# Establishing a human co-infection challenge study of Streptococcus pneumoniae (Spn) and live attenuated influenza vaccine (LAIV) in older healthy adults: Exploring Co-infection with Live Attenuated Influenza Vaccine and PneumococcuS in healthy oldEr adults (ECLIPSE), a UK-based single-centre, single-blind randomised controlled trial

**DOI:** 10.1136/bmjopen-2026-118632

**Published:** 2026-10-12

**Authors:** Rebecca I Jackson, Oisin Hennigan, Karen Kelleher, Carla Solórzano, Elena Mitsi, Ella Morey, Thejaswini Madupuri, Nisha Singh, Hannah Baughan, Bhumika Patel, Nile Verleur, Tatiana Codreanu, Filora Elterish, Ebrima Comma, Yama F Mujadidi, Emma Plested, Hannah Robinson, Sanjita Brito-Mutunayagam, Xinxue Liu, Arnaud M Didierlaurent, Britta C Urban, Katrina M Pollock, Daniela M Ferreira

**Affiliations:** 1 Department of Paediatrics, Oxford Vaccine Group, University of Oxford, Oxford, UK; 2 Department of Clinical Sciences, Liverpool School of Tropical Medicine, Liverpool, UK; 3 NIHR Oxford Biomedical Research Centre, Oxford, UK; 4 Department of Pathology and Immunology, University of Geneva, Geneva, Switzerland

**Keywords:** IMMUNOLOGY, INFECTIOUS DISEASES, GERIATRIC MEDICINE, Research Design, RESPIRATORY MEDICINE (see Thoracic Medicine)

## Abstract

**Introduction:**

Secondary bacterial pneumonia following influenza is a significant cause of morbidity and mortality in older adults. The *E*xploring *C*o-infection with *L*ive Attenuated *I*nfluenza Vaccine and *P*neumococcu*S* in healthy old*E*r adults study investigates how the order of exposure to *Streptococcus pneumoniae* serotype 6B (Spn6B) and live attenuated influenza vaccine (LAIV) affect mucosal immunity, pathogen load and respiratory symptoms in adults over 55 years of age. Immunisation with LAIV is a safe alternative to nasal challenge with live influenza virus because it is cold-adapted and cannot replicate in the lower respiratory tract, reducing the risk of disease in older study participants.

**Methods and analysis:**

This is a single-centre, single-blind randomised controlled human co-infection study (protocol V.4.2, 20 January 2026). Up to 60 healthy adults aged between 55 and 80 years will be randomised 1:1 for intranasal immunisation with LAIV first followed by inoculation with Spn6B or intranasal inoculation with Spn6B first followed by immunisation with LAIV with a 3-day interval between exposures. Throughout the study, we will collect samples from the nose, throat, saliva and blood to analyse how the immune system responds. Participants will also complete a daily symptom diary. Participants will collect saliva and nasosorption samples at home.

A control cohort of up to 20 participants (up to 10 adults aged 18–49 years and up to 10 adults aged 55–80 years) will enable age-stratified comparisons of viral load and mucosal immune responses to immunisation with LAIV only. This cohort will not be randomised.

The primary outcome is feasibility of the study and the safety of participants. The secondary outcomes include respiratory symptoms, how the order of exposure affects pneumococcal carriage, LAIV viral load and nasal immune responses over time as well as the impact of asymptomatic infection with other respiratory pathogens at baseline on these parameters. The study will improve our understanding of immune responses to co-infection with bacterial and viral pathogens in the upper respiratory tract in older adults and why they are at increased risk of pneumonia.

**Ethics and dissemination:**

Ethical approval for this study has been obtained from the East Midlands–Leicester South Research Ethics Committee (REC reference 24/EM/0200). The results will be disseminated via publication in a peer-reviewed journal and presentation at international conferences.

**Trial registration number:**

ISRCTN13284643.

**STRENGTHS AND LIMITATIONS OF THIS STUDY**

- Strict inclusion and exclusion criteria will help ensure safety of the participants and their close contacts and integrity of the study data.
- The timing, dose and strain of pneumococcal inoculation are standardised, reducing variability compared with natural infection.
- The live attenuated influenza vaccine (LAIV)-only cohort is non-randomised and has a small sample size that may limit age-stratified comparisons.
- Participants who decline pneumococcal inoculation may be recruited into the LAIV-only cohort, which may result in selection bias and could affect between-group comparisons.
- Restricting participation to healthy older adults may impact the generalisability of results across populations with respiratory disease and other comorbid conditions.

## Background and rationale

### Pneumococcal disease

*Streptococcus pneumoniae* remains a leading cause of community-acquired pneumonia (CAP) and invasive pneumococcal disease (IPD), contributing to significant global morbidity and mortality. CAP affects up to 14 per 1000 adults annually and contributes to an estimated 4 million deaths worldwide each year.^1^ Pneumococcal disease disproportionately affect*s* young children and the elderly.^2 3^

Pneumococcus is an encapsulated Gram-positive bacterium with over 100 capsular serotypes described. Colonisation of the nasopharynx occurs in 10%–20% of healthy adults, although colonisation rates are lower in older adults. It is typically asymptomatic and is a prerequisite for transmission and disease. Pneumococcal conjugate vaccines (PCV), which target the capsular polysaccharide capsule of up to 20 serotypes, have been shown to protect against mucosal colonisation and disease in children.^4 5^ Indirect protection of unvaccinated adults has been observed due to reduced transmission from vaccinated children.^6^ However, this has been accompanied by a relative increase in colonisation and disease caused by non-vaccine serotypes, a phenomenon known as serotype replacement.^7 8^

### Viral-bacterial interactions

Co-infection with respiratory viruses, particularly influenza, is a recognised risk for secondary bacterial pneumonia. Analyses by Weinberger *et al* showed an association between peaks in influenza activity and subsequent paediatric admissions for IPD.^9 10^ Similarly, healthy children vaccinated with the live attenuated influenza vaccine (LAIV) showed a temporary increase in pneumococcal colonisation.^11^

During the COVID-19 pandemic, widespread public health initiatives such as social restrictions and face-mask use led to a marked decline in seasonal viral infections, including influenza. While carriage rates of *S. pneumoniae* remained largely unchanged, cases of CAP and IPD dropped significantly.^12^ These epidemiological observations support an association between respiratory viral infections and the incidence of pneumococcal disease.

The experimental human pneumococcal challenge (EHPC) model is a type of controlled human infection model (CHIM) that offers a powerful and safe approach to studying respiratory pathogens in a controlled setting. The CHIM using Spn6B, an EHPC model, has been safely used in the UK for 13 years. Between 40% and 70% of participants nasally inoculated with pneumococcus become colonised for 1–4 weeks.^13^ This work has demonstrated that carriage boosts pneumococcus-specific IgG responses as well as cellular responses in the lungs and blood.^14^ Serotype-specific memory B cells were associated with protection from carriage acquisition following challenge studies.^13 15 16^

The EHPC model has been used previously in younger adults to study co-infection with intranasal LAIV and pneumococcus. It has been shown that the order of pathogen exposure alters immune responses and the rates of pneumococcal colonisation.^17 18^ By contrast, pneumococcal colonisation at the time of LAIV vaccination reduced nasal inflammatory responses, leading to decreased antibody responses against influenza virus in the nasal mucosa and lungs.^19^

### Immune response and age

Older adults remain disproportionately affected by pneumococcal disease, even though the colonisation rates and density in the upper respiratory tract are comparable to or lower than those observed in younger adults.^5 20^ This paradox likely reflects age-related changes in both innate and adaptive immunity. Innate immune changes in older adults include impaired neutrophil recruitment into tissue, reduced phagocytosis and bacterial killing and reduced antigen presentation by myeloid cells.^21^

In the adaptive response, ageing is linked to an increase in cytotoxic CD4+ T cells, CD8+ T cells and age-associated B cells in peripheral circulation.^22–24^ Our recent work demonstrates that older adults prone to pneumococcal colonisation show increased inflammation in the nasal mucosa. We hypothesise that nasal inflammation results in tissue injury and facilitates adhesion of pneumococcus.^25^

### Study rationale

*S. pneumoniae* is a leading cause of morbidity and mortality for older adults, who remain vulnerable despite successful pneumococcal and influenza vaccination strategies. Co-infection with respiratory viruses, particularly influenza, is a key contributor to pneumococcal disease. EHPC studies in younger adults show that the order of exposure to LAIV and pneumococcus affects colonisation dynamics and immune responses. However, comparable data in the context of immunosenescence are lacking.

The *E*xploring *C*o-infection with *L*ive Attenuated *I*nfluenza Vaccine and *P*neumococcu*S* in healthy old*E*r adults (ECLIPSE) study extends the LAIV-EHPC co-infection model to older adults to investigate how pathogen order influences mucosal and systemic immunity. We will recruit a group of older adults aged between 55 and 80 years. Participants will be randomised for inoculation with Spn first and LAIV 3 days later, or LAIV followed by Spn after 3 days. In parallel, a non-randomised control group of younger adults (aged 18–49 years) and older adults (aged 55–80 years) will receive LAIV only. This group will allow us to distinguish the impact of LAIV vaccination alone from co-infection with Spn6B on mucosal and systemic immune responses in exploratory analyses.

By comparing older and younger adults, and through comprehensive sampling (nasal mucosa, peripheral blood and lymph node biopsy), this study will provide a detailed picture of immune response at different ages. We hope the findings will inform the design of mucosal vaccines and targeted intervention to reduce infection risk in older at-risk populations.

## Study aims and objectives

This study aims to investigate pathogen interaction dynamics and immune responses in a controlled human co-infection model with Spn6B and LAIV. The primary objective of this study is to assess the feasibility and safety of this model in older adults. Secondary objectives aim to evaluate how the order of exposure affects respiratory symptoms, pneumococcal carriage dynamics, LAIV viral load and nasal immune responses at specific time points. A full list of objectives and outcomes is outlined in online supplemental appendix 3.

## Study design

This is a single-blind, randomised controlled human co-infection study conducted in Oxford using a previously established model.^17^ Eligible healthy older adults will receive an intranasal challenge with LAIV or saline on day –3, followed by pneumococcal challenge (Spn6B) on day 0. The alternate intranasal challenge will be given on day 3 (ie, those who receive LAIV on day −3 will receive saline on day 3 and vice versa). All study participants will be blinded to the order of administration of the challenge agents (figure 1).

**Figure 1:**
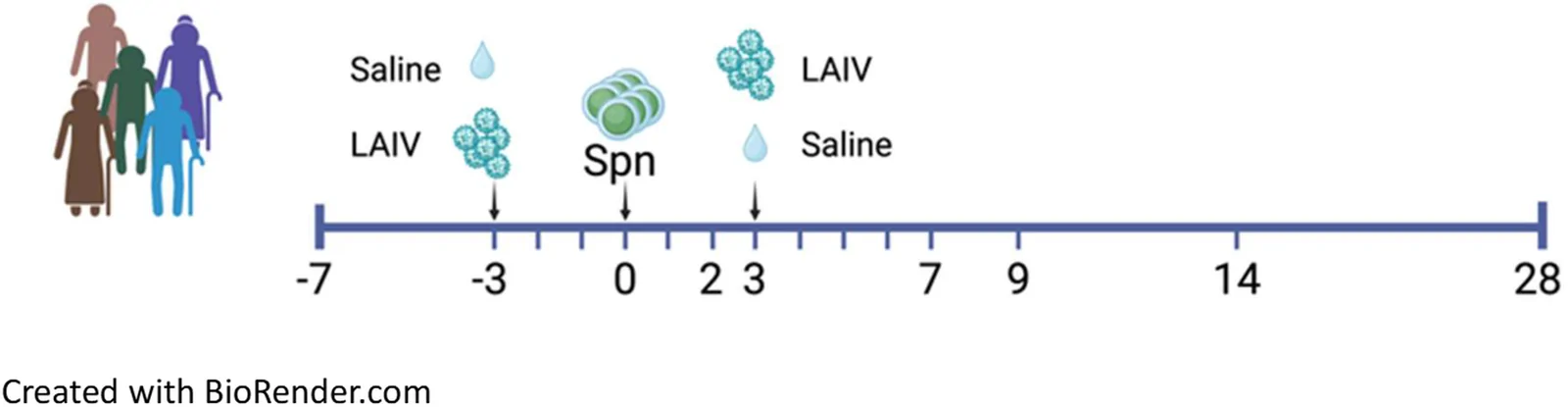
Study design and visit timelines for pneumococcal challenge cohort. LAIV, live attenuated influenza vaccine; Spn, *Streptococcus pneumoniae*.

The planned start date for the trial was 1 September 2024, with the study planned to end by 31 January 2028.

Safe experimental human challenge models involving pneumococcal inoculation have been conducted previously, and this is an established outpatient model.^12^

In addition to the co-infection arm, a control group of younger and older adults will receive intranasal LAIV alone (figure 2). Data from the control group will allow us to investigate immune responses to LAIV in the presence and absence of pneumococcal co-infection. These findings can be compared with existing data generated during Spn6B challenge alone or Spn6B/LAIV co-infection in younger adults.^17 18^ Comparisons involving this cohort are exploratory and may be limited by the allocation procedure and small sample size.

**Figure 2:**
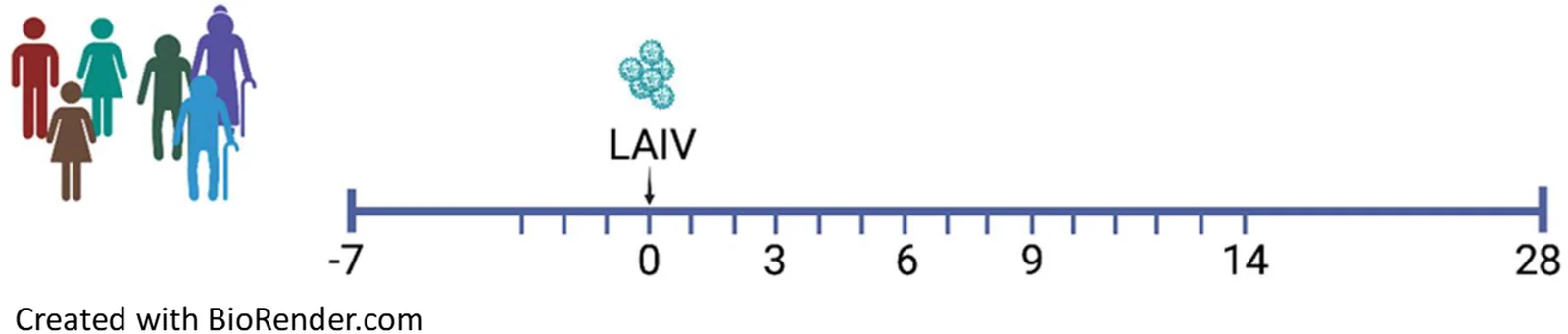
Study design and visit timelines for LAIV-only cohort. LAIV, live attenuated influenza vaccine.

For details on study assessments and procedures, the schedule of events can be found in online supplemental tables 1 and 2 and The Standard Protocol Items: Recommendations for Interventional Trials (SPIRIT) checklist and full protocol are also available in online supplemental appendices 4 and 5, respectively.

### Recruitment, screening and eligibility assessment

This study will recruit healthy older adults (aged 55–80 years), with a control subset of younger (aged 18–49 years) and older adults (aged 55–80 years). Recruitment advertisements will use approved text and will be widely distributed.

They will be directed to read the Participant Information Sheets to undergo a screening process, when they will sign the relevant informed consent form. Participants will be separately consented for their samples to be transferred to the Oxford Vaccine Centre Biobank. Participants will be reimbursed for their time, travel and inconvenience.

Following informed consent, the participant will undergo an eligibility assessment. To minimise risk, we aim to recruit healthy older adults without significant comorbidities (particularly respiratory disease and immunodeficiency) and limited contact with vulnerable populations. To reduce potential confounding factors, we also excluded participants who have had recent influenza vaccine. The inclusion and exclusion criteria are outlined in box 1. Separate temporary exclusion criteria are reviewed at the points of inoculation, lymph node biopsy and nasal biopsy (box 1). Informed consent materials are included in online supplemental appendices 1 and 6.

Box 1 **Schedule of events—inclusion and exclusion criteria**

**Inclusion criteria**

- Participant is willing and able to give informed consent for participation in the study.
- Healthy adults, aged between 55 and 80 years or 18 and 49 years (group B only).
- In the investigator’s opinion, is able and willing to comply with all study requirements.
- Fluent spoken English—to ensure a comprehensive understanding of the research study.
- Willing to allow their general practitioner and consultant, if appropriate, to be notified of participation in the study.
- Agree to provide their National Insurance/Passport number for the purposes of TOPS registration and for payment of reimbursement expenses.
- Participant must live near to the study site or in the surrounding area.
- Females of childbearing potential with a negative urine pregnancy test at screening who are willing to practice adequate contraceptive measures as per the UK Clinical Trial Facilitation Group requirements during the study.

**Exclusion criteria**

- Severe and/or uncontrolled cardiovascular disease, respiratory disease, gastrointestinal disease, liver disease, renal disease, endocrine disorder or neurological illness, as judged by the investigator (note, mild/moderate well-controlled comorbidities are allowed). Including but not limited to:
  - Asplenia or dysfunction of the spleen.
  - Chronic respiratory disease (eg, asthma requiring medication, including a salbutamol inhaler, within the last 12 months, chronic obstructive pulmonary disease, bronchiectasis or sleep apnoea).
  - Chronic heart disease (eg, angina, ischaemic heart disease, chronic heart failure)—controlled and stable hypertension may be included.
  - Severe chronic kidney disease (eg, nephrotic syndrome, kidney transplant or requiring dialysis).
  - Chronic liver disease (eg, cirrhosis, biliary atresia, hepatitis).
  - Chronic neurological disease that limits mobility, bulbar or respiratory function (including stroke, Parkinson’s disease, dementia and multiple sclerosis).
  - Diabetes mellitus (including diet-controlled).
  - Receipt of immunosuppressive therapy, such as anticancer chemotherapy or radiation therapy, within the preceding 12 months, or long-term systemic corticosteroid, Roaccutane or disease-modifying antirheumatic drugs therapy for >7 consecutive days within the 3 months prior to enrolment.
  - Individuals with cochlear ear implants.
  - Individuals with major cerebrospinal fluid leaks (eg, following traumatic or major skull surgery, or requiring cerebrospinal fluid shunts).
  - Subjects with known or suspected immune deficiency (eg, known IgA deficiency, immotile cilia syndrome or Kartagener’s syndrome).
  - History of frequent nosebleeds.
  - Bleeding disorders.
  - No major pneumococcal illness requiring hospitalisation in the last 10 years.

- Maternal (group B)
  - Female participants who are pregnant.
  - Female participants who are lactating.
  - Female participants who intend to become pregnant during the study.
  - Female participants unable to take contraception measures during the study (from consent to final study visit).
- Close contact with individuals at increased risk of pneumococcal disease (eg, children under 5 years, immunocompromised individuals) (group A only).
- Healthcare workers in a direct caring role for individuals at increased risk of pneumococcal disease (group A only).
- On medication that may affect the immune system in any way (eg, steroids).
- Taking long-term antibiotics, nasal/inhaled steroids, oral antiplatelets or warfarin therapy.
- Allergy to penicillin, amoxicillin and/or gentamicin (group A only).
- Allergy to gelatin, lidocaine or any ingredient of the influenza vaccine.
- People who currently smoke or vape regularly (daily) or who previously smoked or vaped regularly but stopped <12 months ago (up to 10 pack-years of smoking history allowed).
- History of drug or alcohol misuse (frequently drinking above the recommended alcohol intake limit: men and women should not regularly drink >14 units per week).
- Any clinically significant findings on screening blood tests.
- Unable to make specific inoculation/vaccination visits required for the study, or with overseas travel booked within 21 days following baseline testing.
- Received any influenza vaccine during the same winter season as they are recruited.
- Pneumococcal vaccination (which we can confirm is not the pneumococcal conjugate vaccine (PCV)) in the last 1 year (older adults who have had pneumococcal vaccination but not influenza vaccination may be considered for group B live attenuated influenza vaccine-only cohort).
  - Participants who have received the PCV specifically within the last 3 years (this would not be given routinely in the UK schedule).
- Previous involvement in a clinical trial with pneumococcal inoculation in the last 3 years.
- Scheduled elective surgery or other procedures requiring general anaesthesia during the study.
- Participants who have participated in another research trial involving an investigational product in the past 12 weeks.
- History of significant unexplained bleeding after a surgical or dental procedure (for optional nasal biopsy participants only).
- Any other issue which, in the opinion of the study staff, may:
  - Put the participant or their contacts at risk because of participation in the study.
  - Adversely affect the interpretation of the study results, or impair the participant’s ability to participate in the study.

**Temporary exclusion for inoculation**

- Current acute infective illness—delay inoculation by 14 days.
- Asymptomatic positive COVID-19 lateral flow test (taken on day of planned challenge)—delay inoculation by 21 days.
- Recent/Current upper respiratory tract infection or antimicrobial (including antiviral) use—delay inoculation by 28 days after last day of illness or last date of antimicrobial therapy.
- Received any other vaccination within the past 28 days.
- Any study participant who is colonised with pneumococcus at the baseline visit (day –7) will be temporarily excluded for 30 days, after which pneumococcal colonisation will be reassessed.
- Temporary use of oral or inhaled steroids (within a month prior to enrolment).

**Temporary exclusion for lymph node biopsy**

- The following are temporary exclusion criteria applicable to participants who have consented to lymph node biopsy (this is optional). On day of lymph node biopsy, the following criteria will be reviewed:
  - Any medicine that increases bleeding risk taken in the past 7 days (eg, aspirin).
  - Signs of local infection or rash at the site of intended biopsy.

**Temporary exclusion for nasal biopsy**

- Antibiotic use (during the study)—delay nasal biopsy for at least 1 week from last date of therapy.
- Dental infections—delay nasal biopsy for at least 2 weeks after last day of illness.

### Allocation

Participants in group A will involve up to 60 participants randomised 1:1 to receive saline on day –3, Spn6B inoculation on day 0, followed by LAIV on day 3 or to receive LAIV on day –3, Spn6B inoculation on day 0, followed by saline on day 3. Random block sizes of 2 or 4 will be used. Randomisation will occur on the day −7 visit by clinical staff once eligibility has been established. Laboratory staff will be blinded to participant group. A computer-generated randomisation list will be generated by the study statistician. Randomisation software on Research Electronic Data Capture will be used and data will be stored on a secure electronic portal accessible by the statistician and information technology team.

Participants in group B will not be blinded to study procedures or randomised.

Participant allocation to group A or B will not be randomised. Younger adults between the ages of 18 and 49 years will automatically be allocated to group B. When adults between the ages of 55 and 80 years are screened, we will initially assign participants to group A. In the event that a participant does not want to receive pneumococcal inoculation, but is still interested in being a part of the study, we will recruit the participant to group B, although this may introduce some selection bias.

### Blinding

Group A participants will be blinded to LAIV/saline administration on day –3 and day 3. The clinical research teams will not be blinded in this study. There is no blinding involved in group B. Participant blinding is required to reduce the risk of bias in the reporting of adverse events (AEs) depending on the order of administration of LAIV/Spn6B.

Scheduled unblinding for group A will occur after the last participant has completed their final visit. Participant unblinding may also be performed at an earlier time point in the event of study withdrawal or the occurrence of serious adverse events (SAEs), serious adverse reactions or suspected unexpected serious adverse reactions.

## Study interventions

### Inoculation with *S. pneumoniae* serotype 6B

A mid-log broth culture of Spn6B will be frozen at −80°C in aliquots of glycerol-enriched media. Frozen aliquots will be thawed and checked for bacterial number (colony-forming units (CFU) per mL) and purity. These checks will first be carried out in research laboratories and then identification, purity by whole-genome sequencing and penicillin sensitivity will be confirmed in a reference laboratory (UK Health Security Agency). On experimental inoculation days, aliquots will be thawed, washed twice and resuspended in 0.9% normal saline at the correct density.

Once the inoculum is prepared, it will be administered within 30 min to avoid degradation. We will aim for a dose of 80 000 CFU/100 μL per naris of the Spn6B inoculum, as determined from previous challenge studies (allowing for variation between half and double this dose).

The study clinician will review the inclusion/exclusion criteria, temporary exclusion for challenge and clinical stability of the participant. Study procedures will be carried out as listed in online supplemental table 1. Before challenge, a viral swab COVID-19 will be performed using a point-of-care test to confirm that the participant is negative before inoculation.

The inoculum will be administered according to the local standard operating procedure (SOP). The participant will be seated in a semi-recumbent position. Using a P200 micropipette, 0.1 mL of pneumococcus-containing fluid will be instilled into each nostril. This will be done slowly, with a sufficient interval between each inoculation (2–3 min) to ensure maximum contact time with the nasal and pharyngeal mucosa. After inoculation, the participant will remain in this position for up to 15 min. Participants will be asked not to wash or blow their noses for at least 1 hour. After inoculation, participants will be given a safety pack containing a safety leaflet, medical alert card, thermometer and a supply of amoxicillin. They will be advised to complete a symptom diary and given an emergency contact number. Participants may be invited to attend an additional or unscheduled visit for further clinical examination if they show signs of pneumococcal infection or warrant clinical review.

### Vaccination with LAIV/saline administration

#### Day –3 and day 3 (group A) and day 0 (group B)

During this visit, the study clinician will review the inclusion/exclusion criteria, screen for AEs and check vitals. Study procedure will be carried out as listed in online supplemental tables 1 and 2.

Once eligibility is confirmed, the study nurse should prepare the LAIV/saline out of view of the participant. The participant is told to breathe normally during administration and half the dose is administered to each nostril. The participant is monitored for 15 min postprocedure.

Fluenz Tetra, Fluenz Trivalent and FluMist Quadrivalent are licensed medicinal products in the UK for children under the age of 18 years. In this study, they are being used outside their licensing indications. However, existing data from previous trials support their safety in this population and have demonstrated an acceptable safety profile in older adults. Reported AEs were predominantly mild, self-limiting upper respiratory symptoms, without an increased risk of vaccine-related SAEs or mortality compared with placebo or inactivated influenza vaccine.^26 27^ We will administer the composition that is recommended for each influenza season.

Participants in both groups will attend follow-up visits outlined in the schedule of events (online supplemental tables 1 and 2). These visits will include a review of AEs, clinical status and study procedures discussed below.

## Study procedures

Blood sampling will be performed by trained, experienced staff for safety monitoring (eg, full blood count) and immunological assays. This includes serum immunoglobulins, peripheral blood mononuclear cell populations and host RNA expression.

Microbiological and molecular sampling will include several procedures as outlined in online supplemental tables 1 and 2. Plans for collection, laboratory evaluation and storage of biological specimens are additionally outlined in. Online supplemental appendix 2 Colonisation will be assessed by classical microbiological culture in nasal washes, with serotype determined as Spn6B by latex agglutination. Colonisation results will also be confirmed by multiplex quantitative PCR for *lytA* and *6BcpsA* genes in nasal wash pellets. A participant will be considered positive for colonisation if they test positive for pneumococcus at any nasal wash time point by either classical microbiological or molecular methods. Nasopharyngeal samples for COVID-19 will be taken prior to study intervention. Serial throat swabs will be performed in clinic prior to nasal wash for bacterial analysis and viral PCR.

This study uses clinic-based and home-based sampling to monitor mucosal immune responses. Nasosorption is performed to collect nasal lining fluid for cytokine analysis and to analyse the kinetics of viral load. Concurrent saliva samples are also collected at home for comparison. In-clinic nasal cell sampling enables characterisation of local immune populations and allows analysis of adaptive T-cell and B-cell responses using single-cell sequencing and functional assays.

A symptom diary will collect AEs for 20 days following the first study intervention. This will focus on upper and lower respiratory tract symptoms (online supplemental appendix 5).

### Fine-needle aspirate (optional)

This is an optional procedure performed on a subset of participants across groups A and B. Eligibility will be assessed prior to the procedure, see box 1 for full criteria. Aspiration of axillary lymph nodes will be performed under aseptic conditions with ultrasound guidance by a trained member of staff, as previously outlined.^28 29^ This can occur at an unscheduled visit, and a paired blood sample will also be collected at the same visit for comparison.

### Nasal biopsy (optional)

Nasal biopsy tissue will be collected on a single occasion as an optional procedure. This tissue will be collected from the inferior turbinate and/or the postnasal space/adenoid following National Health Service procedures. Participants will have up to four biopsies collected, performed by a trained ear, nose and throat surgeon in an acute hospital outpatient setting using local anaesthesia.

### Statistical methods

Analyses will be reported in accordance with the Consolidated Standards of Reporting Trials (CONSORT) guidelines. The primary objective of the study is to assess the feasibility and safety of the co-infection human challenge model in older adults. Safety outcomes, including AEs and respiratory symptoms, will be summarised descriptively by study group.

The primary biological analyses will focus on longitudinal immune responses within the nasal mucosa, reflecting the principal site of pathogen interaction. Peripheral blood immune responses will be analysed as complementary systemic comparators to investigate compartmentalisation of mucosal and systemic immunity.

Continuous immunological measurements are anticipated to be right-skewed and will be log-transformed where appropriate. Summary statistics will include geometric mean concentrations or geometric mean titres, together with corresponding 95% CIs. Outcomes will be summarised by treatment group and study visit.

Longitudinal outcomes will be analysed using repeated-measures regression models (eg, mixed-effects models where appropriate) that account for within-participant correlation over time. Models will include treatment group, study time point and their interaction, with participant included as a random effect where appropriate. Baseline measurements and other clinically relevant covariates will be incorporated into analyses where appropriate.

Pneumococcal carriage and density, LAIV viral load and other longitudinal microbiological outcomes will be analysed using regression methods appropriate for the distribution of each endpoint. Estimates of treatment effects will be presented with 95% CIs.

Asymptomatic respiratory viral infection at baseline has previously been shown to influence pneumococcal carriage acquisition and density and will therefore be included as a variable in the exploratory analyses. Detection of naturally acquired respiratory pathogens in baseline samples or during follow-up will not result in automatic exclusion from the primary analyses. These infections will be recorded and considered in exploratory sensitivity analyses or incorporated as covariates where appropriate to assess their potential influence on microbiological and immunological outcomes.

Single-cell RNA sequencing and other high-dimensional immunological datasets will be analysed as exploratory, hypothesis-generating endpoints, with emphasis on identifying longitudinal changes in immune cell populations, transcriptional programmes and cellular states within the nasal mucosa. Analyses will focus on biological interpretation and generation of mechanistic hypotheses rather than formal hypothesis testing of individual genes. Where appropriate, false discovery rate methods will be applied to high-dimensional datasets to account for multiple testing.

The per-protocol population will be used for the primary immunological analyses. Safety analyses will include all participants receiving at least one study intervention. Reasons for missing data will be reported; missing observations will not be imputed. Longitudinal models will use all available observations under the assumption that data are missing at random.

### Sample size determination

Group A will recruit up to 60 healthy older adults aged 55–80 years, with recruitment continuing until at least 40 participants (approximately 20 per randomised arm) have completed the study. This allows for anticipated screen failures, withdrawals and participants with pre-existing pneumococcal carriage or other reasons for non-evaluable datasets.

The study is designed primarily as a mechanistic experimental medicine study to assess the feasibility and safety of the co-infection challenge model rather than to provide definitive hypothesis testing for immunological endpoints. Accordingly, the sample size was determined by feasibility, safety considerations, experience from previous EHPC studies and the need to generate sufficiently rich longitudinal datasets from repeated mucosal and systemic sampling.

As this is a mechanistic experimental medicine study, the sample size was determined primarily by feasibility, participant safety, previous experience with the EHPC model and earlier LAIV-pneumococcal co-infection studies, rather than formal hypothesis testing of the secondary immunological endpoints. The repeated longitudinal sampling design provides multiple within-participant measurements across mucosal, microbiological and systemic compartments, enabling detailed characterisation of temporal immune responses and pathogen dynamics despite the relatively modest cohort size. The study is intended to generate mechanistic hypotheses, estimate biological variability and inform the design and sample size calculations of future confirmatory studies.

Group B will include up to 20 participants (7–10 younger adults and 7–10 older adults). This comparator cohort is intended to characterise immune responses to LAIV alone and enable exploratory comparisons with the co-infection groups. Comparisons involving group B will therefore be considered exploratory and interpreted accordingly.

## Ethics and dissemination

### Safety reporting

AEs will be collected using participant electronic diaries for 21 days postinoculation. Any significant deviation in laboratory results or vital signs will be reported as an AE. All AEs will be assessed by a study investigator using predefined grading scales for severity and causality (table 1). The participants will have a 24-hour contact number available for AE reporting, and all grade 3 solicited AEs will be followed up with a clinical review.

**Table 1:** Assessment of causality of AEs

Not related	No temporal relationship to LAIV or *Streptococcus pneumoniae* inoculation and Alternative aetiology (clinical, environmental or other intervention), and Does not follow pattern of recognised response to LAIV or *S. pneumoniae* inoculation or other study procedure
Possible	Reasonable temporal relationship to LAIV or *S. pneumoniae* inoculation, or Event not readily explained by alternative aetiology (clinical, environmental or other interventions), or Similar pattern of response to that seen with LAIV or *S. pneumoniae*
Probable	Reasonable temporal relationship to LAIV or *S. pneumoniae* inoculation or other study procedure, and Event not readily produced by alternative aetiology (clinical, environment or other interventions), or Known pattern of response with LAIV or *S. pneumoniae* or other study procedure.
Definite	Reasonable temporal relationship to LAIV or *S. pneumoniae* inoculation or other study procedure, and Event not readily produced by alternative aetiology (clinical, environment or other interventions), and Known pattern of response to LAIV or *S. pneumoniae* or other study procedure

AE, adverse event; LAIV, live attenuated influenza vaccine.

SAEs will be collected from enrolment through to study completion. IPD, prolonged bacterial carriage and medically attended events related to study interventions are classified as adverse events of special interest (AESIs). SAEs and AESIs will be reported to the principal investigator, sponsor and relevant regulatory bodies according to the study timelines.

### Data collection and management

Each study participant will have a unique participant ID allocated at the screening visit. Study data are stored electronically on a secure server, and paper notes are kept in a secure location at each study site or as outlined in local SOPs for up to 25 ears. Participants’ bank details will be stored for a minimum of 7 years. Volunteers who complete online screening only will not have data kept beyond the end of the study.

Names or identifying details are not included in any electronic file containing study data, except in specific circumstances where consent has been given. Participant’s personal information will be stored on a separate database not linked to the clinical database. Further information on data storage and the relational database used for the electronic data capture system is available in the main protocol (online supplemental appendix 5). The findings will be disseminated through peer-reviewed journals and relevant scientific conferences. All publications will adhere to the CONSORT guidelines and the requirements of the International Committee of Medical Journal Editors.

### Auditing

Direct access will be granted to authorised representatives to permit study-related monitoring, audits and inspections. The Data and Safety Monitoring Committee (DSMC) will oversee the study and may review data in the interest of safety. Weekly safety summaries will be provided to the DSMC; these reports will include details such as carriage rate, AEs, AESIs and SAEs.

Interim data will be provided if, at any time, the Study Management Group has any concerns regarding the safety of a participant or the general public. The DMSC will advise the study investigators on whether there are any ethical or safety reasons regarding the study. There are defined stopping rules that may result in a participant being withdrawn from the study.

Solicited AEs: the participant develops a grade 3 systemic solicited AE considered possibly, probably or definitely related within 2 days after challenge (the day of challenge and one subsequent day) which persists for 48 hours or is deemed severe by clinician assessment.

Unsolicited AEs: the participant has a grade 3 AE, considered possibly, probably or definitely related to challenge that persists for 48 hours or is deemed severe by clinician assessment, or has a SAE considered possibly, probably or definitely related to challenge.

The chief investigator (CI), with the DSMC, will have the right to terminate the study at any time on grounds of participant safety.

The CI shall submit an Annual Progress report once a year throughout the study, or on request, to the host organisation, sponsor and funder (where required). In addition, an end of study notification and final report will be submitted to the same parties.

### Ancillary and post-trial care

The investigator may choose to withdraw a participant from the study in the event of safety concerns or non-compliance with study procedures. Participants should continue to attend the follow-up visits unless they withdraw consent. Examples for withdrawal include, but are not limited to, SAEs, AEs, new medications (eg, intercurrent antibiotics) and ineligibility. If the participant is withdrawn due to an AE, the investigator may arrange follow-up visits or telephone contact until the event resolves.

Withdrawal from the study will not result in exclusion of existing data generated by the participant from analysis. Participants can request that their samples are destroyed at any point during or after the study, although data that have already been generated from samples analysed up to that point will be retained. The reason for withdrawal, if given, will be recorded in the electronic case report form. If a participant is withdrawn due to an AE, the investigator may arrange for follow-up visits or telephone calls until the adverse event has resolved or stabilised.

Participants in group A will be provided with 3 days of amoxicillin 500 mg three times a day to reduce density of pneumococcal colonisation if they remain positive at the last two face-to-face visits. Participants will also be advised to complete a course of amoxicillin if they are unwell, withdraw from the trial after Spn inoculation or at the clinician’s discretion.

Participants will be offered the seasonal COVID-19 and influenza vaccines, if eligible, at their final visit after completing the study. This is to prevent undue delay of routine immunisations for at-risk groups.

### Research ethics approval

The investigator will ensure that the study is conducted in accordance with the principles of the Declaration of Helsinki, with relevant regulations and with Good Clinical Practice CH E6(R2) and latest versions, where applicable.

Following sponsor approval by the University of Oxford, the protocol, informed consent form, participant information sheet and required material has received approval from the East Midlands–Leicester South Research Ethics Committee (REC reference 24/EM/0200). The investigator will submit and, where necessary, obtain approval from the above parties for all substantial amendments to the original approved documents.

### Patient and public involvement

A patient and public involvement and engagement group reviewed participant-facing materials, including information leaflets and recruitment advertisements. Their suggestions were incorporated into these materials to ensure clarity and accessibility for potential participants. This group will also support advertising strategies, recruitment planning and public engagement initiatives.

Additionally, a regular newsletter disseminating study results and updates on ongoing trials will be sent out to individuals who opt to receive it. Social media platforms will also be used to engage followers with real-time updates about the research.

### Limitations

CHIM studies, including the EHPC model, can provide important insights into the pathophysiology of pneumococcal infection; however, several limitations should be considered. Standardisation of the timing, dose and strain of pneumococcal inoculation reduces the variability when compared with natural infection, where individuals may experience multiple exposures over prolonged time frames. By restricting participation to healthy older adults, the results may not be generalisable across populations that have a higher risk of severe infection, including young children and individuals with comorbidities. The LAIV-only cohort is non-randomised and has a small sample size, which may limit age-stratified and between-group comparisons. Additionally, participants who decline pneumococcal inoculation may be recruited into the LAIV-only cohort, introducing potential selection bias and further limiting the interpretation of comparisons between groups.

## Supplementary material

10.1136/bmjopen-2026-118632 online supplemental table 1

10.1136/bmjopen-2026-118632 online supplemental table 2

10.1136/bmjopen-2026-118632 online supplemental appendix 1

10.1136/bmjopen-2026-118632 online supplemental appendix 2

10.1136/bmjopen-2026-118632 online supplemental appendix 3

10.1136/bmjopen-2026-118632 online supplemental appendix 4

10.1136/bmjopen-2026-118632 online supplemental appendix 5

10.1136/bmjopen-2026-118632 online supplemental appendix 6
