# Supplementary material for: Establishing a human co-infection challenge study of Streptococcus pneumoniae (Spn) and live attenuated influenza vaccine (LAIV) in older healthy adults: Exploring Co-infection with Live Attenuated Influenza Vaccine and PneumococcuS in healthy oldEr adults (ECLIPSE), a UK-based single-centre, single-blind randomised controlled trial
Source: BMJ Open. 2026 Oct 12;16(10):e118632. doi: 10.1136/bmjopen-2026-118632 (PMC13662028; doi:10.1136/bmjopen-2026-118632)
Supplement: Supplementary file 2 [file bmjopen-16-10-s004.pdf]

Table of Contents

Table of Contents 1
1 ABBREVIATIONS 2
2 REVIEW HISTORY 3
3 OVERVIEW OF STUDY AND SCOPE OF WORK 3
3.1 Study Overview 3
3.2 Purpose and Scope of Laboratory Analysis Plan 4
3.3 Endpoints, related analysis methods and their principles 4
3.3.1 Primary Objective 4
3.3.2 1.3.2 Secondary Objectives 4
3.3.3 1.3.3 Exploratory Objectives 5
4 ROLES, RESPONSIBILITIES AND TIME POINTS 6
4.1 Laboratory Communication 8
5 ANALYTICAL PROCESSES 9
5.1 Challenge Preparation 9
5.2 Clinic Collection Sample Processing 9
5.2.1 Clinic Sample Transport Conditions and Processing Time Window 12
5.3 Home-Collected Samples 13
5.4 Sample Analysis 14
5.5 Equipment and Reagents 21
5.5.1 Non-commercial reagent requirements 21
5.6 Sample Shipment and Long-Term Storage 21
5.6.1 Local Sample storage 22
5.6.2 Sample Shipment and External Storage 23
5.6.3 Disposal of samples 23
6 DATA MANAGEMENT 23
7 QUALITY CONTROL AND QUALITY ASSURANCE 25
8 REFERENCES 25

1 ABBREVIATIONS

ASAP	As Soon As Possible
CAP	Community-Acquired Pneumonia
CI	Chief Investigator
CRF	Case Report Form
EHPC	Experimental Human Pneumococcal Carriage
GCP	Good Clinical Practice
GP	General Practitioner
HRA	Health Research Authority
ICF	Informed Consent Form
IPD	Invasive Pneumococcal Disease
LAIV	Live Attenuated Influenza Vaccine
NHS	National Health Service
NW	Nasal Wash
OM	Otitis Media
PBMC	Peripheral Blood Mononuclear Cell
PCR	Polymerase Chain Reaction
PI	Principal Investigator
PIS	Participant/ Patient Information Sheet
qPCR	Quantitative Polymerase Chain Reaction
REC	Research Ethics Committee
RES	Research Ethics Service
RGEA	Research Governance, Ethics & Assurance Team
RNA	Ribonucleic Acid
SAE	Serious Adverse Event
SOP	Standard Operating Procedure
SPN	Streptococcus pneumoniae
SPN6B	Streptococcus pneumoniae serotype 6B
TDS	Three times a day
TOPS	The Over Volunteering Prevention System database

2 REVIEW HISTORY

Date	Review By	Review Type (minor/major)	Details of Changes	Previous versions
18/11/2024	Britta Urban	Minor	Added sample disposal section	V1.0
08 July 2025	Britta Urban<br>Bhumika Patel	Major	Change to throat sampling media collection type<br>Removal of STGG for Saliva samples<br>Implementation of SA01<br>Change to nasal cell sampling	V1.1
04/08/2025	Nile Verleur<br>Britta Urban	Major	Addition of Nasal biopsy processing	V2.0
12/12/2025	Nile Verleur<br>Britta Urban	Minor	Extension of age range for recruitment	2.1

3 OVERVIEW OF STUDY AND SCOPE OF WORK

3.1 Study Overview

This study intends to investigate the recruitment and activation of cells in the nasal mucosa over the course of infection (challenge with pneumococcus) and vaccination with Influenza in older adults (>55 years). The use of a cold-adapted influenza virus, such as LAIV, will provide a safe platform to study influenza nasal effects without any risk to older adult participants. Considering LAIV leads to profound inflammation in the nose and impaired responses in the nose and lung to pneumococcal carriage in young adults, we will compare this with an older cohort. Since older adults show increased nasal inflammation, we postulate that inflammatory responses due to co-infection will be even more exacerbated in older adults leading to increased virus shedding and pneumococcal density in the nose and oropharynx (throats). In this clinical study, we will extend this LAIV-EHPC co-infection model to the elderly population (aged >55 years) to assess nasal immune responses to LAIV and pneumococcus carriage, as well as the effect of the co-infection in this population. We will conduct two studies under the same protocol. Those studies will reflect alternative scenarios:

1. Colonisation with Pneumococcus first (LAIV administered 3 days after colonisation with pneumococcus)
2. Immunisation first (LAIV precedes nasopharyngeal inoculation with pneumococcus by 3 days).

In parallel, and to obtain data on nasal immune responses to infection with LAIV alone, we will vaccinate a control group of younger and older adults with LAIV and monitor their immune responses in a comparable manner to the co-infection study. These data will allow us to understand which immune responses are triggered by LAIV alone and whether the quality or quantity of immune responses to LAIV or viral shedding differ between younger and older adults.

The results of the study will provide a better understanding of the factors that influence susceptibility to infection with either pathogen and their interaction. We will undertake an in-depth analysis of immune responses in the upper respiratory tract, peripheral circulation and lymph nodes and their association with protection or susceptibility to colonisation/infection. The obtained knowledge will contribute to the design of intervention that could be tailored to an at-risk population of older adults through vaccination and/or early treatment with antivirals. A better understanding of immune response at the nasal mucosa is of particular interest for the design of mucosal vaccines that reduce the risk of dissemination of respiratory pathogens into the lower respiratory tract.

Group A: This will be a single blind randomised controlled human co-infection study using a previously established pneumococcal and LAIV infection model (Jochems et al., 2018). This study will be conducted in Oxford. Eligible healthy, older adults will receive an intranasal challenge with LAIV/saline on Day –3 followed by challenge with Spn6B on day 0. Participants will receive a further intranasal challenge on Day 3 with the LAIV/saline option they did not receive on Day –3 (participants who received saline at day –3 will receive a LAIV on day 3, and those who received LAIV will receive saline, **Figure 1**). All study participants will be blinded to the order of challenge agents (LAIV first or saline first).

Group B: In addition to the co-infection model, we will vaccinate a control group of younger and older adults with intranasal LAIV only to determine the impact on systemic and mucosal immune responses.

Figure 1 Co-infection Challenge Overview

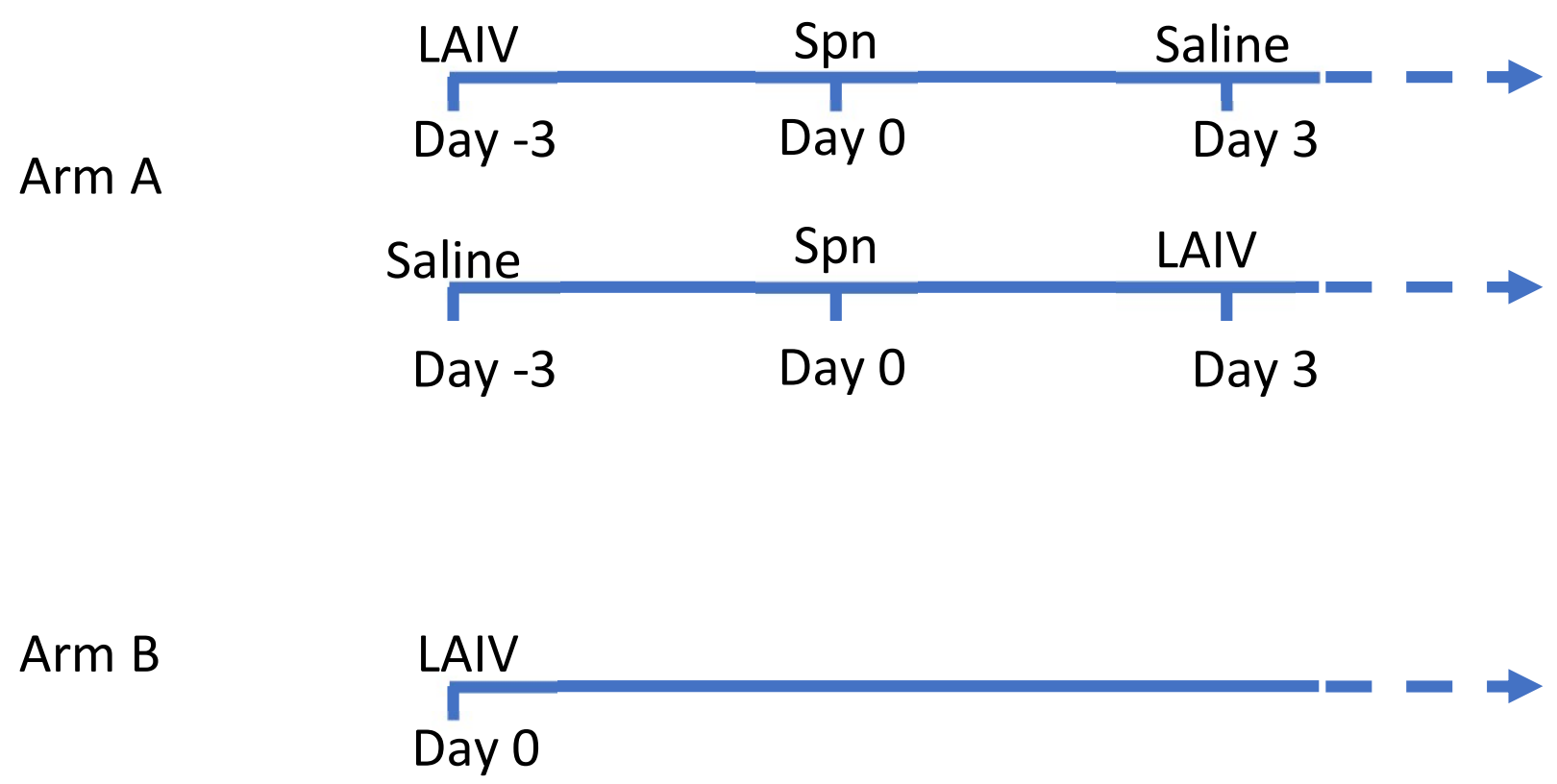

3.2 Purpose and Scope of Laboratory Analysis Plan

This laboratory analysis plan ensures that samples from the current version of the: "ECLIPSE Protocol" can be processed and analysed safely and accurately according to protocol and local needs.

3.3 Endpoints, related analysis methods and their principles

All endpoints are described below. The analysis plan considers only the laboratory aspect of sample analysis

3.3.1 Primary Objective

To determine if a co-infection human challenge model with LAIV and *S. pneumoniae* (Spn6B) is feasible and safe in older adults.

Laboratory Endpoints:

1. Absence of SAEs/AESIs relating to inoculation throughout the study

3.3.2 1.3.2 Secondary Objectives

1. If co-infection with LAIV and Spn6B alter upper and lower respiratory tract infection symptoms

2. How primary LAIV vaccination alters the:
    a. Risk of Spn6B carriage acquisition
    b. Density of Spn6B carriage
    c. Duration of Spn6B carriage
3. How primary Spn6B challenge or carriage alters the:
    a. Risk of secondary LAIV infection (i.e., detection of LAIV)
    b. Viral load of secondary LAIV infection (AUC of density over time)
4. If nasal colonisation of other respiratory viruses and bacteria is detectable
5. To evaluate changes in nasal cells phenotype and function over time

Laboratory Endpoints:

1. Symptomatology using diary cards, with clinical review of LRTI and URTI symptoms at visits
2. Classical culture and molecular methods including RT-qPCR
    a. Colonisation rates of Spn6B carriage
    b. Density of Spn6B carriage
    c. Longitudinal quantification
3. Secondary LAIV infection and viral load determined by RT-qPCR
4. Detection of most common respiratory viruses and other respiratory bacteria will be assessed by qPCR or microfluidic qPCR
5. Analysis of changes in cell populations including antigen-specific T and B cells at different time points in nasal mucosa

3.3.3 1.3.3 Exploratory Objectives

1. To identify if primary Spn6B challenge or carriage alters immune responses to secondary LAIV vaccination (innate and adaptive) and vice-versa.
2. To determine T cell receptor and B cell receptor sequence at the single cell level in nasal cells
3. To evaluate changes in inflammatory markers and antibody responses to pneumococcus and LAIV in the mucosa
4. To compare selected immune parameters both at the cellular and transcriptomic level between the nasal mucosa and systemic circulation
5. Comparison of immune response to LAIV vaccination over the life course (children, younger adults, older adults)
6. Comparison of immune and transcriptional parameters of experimental co-infection with Spn6B and LAIV with data obtained from natural infected older adults (symptomatic and asymptomatic)
7. To determine the frequency, phenotype, and function of immune cells in axillary secondary lymphoid tissue compared to blood and nasal mucosal following intranasal immunisation, in both older and younger adults.
8. To characterise transcriptional changes in immune cells (single cell and bulk RNA sequencing) in response to LAIV and Spn6B challenge/carriage/vaccination.

Laboratory Endpoints:

1. Assessment of immune responses including nasal cytokines, cell populations, Influenza- and Spn6B-specific antibodies and cellular immunity before and after each pathogen challenge.

2. T cell receptor and B cell receptor sequencing in parallel with scRNAseq to determine clonotypes in the nasal mucosa in response to LAIV/Spn6B co-infection
3. Analysis of changes in antibody levels and inflammatory markers at different time points in nasal lining fluid, saliva and/or throat swabs
4. Analysis of antibody level, inflammatory markers and cell populations (cellular and transcriptome level) in systemic circulation and compare data with those generated at the nasal mucosa
5. Meta analysis of data generated in this study with comparable data generated in a parallel study on mucosal immune responses to infection with LAIV in children
6. Meta analysis of data generated in this study with comparable data generated by collaborators in a hospital study (University of Geneva).
7. Single cell ribonucleic acid sequencing (scRNA-seq) to measure cell by cell transcriptomes in lymph node cells and/or multiparameter flow cytometry
8. RNAseq analysis to determine gene induction and regulation to identify gene signatures (cell specific where possible) that correlate with susceptibility to infection, as well as alterations in immune responses and symptoms during co-infection

4 ROLES, RESPONSIBILITIES AND TIME POINTS

Role/Responsibility	Name	Contact Details
Chief Investigator	Professor Katrina Pollock	Oxford Vaccine Group (OVG)<br>University of Oxford<br>Centre for Clinical Vaccinology & Tropical Medicine (CCVTM)<br>Churchill Hospital, Oxford OX3 7LE
Investigators	Professor Maheshi Ramasamy	Oxford Vaccine Group (OVG)
Lead Scientific Investigator	Professor Daniela Ferreira	Oxford Vaccine Group,<br>and<br>Liverpool Vaccine Group, Liverpool School of Tropical Medicine, Accelerator Building, 1 Daulby Street, Liverpool, L7 8XZ
Scientific Investigators	Professor Arnaud Didierlaurent<br>Dr Britta Urban<br>Dr Carla Solorzano-Gonzalez	Centre of Vaccinology and Translational Research in Immunology Department of Pathology and Immunology<br>Faculty of Medicine<br>Rue Michel-Servet 1, CH-1211 Geneve<br>University of Geneva<br>Oxford Vaccine Group,<br>Oxford Vaccine Group,

Sponsor	University of Oxford	Research Governance, Ethics & Assurance (RGEA)<br>Boundary Brook House, Churchill Drive, Headington, Oxford, OX3 7GB
Funder(s)	European Union (grant number: 101080528-NOSEVAC) and Horizon Europe Guarantee Extension. UKRI Reference number: 10077113	UK Research and Innovation<br>Polaris House<br>Swindon<br>SN2 1FL
Clinical Trials Unit	Primary Care and Vaccine Collaborative Clinical Trials Unit	Oxford Vaccine Group,<br>Centre for Clinical Vaccinology and Tropical Medicine (CCVTM),<br>Churchill Hospital Old Road,<br>Headington<br>Oxford<br>United Kingdom<br>OX3 7LE
Trial Sites	Site 1:<br><br>Site 2: (non-recruiting site)	Centre for Clinical Vaccinology and Tropical Medicine (CCVTM), Churchill Hospital,<br>Oxford,<br>OX3 7LE<br>Oxford University Hospitals NHS Foundation Trust,<br>Churchill Hospital<br>Oxford,<br>OX3 7LE
Laboratory Lead	Dr Britta Urban<br><br>Nile Verleur	Oxford Vaccine Group<br>Oxford Vaccine Group
Trial Monitor	Gretchen Meddaugh	Oxford Vaccine Group
Statistician	Xinxue Liu	Oxford Vaccine Group (OVG)

Timepoint	Expected start date	Expected completion date
Recruitment	October 2024	August 2026
Enrolment and Study Visits	October 2024	August 2026
Sample Processing	October 2024	August 2026
Data analysis	October 2024	August 2026

4.1 Laboratory Communication

The lead Researcher and research assistant will be the main point of contact between the clinic and the laboratory and will also be responsible for ensuring study specific supplies and reagents are in stock.

During the study, information of the scheduled visits for each participant (including the day of challenge) will be entered into the adult study planner and the OVG CCVTM Bookings spreadsheet via Microsoft SharePoint. All staff working on the study should ensure they have access to this information and check these documents on a regular basis.

Participant visit information will be entered by the clinical team to the bookings spreadsheet (any discrepancies or queries should be addressed directly with the study lead nurse or doctor). Confirmation or immediate changes of visits will be communicated appropriately by telephone, in person, by email or via MS Teams chat between appointed representatives of the laboratory and clinical team (responsibility will be distributed throughout the laboratory and clinical team).

During the first 10 days after challenge, to ensure the safety of both our participants and staff there will always be a member of the clinical and laboratory team on call (the contact details of core staff will be posted in the laboratory).

Laboratory staffing will be overseen by the OVG laboratory. There is the potential for out of hours and weekend work associated with this study, but this will be minimised where possible (staffing for non-core hours will be managed by the laboratory lead for the study). **Nasal wash samples must be delivered to the CCVTM laboratory within 50 mins of sample collection which must be stringently followed to not compromise the colonisation data for the secondary endpoint. Cut-off time for sample delivery to the lab is 14:30 to allow for enough time for all processing to be completed within the working hours.**

A study specific weekly meeting will be held to discuss all aspects of the ongoing study including safety reporting, recruitment, clinical and laboratory logistics.

5 ANALYTICAL PROCESSES

5.1 Challenge Preparation

Preparation for Spn6b challenge agents must commence once the number of participants is confirmed by the clinic and after they have completed the pre-challenge checks. The confirmation will be communicated in the TEAMS ECLIPSE Coordination chat. All preparation tasks require one operator and one observer to ensure that the relevant Standard Operating Procedures (SOPs) are followed and cross-checked against the observer checklist. The Spn6b challenge inoculum must be administered within 30 minutes of preparation.

All staff involved in the challenge procedures, whether as an operator or observer, must read and understand SOPs OVG2027 and OVGL156 (including form F1). After the challenge, inventory forms (OVGL156-F3) must be completed, including the accountability section.

Additionally, in the file path K:\Adult Studies\2) Adult Set-up\ECLIPSE (Nosevac adult)\3. TMF_Sep 2024\17. Laboratory\17.9 Challenge Agent Documents, the following documents must be updated to reflect the usage of challenge stock:

- Stock Sheet Word document (ensure the physical copy in the microbiology lab in CCVTM CL2 is also updated).
- Stock Spreadsheet for location inventory.
- Scan and upload inventory and observer checklist forms. Original copies should be filed away in the CCVTM CL2 Microbiology room folder.

These updates must be completed for the challenge agent in its respective folders.

5.2 Clinic Collection Sample Processing

Sample processing required to meet the study objectives are listed below and will be performed according to the listed SOP.

Group A: LAIV/Spn

Process	Sample			SOP Ref.	Agreed deviation from SOP or Assay performed by other laboratory
	Container	Volume	Frequency		
Nasal wash	50mL Falcon tube	≥10mL	5 visits	OVGL153<br>RIGRPS005	Liverpool SOP will be developed into OVG version as part of transferring the Experimental Human Pneumococcal Challenge model to OVG.<br>Lab to take pictures of the primary endpoint sample plates for all participants, including negative and positive samples.<br>NWS will be aliquoted in 5 x 2ml tubes<br>NWP will be stored as one aliquot of 800 ul

Process	Sample			SOP Ref.	Agreed deviation from SOP or Assay performed by other laboratory
	**Container**	**Volume**	**Frequency**		
Serum	Red Cap Clot Activator Vacutainer	6mL	4 visits	OVGL175	To spin at 1306 x g for 10mins as per RIGRPS007<br>Aliquot 2 ml into screwcap tubes until no serum remains
PBMC separation	Green Cap Lithium Heparin tube	60mL	4 visits	OVGL175	Follow Liverpool SOP of Leucosep Separation (Figure 4. For overview)
Blood RNA	Red Cap Paxgene Vacutainer	2.5mL	4 visits	OVGL030<br>RIGRPS008	Follow Liverpool SOP
Nasosorption	Blotting paper inside microcentrifuge tube	N/A	7 clinic visits	Liverpool SOP RIGRPS010	Follow Liverpool SOP
Nasal cells FloQ swab	FloQ swabs inside 15mL falcon tubes with medium	N/A	2 visits	OVGL155	FloQswabs: Collect one swab for each nostril at visit D-7 and D28, elute cells from swab and store in CryoABC
Nasal Cells Curette	Curettes inside 15ml falcon tube with medium	N/A	5 visits	OVGL155	Nasal cells (two curettes/nostril, total of four) will be collected on D-7, D2, D7, D9 and D28. Cells will be eluted and stored in cryoABC as one aliquot
Saliva	Orocal sample tube	Variable	7 visits	OVGL183	All aliquots to be frozen at -80°C immediately and eluted as a batch
Bacterial throat swabs	Flocked swab inside tube with 1mL AIMES media	1mL	7 visits	Liverpool SOP RIGRPS010	Bacterial swabs will not be plated but will be frozen at -80C immediately after collection.
Viral throat swabs	Flocked Swab inside tube with UTM	1mL	7 visits	Liverpool SOP RIGRPS010	Follow Liverpool SOP
Fine needle Aspirate (FNA)	15 mL centrifuge tube with 10-12 ml transport media	Variable	1 visit	WHG-SOP-LEGACY001	Process as soon as possible after procedure<br>WHG-SOP-LEGACY001, process at IDRM
Nasal Biopsy	20mL	Variable	1 visit	TBC	Follow procedure appendix 1 until specific SOP submitted and approved

Group B: LAIV only

Process	Sample			SOP Ref.	Agreed deviation from SOP or Assay performed by other laboratory
	Container	Volume	Frequency		
Nasal wash	50mL Falcon tube	≥10mL	4 visits	OVGL153<br>RIGRPS005	Liverpool SOP will be developed into OVG version as part of transferring the Experimental Human Pneumococcal Challenge model to OVG.<br>Lab to take pictures of the primary endpoint sample plates for all participants, including negative and positive samples.<br>NWS will be aliquoted in 5 x 2ml tubes<br>NWP will be stored as one aliquot of 800 ul
Serum	Red Cap Clot Activator Vacutainer	6mL	4 visits	OVGL175	To spin at 1306 x g for 10mins as per RIGRPS007<br>Aliquot 2 ml into screwcap tubes until no serum remains
PBMC separation	Green Cap Lithium Heparin tube	60mL	4 visits	OVGL175	Follow Liverpool SOP of Leucosep Separation (Figure 4. For overview) with agreed deviation
Blood RNA	Red Cap Paxgene Vacutainer	2.5mL	4 visits	OVGL030<br>RIGRPS008 (Appendix A)	Follow Liverpool SOP
Nasosorption	Blotting paper inside microcentrifuge tube	N/A	5 clinic visits	Liverpool SOP RIGRPS010 (Appendix B)	Follow Liverpool SOP
Nasal cells FloQ swab	FloQ swabs inside 15mL falcon tubes with 2 ml medium	N/A	2 visits	OVGL155	FloQswabs: Collect one swab from each nostril (maximum of 2) at D-7 and D31, elute cells from swab and store in CryoABC
Nasal Cells Curette	Curettes inside 15ml falcon tube with medium	N/A	5 visits	OVGL155	Nasal cells (two curettes/nostril, maximum of four) will be collected on D-7 D3, D6 and D31. Cells will be eluted and stored in cryoABC as one aliquot
Saliva	Orocal sample tube	Variable	5 visits	OVGL183	All aliquots to be frozen at -80°C immediately.

Process	Sample			SOP Ref.	Agreed deviation from SOP or Assay performed by other laboratory
	**Container**	**Volume**	**Frequency**		
Bacterial throat swabs	Flocked swab inside tube with 1mL AIMES media	1mL	5 visits	Liverpool SOP RIGRPS010 (Appendix B)	Bacterial swabs will not be plated but will be frozen at -80C immediately after collection.
Viral throat swabs	Flocked Swab inside tube with UTM	1mL	5 visits	Liverpool SOP RIGRPS010 (Appendix B)	Follow Liverpool SOP
Fine needle Aspirate (FNA)	15 mL centrifuge tube with 10-12 ml transport media	Variable	1 visit	TBC	Process as soon as possible after procedure<br>WHG-SOP-LEGACY001, process at IDRM
Nasal Biopsy	20mL	Variable	1 visit	TBC	Follow procedure appendix 1 until specific SOP submitted and approved

5.2.1 Clinic Sample Transport Conditions and Processing Time Window

Group A and Group B:

Sample	Transport Condition	Processing Window
Nasal Wash	Room Temperature	1 hour (Priority) from time of collection
PBMC	Room Temperature	4 hours after collection (on roller)
Serum	Room Temperature	If not processed immediately, can be kept in fridge up to 4 hours after collection
PAXgene	Room Temperature	2.5 hours
Bacterial Throat Swab	Room Temperature	1 hour
Saliva	On Ice	1 hour
Nasosorption (SAM)	On Ice	1 hour
Nasal Cells (Curettes and FloQ swab)	On Ice	1 hour from time of arrival in lab
Viral Throat Swab	On Ice	1 hour
Fine Needle Aspirate (FNA)	Room Temperature	Immediately

Nasal Biopsy	Room Temperature	By end of day

5.3 Home-Collected Samples

Study participants will be trained in home -sampling during their first inoculation visit. These samples will be conducted at the participant place of residence. Samples will be stored at home in biohazard appropriate packaging (supplied by the research team) in their home freezer. A temperature logger will be inside their dedicated transport box that will record and allow for monitoring of the temperature of the samples when at home and during their transfer to clinic. Participants will return the samples on their subsequent clinic visit using cool boxes and frozen ice packs.

Sample	Transport Condition	Processing Window
Saliva	In cool box with -20°C ice packs	1 hour
Nasosorption (SAM)	In cool box with -20°C ice packs	1 hour

Group A and Group B:

**Process**	**Sample**			**SOP Ref.**	**Agreed deviation from SOP**<br>**or**<br>**Assay performed by other laboratory**
	**Container**	**Volume**	**Frequency per participant**		
Nasosorption	Nasal strips	N/A	Group A: 7<br>Group B: 9	N/A	Check labelling, scan into inventory sheet and freeze in -80C ASAP
Saliva	Orocal sample tube	1mL	Group A: 7<br>Group B: 9	N/A	Saliva samples will not be processed immediately. Check labelling, scan into inventory sheet and freeze in -80C ASAP

5.4 Sample Analysis

Assays required to meet the study objectives are listed below and will be performed according to the listed SOP.

**Objective/ endpoint**	**Sample type**	**Storage**	**Assay name**	**SOP Ref.**	**Comments**
Antibody levels	Serum/Plasma	-80°C	ELISA	N/A	To be developed
Antibody levels	Nasal wash	-80°C	ELISA	N/A	To be developed
Antibody levels	Saliva	-80°C	ELISA	N/A	To be developed
Cell mediated immune response	PBMC	LN_2	Flow cytometry	N/A	To be developed
Cell mediated immune response	Nasal cells	LN_2	Flow cytometry	N/A	To be developed
Antibody Secreting Cells	PBMC	LN_2	ELISpot	N/A	To be developed
Transcriptomiocs	Nasal cells	LN_2	single cell RNA sequencing (scRNAseq)	N/A	To be developed
Spatial Transcriptomics	Nasal Biopsy	4°C	Xenium spatial transcriptomics	N/A	To be developed

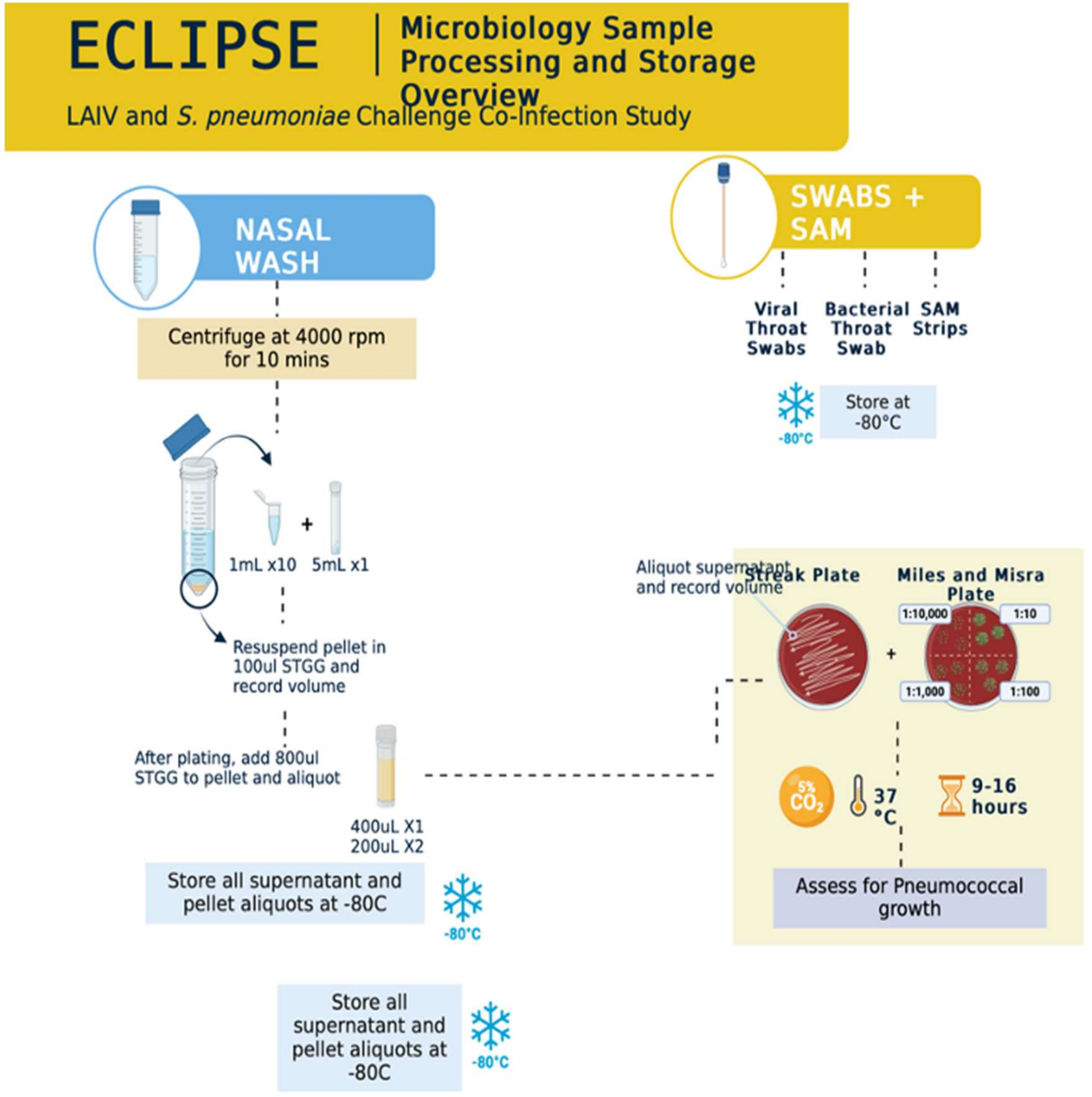
ECLIPSE
Microbiology Sample Processing and Storage Overview
LAIV and S. pneumoniae Challenge Co-Infection Study
NASAL WASH
Centrifuge at 4000 rpm for 10 mins
1mL x10
+
5mL x1
Resuspend pellet in 100ul STGG and record volume
After plating, add 800ul STGG to pellet and aliquot
400uL X1
200uL X2
Store all supernatant and pellet aliquots at -80C
-80°C
Store all supernatant and pellet aliquots at -80C
-80°C
SWABS + SAM
Viral Throat Swabs
Bacterial Throat Swab
SAM Strips
-80°C
Store at -80°C
Aliquot supernatant and record volume
Streak Plate
Miles and Misra Plate
1:10,000
1:10
1:1,000
1:100
+
5% CO2
37 °C
9-16 hours
Assess for Pneumococcal growth
SALIVA
Store all samples at -80°C

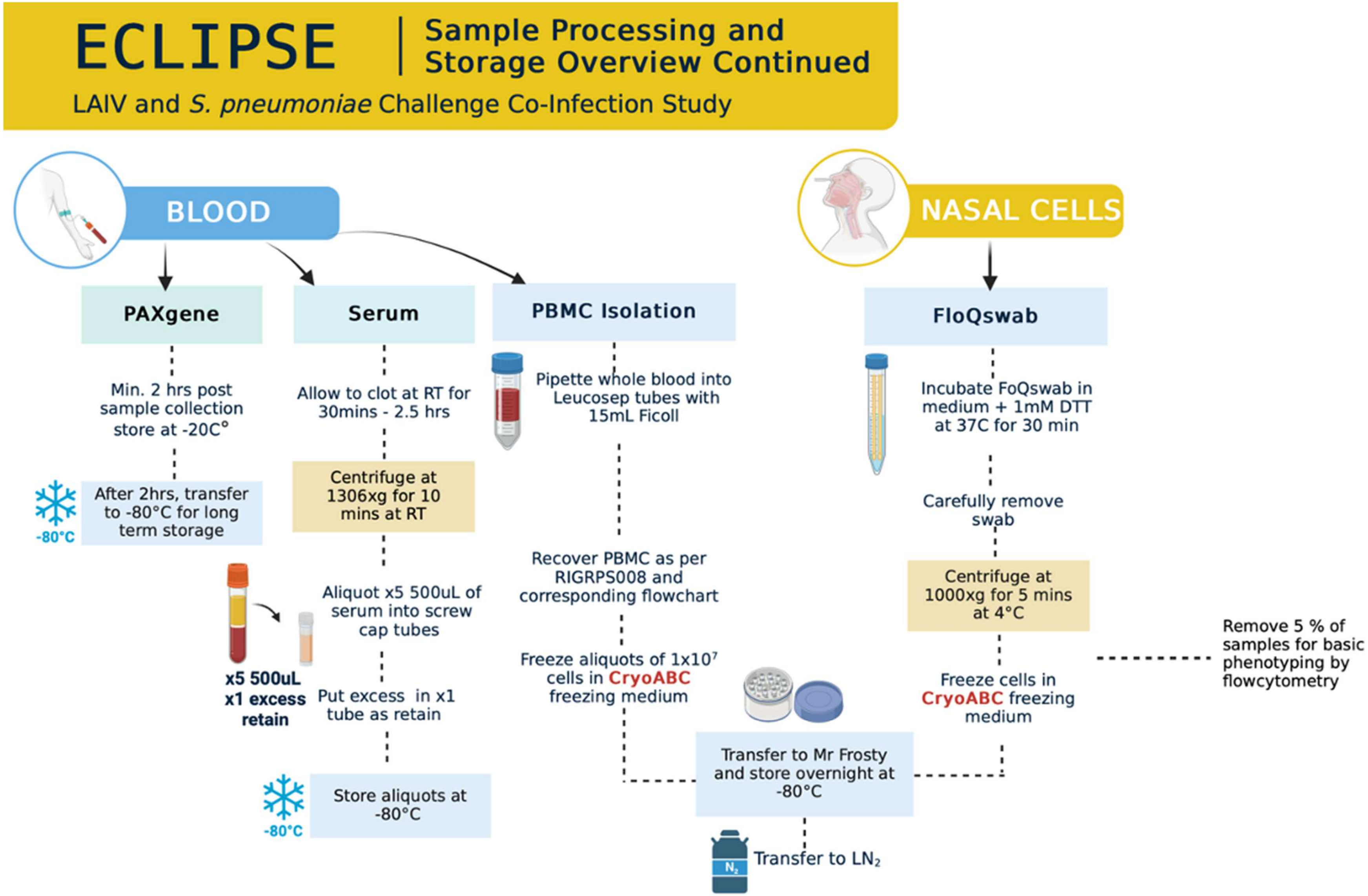
ECLIPSE
Sample Processing and Storage Overview Continued
LAIV and S. pneumoniae Challenge Co-Infection Study
BLOOD
PAXgene
Min. 2 hrs post sample collection store at -20C°
-80°C
After 2hrs, transfer to -80°C for long term storage
Serum
Allow to clot at RT for 30mins - 2.5 hrs
Centrifuge at 1306xg for 10 mins at RT
x5 500uL
x1 excess retain
Aliquot x5 500uL of serum into screw cap tubes
Put excess in x1 tube as retain
-80°C
Store aliquots at -80°C
PBMC Isolation
Pipette whole blood into Leucosep tubes with 15mL Ficoll
Recover PBMC as per RIGRPS008 and corresponding flowchart
Freeze aliquots of 1x10⁷ cells in CryoABC freezing medium
Transfer to Mr Frosty and store overnight at -80°C
Transfer to LN₂
NASAL CELLS
FloQswab
Incubate FoQswab in medium + 1mM DTT at 37C for 30 min
Carefully remove swab
Centrifuge at 1000xg for 5 mins at 4°C
Freeze cells in CryoABC freezing medium
Remove 5 % of samples for basic phenotyping by flowcytometry

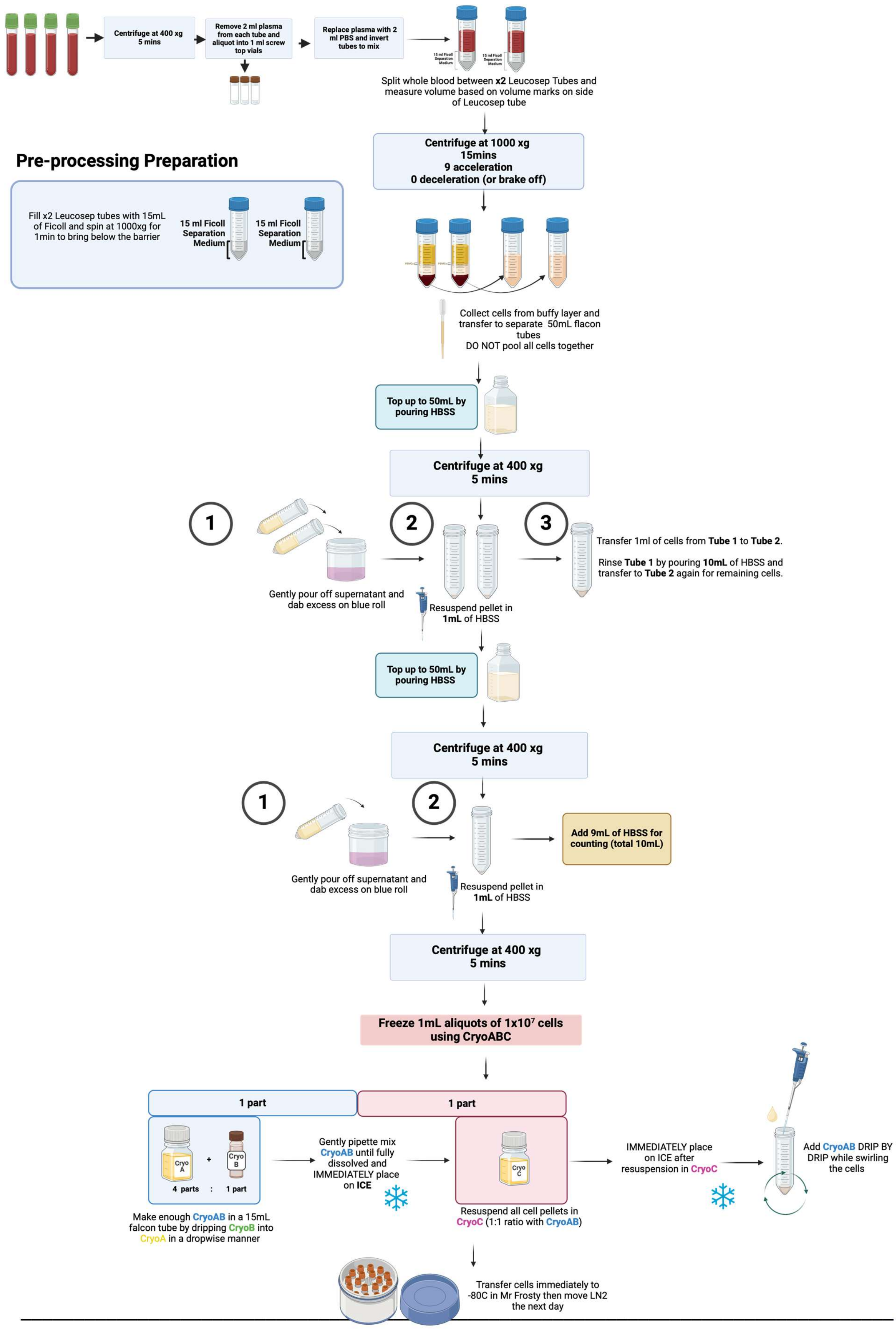
Centrifuge at 400 xg 5 mins
Remove 2 ml plasma from each tube and aliquot into 1 ml screw top vials
Replace plasma with 2 ml PBS and invert tubes to mix
15 ml Ficoll Separation Medium
15 ml Ficoll Separation Medium
Split whole blood between x2 Leucosep Tubes and measure volume based on volume marks on side of Leucosep tube
Centrifuge at 1000 xg
15mins
9 acceleration
0 deceleration (or brake off)
Pre-processing Preparation
Fill x2 Leucosep tubes with 15mL of Ficoll and spin at 1000xg for 1min to bring below the barrier
15 ml Ficoll Separation Medium
15 ml Ficoll Separation Medium
Collect cells from buffy layer and transfer to separate 50mL flacon tubes
DO NOT pool all cells together
Top up to 50mL by pouring HBSS
Centrifuge at 400 xg
5 mins
1
2
3
Gently pour off supernatant and dab excess on blue roll
Resuspend pellet in 1mL of HBSS
Transfer 1ml of cells from Tube 1 to Tube 2.
Rinse Tube 1 by pouring 10mL of HBSS and transfer to Tube 2 again for remaining cells.
Top up to 50mL by pouring HBSS
Centrifuge at 400 xg
5 mins
1
2
Gently pour off supernatant and dab excess on blue roll
Resuspend pellet in 1mL of HBSS
Add 9mL of HBSS for counting (total 10mL)
Centrifuge at 400 xg
5 mins
Freeze 1mL aliquots of 1x10⁷ cells using CryoABC
1 part
1 part
Cryo A
Cryo B
4 parts : 1 part
Make enough CryoAB in a 15mL falcon tube by dripping CryoB into CryoA in a dropwise manner
Gently pipette mix CryoAB until fully dissolved and IMMEDIATELY place on ICE
Cryo C
Resuspend all cell pellets in CryoC (1:1 ratio with CryoAB)
IMMEDIATELY place on ICE after resuspension in CryoC
Add CryoAB DRIP BY DRIP while swirling the cells
Transfer cells immediately to -80C in Mr Frosty then move LN2 the next day

Sample Collection Plan Group A

Timepoints	Challenge	PBMC	Serum	Blood RNA	Nasosorption	Throat swab	Saliva	Nasal Wash	Nasal Cells Swab	Nasal Cells Curette
Sample Tube	N/A	10mL lithium heparin tube	6mL serum tube	2.5mL PAXgene tubes	Nasal strip tube	Viral: 5mL tube + 1mL UTM Bacterial: 5mL tube + 1mL AIMES media	Orocal tube	20mL saline +50mL falcon tube	15mL tubes + 2 ml medium	15mL tubes + 2 ml medium
Sample Volume	N/A	60mL	6mL	2.5mL	N/A	N/A	N/A	≥10mL	N/A	N/A
Screen										
D-7		x$^{\gamma}$	x$^{\gamma}$	x$^{\gamma}$	x	x	x	x	x	x
D-3	x									
D 0	x	x	x	x	x	x	x			
D 2					x	x	x	x		x
D 3	x				x	x	x			
D 7		x	x	x	x	x	x	x		x
D 9					x	x	x	x		x
D28		x	x	x	x	x	x	x	x	x

FNA will be collected and processed immediately at the IDRM

Y Additional safety bloods can be taken if screening visit was more than 21 days prior to baseline visit

Sample Collection Plan Group B

Timepoints	LAIV Challenge	PBMC	Serum	Blood RNA	Nasosorption	Throat swab	Saliva	Nasal Wash	Nasal Cells Swab	Nasal Cells Curette
Sample Tube	N/A	10mL lithium heparin tube	6mL serum tube	2.5mL PAXgene tubes	Nasal strip tube	Viral: 5mL tube + 1mL UTM Bacterial: 5mL tube + 1mL AIMES media	Orocal tube	20mL saline +50mL falcon tube	15mL tubes + 2 ml medium	15mL tubes + 2 ml medium
Sample Volume	N/A	60mL	6mL	2.5mL	N/A	N/A	N/A	≥10mL	N/A	N/A
Screen										
Baseline/D-7		x[Y]	x[Y]	x[Y]	x	x	x	x	x	x
D 0	x				x	x	x			
D 3			x	x	x	x	x	x		x
D 6		x	x	x	x	x	x	x		x
D31		x	x	x	x	x	x	x	x	x

FNA will be collected and processed immediately at the IDRM

Y Additional safety bloods can be taken if screening visit was more than 21 days prior to baseline visit

Home sampling Sample Collection Chart-Group A

Timepoints	Nasosorption	Saliva
Sample Tube	Nasal strip tube	Orocal tube
Sample Volume	N/A	N/A
D-2	x	x
D-1	x	x
D 1	x	x
D 4	x	x
D 5	x	x
D 6	x	x
D14	x	x

Home-sampling Sample Collection Chart-Group B

Timepoints	Nasosorption	Saliva
Sample Tube	Nasal strip tube	Orocal tube
Sample Volume	N/A	N/A
D 1	x	x
D 2	x	x
D 4	x	x
D 5	x	x
D 7	x	x
D 8	x	x
D 9	x	x
D 10	x	x
D 17	x	x

5.5 Equipment and Reagents

Equipment and Reagents required to meet the study objectives are listed below. Please refer to individual SOPs for detailed information.

Assay	Equipment required	Consumables required	Reagents required
Culturing Streptococcus pneumoniae as part of processing nasal wash and bacterial swab	Centrifuge, Class II Microbiological safety cabinet, CO_2 incubator, -80°C freezer	Labels, Tubes, cryoboxes, Pipettes & Pipette Tips, Columbia Horse Blood agar plates, STGG media, THY media, Gentamycin, L-Spreaders, inoculation loops, saline, 96-well plates, 50mL falcon tubes,	STGG medium: skimmed-milk-tryptone-glucose 10% glycerol<br>THY medium: Todd Hewitt Yeast broth 10% glycerol<br>Gentamycin 1mg/mL
Serum separation and storage	Centrifuge, Class II Microbiological safety cabinet, freezer (-80°C)	Labels, cryovials, cryoboxes, pipettes, and tips	-
PBMC Isolation and freezing	Centrifuge, Class II biological safety cabinet, freezer (-80°C), cell counter, liquid nitrogen cryostore	Pipettes and tips, labels, cryovials, falcon tubes, Leucosep tubes tubes, Mr Frosty, cryoboxes and inserts	Ficoll, HBSS, Cryo-ABC, Red cell lysis buffer, Trypan blue 0.4%
Acquisition of samples by flow cytometry	Centrifuge, Class II Microbiological safety cabinet, freezer(-80°C), Aurora cytometer	Pipettes and tips, labels, cryovials, falcon tubes, Serological pipettes, 70um filters, FACS tubes, timer	Antibodies, PBS, FBS (heat inactivated), EDTA, viability reagents
ELISPOT	ELISpot reader, Centrifuge, Class II Microbiological safety cabinet, freezer (-80°C and -20°C)	Tissue culture plate, multi-channel pipettes, pipette tips, steripette, serological pipettes, vortex, eppendorf tubes, reagent reservoirs,	Antigens, RPMI cell culture medium, fetal bovine serum, Penicillin/ Neomycin/ Streptomycin antibiotic mix, PBS, BCIP/NBT substrate, sterile water, ethanol, DNAse,
ELISA	Vortex, ELISA Plate Reader and Plate Washer	96 well ELISA plate, multi-channel pipettes, pipette tips, plate sealer, Vortex, Eppendorf tubes, reagent reservoirs	Coating antigens, wash buffers, block buffers, secondary antibody, substrate, stopping solution, PBS-Tween, carbonate-bicarbonate

5.5.1 Non-commercial reagent requirements

Streptococcus pneumoniae serotype 6B (Spn6B) BHN418 strain, manufactured and supplied by Liverpool School of Tropical Medicine. Fluenz, produced by AstraZeneca UK Ltd.

5.6 Sample Shipment and Long-Term Storage

Shipping & storage of study samples will be conducted according to OVGL018 & OVGL003.

5.6.1 Local Sample storage

Group A

Assay/Sample	Storage	Number of Subjects	Number of Visits	Aliquots per sample	Total	Samples per box	Number of boxes
PBMC	LN2	40	4	6	960	81	12
Serum	-80°C Freezer	40	4	8	1280	81	16
Blood RNA	-80°C Freezer	40	4	1	160	81	2
Fine needle Aspirant (FNA)	LN2	TBC	1	1	TBC max 40	81	1
Saliva	-80°C Freezer	40	14	4	2240	81	28
Bacterial throat swab	-80°C Freezer	40	7	1	280	81	4
Viral throat swab	-80°C Freezer	40	7	1	280	81	4
Nasosorption (SAM)	-80°C Freezer	40	14	2	2240	81	28
Nasal wash	-80°C Freezer	40	4	14	2240	81	28
Nasal cells	LN2	40	5	2	400	81	5
Nasal Biopsy	4°C	5	1	1	5	5	1

Group B

Assay/Sample	Storage	Number of Subjects	Number of Visits	Aliquots per sample	Total	Samples per box	Number of boxes
PBMC	LN2	20	4	6	480	81	6
Serum	-80°C Freezer	20	4	8	640	81	8
Blood RNA	-80°C Freezer	20	4	1	80	81	1
Fine needle Aspirant (FNA)	LN2	20	1	1	TBC max 40	81	1
Saliva	-80°C Freezer	20	14	4	560	81	7
Bacterial throat swab	-80°C Freezer	20	5	1	100	81	2
Viral throat swab	-80°C Freezer	20	5	1	100	81	2
Nasosorption (SAM)	-80°C Freezer	20	14	2	1120	81	14
Nasal wash	-80°C Freezer	20	4	14	2240	81	28
Nasal cells	LN2	20	4	2	200	81	2
Nasal Biopsy	4°C	5	1	1	5	5	1

Freezer and Cryostore space requirements:

Storage Space	Number of boxes
-80°C	163
LN_2	30

5.6.2 Sample Shipment and External Storage

Assay / Sample	Post study storage	Expected shipment / storage date
All of above samples	The study is part of a larger EU-funded consortium NoseVac. Samples may be shipped to our collaborator(s) for analysis<br>Samples with consent will be transferred to Biobank after the end of the study for long term storage and future use. Date and details to be confirmed.	Samples can be shipped at any timepoint based on mutual agreement and with the appropriate MTA in place.<br>Rest of the samples

5.6.3 Disposal of samples

In the event that study participants withdraw their consent or a sample integrity has been compromised, the sample(s) will have to be disposed of using appropriate procedures (autoclaving) and the disposal will be logged. If research samples are collected during a combined screening and baseline visit, the research samples for a given study participant will be destroyed at the end of the season (spring 2025 or spring 2026) if that study participant fails the eligibility criteria.

6 DATA MANAGEMENT

All laboratory information relating to the study will be recorded as listed below to ensure all analyses are complete and performed according to the clinical protocol. Data will be recorded, analysed and stored/archived according to the following central OVC SOPs, plus others for local procedures on sample storage, tracking & result reporting, e.g.;

OVG019 Data Management of OVG Clinical Trials and Research Studies

OVC020 Archiving

OVGL030 Sample Handling, Receipt, Tracking, Processing & Storage

OVGL033 ELISpot Sample Tracking

OVC037 Confidentiality and Information Security

Data for the individual assays is captured & processed electronically using equipment as described in Table below. Final Data files for all assays will be stored in the assay specific folder in the Laboratory section of the study specific electronic TMF. These assay folders will be created at the start of the study for easy saving and tracking of data. Where the final data analysis file is too large to be stored in the TMF, the actual location must be signposted in the TMF.

Assay	Data capture & primary data format	Primary Storage Location	Primary Data QC Check	Data Export for analysis	QC check of exported data	Data processing	QC Check of data processing	Final Data Analysis
ELISA (OVG)	ELISA plate reader and software. Data exported to MS Excel report when plate is read and saved onto study specific K: Drive.	MS Excel report saved to study specific folder on the S: Drive	QC pass/fail criteria are pre-defined in assay programme, and the MS Excel report confirms pass/fail status of test samples.	Data from report entered into master spreadsheet<br>MS Excel<br>Saved in study specific folder on the S: Drive	MS Excel sheet will be opened to check that the data has been exported from the experiment file. It will be saved as plate number_date_operator initial. The experiment files will be saved in the same folder of the raw data can always be exported again at a later date.	MS Excel<br>See OVGL026	The data will be checked for duplications and to ensure all the participant results are included	GraphPad Prism / R as appropriate for complexity of data
ELISpot<br>(OVG)	AID Plate Reader<br>AID Plate file	Study specific folder on x drive (AID computer cannot be networked)<br>Transferred to study specific folder on S: Drive	Plate count checked manually for artefacts / poor quality read.	Data auto exported from AID software into single Excel file (Data Accumulate)<br>Saved in study specific folder on S: Drive	Correct plates have all been exported.	MS Excel<br>See OVGL033 & OVGL015	N/A	GraphPad Prism / R as appropriate for complexity of data

Assay	Data capture & primary data format	Primary Storage Location	Primary Data QC Check	Data Export for analysis	QC check of exported data	Data processing	QC Check of data processing	Final Data Analysis
Flow cytometry	Sony or Aurora spectral flow cytometer; fcs files	Fcs files are transferred to study specific folder on D: Drive	Within specific software such as FlowJo or Omiq	Data exported from software into Excel file. Saved in study specific folder on S: Drive	Data were QCed before export using specific analysis software (FlowJo, Aurora)	N/A	N/A	GraphPad Prism / R as appropriate for complexity of data

7 QUALITY CONTROL AND QUALITY ASSURANCE

The laboratory results will provide a large proportion of the data collected and analysed in this study. Measures taken to ensure the quality of the data are listed below.

The SOP for each assay includes details of quality control samples and processes. QC failures will be reviewed according to SOP OVC027 Management of Complaints, Errors, Deviations and Non-Conformance.

Sample receipt, storage and shipment will be reviewed according to the study monitoring plan. If additional quality assurance is required, this will be included in the monitoring plan or as part of an internal audit, conducted by the QA manager.

8 REFERENCES

Good Clinical Laboratory Practice (GCLP) A Quality System for Laboratories that undertake the Analysis of Samples from Clinical Trials. BARQA. (**ISBN** 978-1-904610-00-7)

Guidance on the maintenance of regulatory compliance in laboratories that perform the analysis or evaluation of clinical trial samples. MHRA. July 2009.

9 APPENDICES

Appendix A:

9.1 Nasal Biopsy processing – Protocol

Key reagents and consumables:

Cat#	Item
FOR0020AF59001	Formalin 10%, Q Path®, buffered in collection pot

Fixation Protocol:

1. Once collected – using sterile forceps gently transfer the nasal biopsy immediately to the room temperature 10% neutral buffered formalin within the collection jar
   1.1. Ensure the time of collection is noted – the sample **should not** remain in room temperature formalin for longer than 24 hours to preserve RNA quality and epitope retrieval
2. Transfer the sample to the histology service of choice at RT on the day of collection where they will be paraffin embedded within 24 hours → ensure orientation instructions below are included
   2.1. In case of emergency – if samples cannot be embedded within 24 hours of fixation, then transfer the biopsy to a container with 70% ethanol and ensure embedding occurs within 48 hours.
   2.2. Minimise physical disruption/motion during transfer

   *Note – delaying embedding will reduce success of subsequent epitope retrieval for IHC and spatial transcriptomics

Histopathology Processing Request for Nasopharyngeal Biopsies (FFPE for Spatial Transcriptomics)

Purpose: To ensure optimal embedding and sectioning of nasal biopsy specimens for downstream **spatial transcriptomic analysis**, with preserved architecture from **epithelial surface through basal layers**, and **high RNA and antigen integrity**.

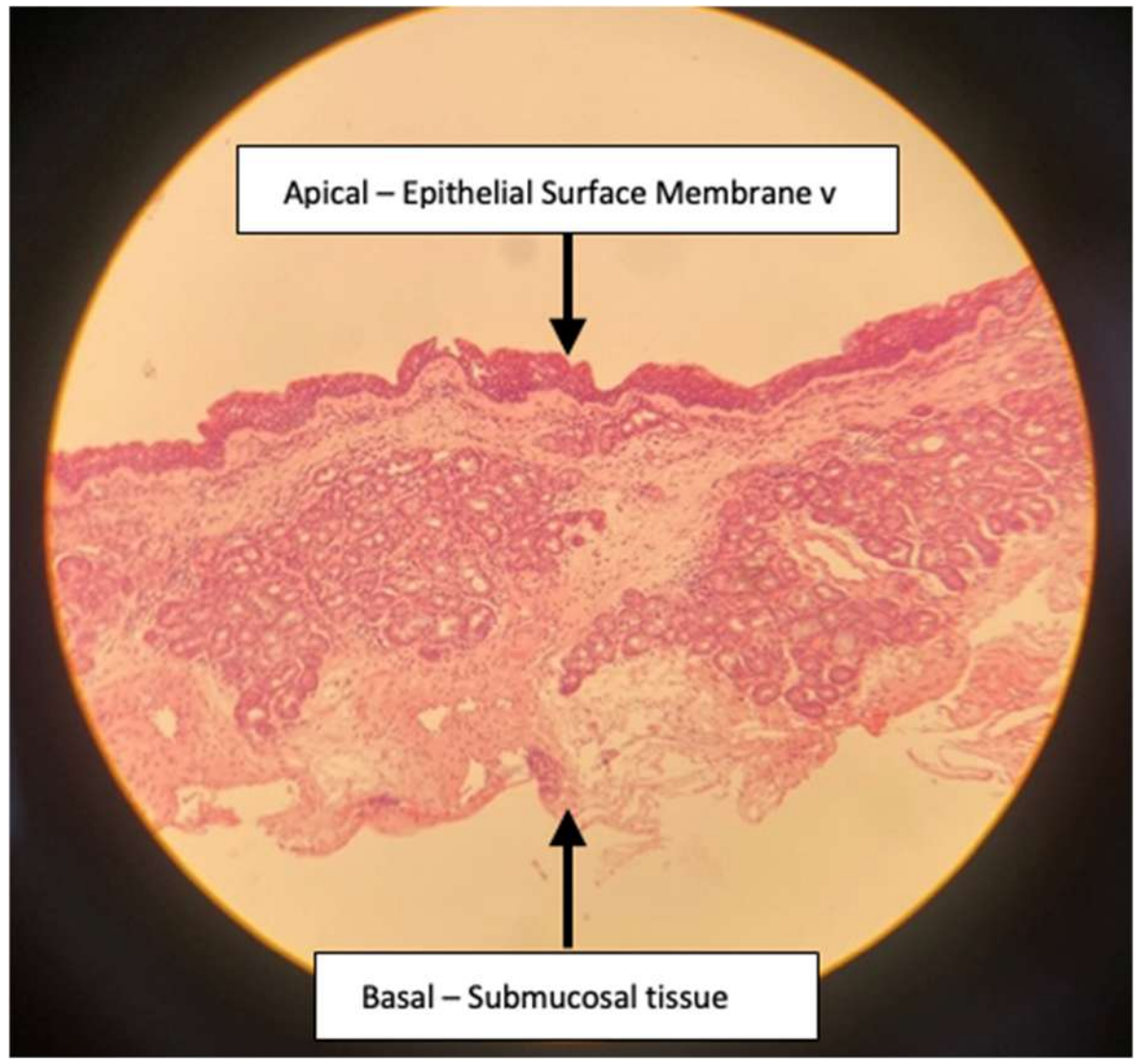

1. Specimen Type and Orientation

- **Specimens**: Small nasal mucosal biopsies ~2–4mm in diameter.
- **Orientation critical**: The surface epithelium must be **oriented upward** in the paraffin block, to enable transverse sectioning **from apical surface to underlying lamina propria** – we want to visualise the layers of the nasal mucosa and underlying tertiary lymphoid organs.

2. Fixation Protocol

- Once collected, samples are immediately transferred to room temperature **10% neutral buffered formalin**. Refer to the documented collection and fixation times.
- Ensure fixation does not exceed **24 hours** to preserve RNA quality and epitope retrieval.

3. Sectioning Request -

- Trim initial block surfaces gently until epithelial surface appears.
- Cut **one 4–5 µm section** for H&E stain for QC purposes

4. Slide Handling

- Label each slide with:
  - Sample ID
  - Orientation (mark apical edge if possible)
  - Date of sectioning

5. Documentation & Communication

- Please note:
  - Orientation at embedding
  - Time in formalin
  - Block and slide IDs
- Immediately alert the research team if:
  - Orientation is ambiguous
  - Sample is degraded or fragmented
  - Fixation appears suboptima

Contact for Questions

Please consult the Oxford Vaccine Group ECLIPSE study team lead before deviating from the protocol, especially in cases of:

- Ambiguous biopsy orientation
- Unusual tissue morphology
- Questions regarding slide preparation for spatial platforms

Primary contacts:

1. Nile Verleur
2. Britta Urban
