# Supplementary material for: Establishing a human co-infection challenge study of Streptococcus pneumoniae (Spn) and live attenuated influenza vaccine (LAIV) in older healthy adults: Exploring Co-infection with Live Attenuated Influenza Vaccine and PneumococcuS in healthy oldEr adults (ECLIPSE), a UK-based single-centre, single-blind randomised controlled trial
Source: BMJ Open. 2026 Oct 12;16(10):e118632. doi: 10.1136/bmjopen-2026-118632 (PMC13662028; doi:10.1136/bmjopen-2026-118632)
Supplement: Supplementary file 3 [file bmjopen-16-10-s005.pdf]

Appendix 3: Study Objectives

Objectives	Outcome Measures
**Primary Objective:**<br><br>1. To determine if a co-infection human challenge model with LAIV and *S. pneumoniae* (Spn6B) is feasible and safe.	1. Absence of SAEs/AESIs relating to inoculation throughout the study.
**Secondary Objectives**<br><br>1. If co-infection with LAIV and Spn6B alter upper and lower respiratory tract infection symptoms<br>2. How primary LAIV vaccination alters the:<br>(a) Risk of secondary Spn6B carriage<br>(b) Density of secondary Spn6B carriage<br>(c) Duration of secondary Spn6B carriage<br>3. How primary Spn6B challenge or carriage alters the:<br>(a) Risk of secondary LAIV infection (ie. detection of LAIV)<br>(b) Viral load of secondary LAIV infection (density over time)<br>4. If nasal colonisation with other respiratory viruses and bacteria is detectable.<br>5. To evaluate changes in nasal cells phenotype and function over time.	1. Symptomatology using diary cards, with clinical review of LRTI and URTI symptoms at visits.<br>2. Classical culture and molecular methods including RT-qPCR<br>(a) Colonisation rates of Spn6B carriage<br>(b) Density of Spn6B carriage<br>(c) Longitudinal quantification<br>3. Secondary LAIV infection and viral load determined by RT-qPCR.<br>4. Detection of most common respiratory viruses and other respiratory bacteria will be assessed by microfluidic qPCR.<br>5. Analysis of changes in cell populations including antigen-specific T and B cells at different time points in nasal mucosa.
**Exploratory Objectives**<br><br>1. To identify if primary Spn6B challenge or carriage alters immune responses to secondary LAIV vaccination (innate and adaptive) and vice-versa.<br>2. To determine T cell receptor and B cell receptor sequence at the single cell level in nasal cells.<br>3. To evaluate changes in inflammatory markers and antibody responses to pneumococcus and LAIV in the mucosa.<br>4. To compare selected immune parameters both at the cellular and transcriptomic level between the nasal mucosa and systemic circulation.<br>5. Comparison of immune response to LAIV vaccination over the life course (children, younger adults, older adults).<br>6. Comparison of immune and transcriptional parameters of	1. Assessment of immune responses including nasal cytokines, cell populations, Influenza- and Spn6B-specific antibodies and cellular immunity before and after each pathogen challenge.<br>2. T cell receptor and B cell receptor sequencing in parallel with scRNAseq to determine clonotypes in the nasal mucosa in response to LAIV/Spn6B co-infection.<br>3. Analysis of changes in antibody levels and inflammatory markers at different time points in nasal lining fluid, saliva and/or throat swabs.<br>4. Analysis of antibody level, inflammatory markers and cell populations (cellular and transcriptome level) in systemic circulation and compare data with those generated at the nasal mucosa.

experimental co-infection with Spn6B and LAIV with data obtained from natural infected older adults (symptomatic and asymptomatic).<br>7. To determine the frequency, phenotype, and function of immune cells in axillary secondary lymphoid tissue compared to blood and nasal mucosa following intranasal immunisation, in both older and younger adults.<br>8. To characterise transcriptional changes in immune cells (single cell and bulk RNA sequencing) in response to LAIV and Spn6B challenge/carriage/vaccination.	5. Meta analysis of data generated in this study with comparable data generated in a parallel study on mucosal immune responses to infection with LAIV in children.<br>6. Meta analysis of data generated in this study with comparable data generated by collaborators in a hospital study (University of Geneva).<br>7. Single cell ribonucleic acid sequencing (scRNA-seq) to measure cell by cell transcriptomes in lymph node cells and/or multiparameter flow cytometry.<br>8. ScRNAseq and bulk transcriptome analysis to compare gene expression in response to Spn6B and LAIV co-infection or LAIV infection alone
