# Supplementary material for: Establishing a human co-infection challenge study of Streptococcus pneumoniae (Spn) and live attenuated influenza vaccine (LAIV) in older healthy adults: Exploring Co-infection with Live Attenuated Influenza Vaccine and PneumococcuS in healthy oldEr adults (ECLIPSE), a UK-based single-centre, single-blind randomised controlled trial
Source: BMJ Open. 2026 Oct 12;16(10):e118632. doi: 10.1136/bmjopen-2026-118632 (PMC13662028; doi:10.1136/bmjopen-2026-118632)
Supplement: Supplementary file 5 [file bmjopen-16-10-s007.pdf]

Study Title: Human co-infection challenge study of S. pneumoniae (Spn) and Live Attenuated Influenza Vaccine (LAIV) in older healthy adults

Internal Reference Number: OVG2023/07

Short title: Exploring **C**o-infection with **L**ive Attenuated **I**nfluenza Vaccine and **P**neumococcu**S** in healthy old**E**r adults **(ECLIPSE)**

Ethics Ref: 24/EM/0200

IRAS Project ID: 343692

Date and Version No: 20 /Jan /2026, V4.2

**Chief Investigator:**	Professor Katrina Pollock
**Lead Scientific Investigator:**	Professor Daniela Ferreira
**Sponsor:**	University of Oxford
**Funder:**	European Commission - Horizon Programme
**Chief Investigator Signature:**	Katrina Pollock, Oxford Vaccine Group, University of Oxford

Statistician Signature: Xinxue Liu, Oxford Vaccine Group, University of Oxford

Confidentiality Statement

This document contains confidential information that must not be disclosed to anyone other than the Sponsor, the Investigator Team, HRA, host organisation, and members of the Research Ethics Committee, unless authorised to do so.

Study Title: Human co-infection challenge study of S. pneumonia (Spn) and Live Attenuated Influenza (LAIV) in older healthy adults
Protocol Date and Version No: 20/Jan /2026, V4.2

Protocol signature page

The undersigned has read and understood the study protocol detailed above and agrees to conduct the study in compliance with the protocol.

Katrina Pollock	[signature]	Oxford Vaccine Group	26 January 2026
**Principal Investigator** (Please print name)	**Signature**	**Site name or ID number**	**Date**

CONTENTS

1. KEY CONTACTS 6

2. LAY SUMMARY 7

3. SYNOPSIS 8

4. ABBREVIATIONS 9

5. BACKGROUND AND RATIONALE 10

Pneumococcal Disease and Impact 10

The Experimental Human Pneumococcal Colonisation (EHPC) model 11

EHPC in older adults 11

EHPC of Influenza and pneumococcal co-infection in younger adults 12

Immune Response and Age 13

Study Rationale 13

6. OBJECTIVES AND OUTCOME MEASURES 14

7. STUDY DESIGN 15

8. PARTICIPANT IDENTIFICATION 18

8.1 Study Participants 18

8.2 Inclusion Criteria (Group A and B) 18

8.3 Exclusion Criteria 19

9. PROTOCOL PROCEDURES 21

9.1 Recruitment 21

9.2 Screening and Eligibility Assessment 25

9.3 Informed Consent 26

9.4 Randomisation 26

9.5 Blinding and code-breaking 27

9.6 Visit Summary 28

9.6.1 Baseline assessments 28

9.6.2 Schedule of Events – Group A 29

9.6.3 Schedule of Events - Group B 30

9.6.4 Blood Volumes 32

9.7 Description of study intervention, study procedures (clinical) and concomitant medications 32

9.7.1 Description of Study Interventions 32

9.7.2 Study Procedures 36

9.7.3 Concomitant medications 39

9.7.4 Post Study Vaccinations 39

9.8 Sample handling ... 40
9.8.1. Clinical laboratory samples ... 40
9.8.2. Immunology Research samples ... 41
9.8.3. Laboratory Processes ... 41
9.9 Definition of Enrolment ... 43
9.10 Early Discontinuation/Withdrawal of Participants ... 43
9.11 Definition of End of Study ... 44
10. SAFETY REPORTING ... 44
10.1 Definitions ... 44
10.2 Grading ... 45
10.3 Causality ... 45
10.4 Procedure for collecting and recording of adverse events ... 46
10.4.1 E-diary AEs ... 47
10.4.2 Unsolicited adverse events ... 47
10.4.3 Vital sign AEs ... 47
10.4.4 Laboratory AEs ... 48
10.6 Adverse Events of Special Interest (AESI) ... 48
11. STATISTICS AND ANALYSIS ... 49
11.1 Statistical Analysis Plan (SAP) ... 49
11.2 Description of the Statistical Methods ... 49
11.3 Sample Size Determination ... 49
11.4 Populations for analysis of primary and secondary endpoints ... 50
11.5 Decision points ... 50
11.6 Stopping rules ... 50
11.7 The Level of Statistical Significance ... 51
11.8 Procedure for Accounting for Missing, Unused, and Spurious Data. ... 51
11.9 Procedures for Reporting any Deviation(s) from the Original Statistical Plan ... 51
12. DATA MANAGEMENT ... 51
12.1 Source Data ... 52
12.2 Access to Data ... 52
12.3 Data Recording and Record Keeping ... 52
13. QUALITY ASSURANCE PROCEDURES ... 53
13.1 Risk assessment ... 53
13.1.1. Risks related to FNA of lymph nodes ... 54

13.1.2. Risks related to Live Attenuated Influenza Vaccine 55
13.1.3. Risks related to Pneumococcal inoculation 55
13.2 Study monitoring 56
13.3 Study Committees 56
14. PROTOCOL DEVIATIONS 57
15. SERIOUS BREACHES 57
16. ETHICAL AND REGULATORY CONSIDERATIONS 57
16.1 Declaration of Helsinki 57
16.2 Guideline for Good Clinical Practice 57
16.3 Approvals 57
16.4 Other Ethical Considerations 57
16.5 Reporting 58
16.6 Transparency in Research 58
16.7 Participant Confidentiality 58
16.8 The Over-volunteering Prevention System 58
16.9 Expenses and Benefits 58
17. FINANCE AND INSURANCE 59
17.1 Funding 59
17.2 Insurance 59
17.3 Contractual arrangements 59
18. PUBLICATION POLICY 59
19. DEVELOPMENT OF A NEW PRODUCT/ PROCESS OR THE GENERATION OF INTELLECTUAL PROPERTY 60
20. ARCHIVING 60
21. REFERENCES 60
22. APPENDIX A: Upper and lower respiratory symptom score 64
23. APPENDIX B: Grading the severity of visit-observed Adverse Events 65
24. APPENDIX C: Grading the severity of laboratory Adverse Events 65
25. APPENDIX D: Amendment History 65

1. KEY CONTACTS

**Chief Investigator**	**Professor Katrina Pollock**<br>Oxford Vaccine Group (OVG)<br>University of Oxford<br>Centre for Clinical Vaccinology & Tropical Medicine (CCVTM)<br>Churchill Hospital, Oxford OX3 7LE
**Lead Scientific Investigator**	**Professor Daniela Ferreira**<br>Oxford Vaccine Group,<br>University of Oxford,<br>Centre for Clinical Vaccinology and Tropical Medicine (CCVTM),<br>Churchill Hospital, Oxford, OX3 7LE,<br>United Kingdom<br><br>Liverpool Vaccine Group, Liverpool School of Tropical Medicine,<br>Accelerator Building, 1 Daulby Street, Liverpool, L7 8XZ
**Scientific Investigators**	**Professor Arnaud Didierlaurent**<br>Centre of Vaccinology and Translational Research in Immunology<br>Department of Pathology and Immunology<br>Faculty of Medicine<br>Rue Michel-Servet 1, CH-1211 Geneve<br>University of Geneva<br><br>**Dr Britta Urban**<br>Oxford Vaccine Group,<br>University of Oxford,<br>Centre for Clinical Vaccinology and Tropical Medicine (CCVTM),<br>Churchill Hospital, Oxford, OX3 7LE,<br>United Kingdom<br><br>**Dr Carla Solorzano-Gonzalez**<br>Oxford Vaccine Group,<br>University of Oxford,<br>Centre for Clinical Vaccinology and Tropical Medicine (CCVTM),<br>Churchill Hospital, Oxford,<br>OX3 7LE,<br>United Kingdom
**Sponsor**	**University of Oxford**<br>Research Governance, Ethics & Assurance Team (RGEA)<br>Boundary Brook House, Churchill Drive, Headington,<br>Oxford, OX3 7GB
**Funder(s)**	European Union (grant number: 101080528-NOSEVAC) and Horizon Europe Guarantee Extension. UKRI Reference number: 10077113

	UK Research and Innovation<br>Polaris House<br>Swindon<br>SN2 1FL
**Clinical Trials Unit**	Primary Care and Vaccine Collaborative Clinical Trials Unit,<br>Oxford Vaccine Group,<br>Centre for Clinical Vaccinology and Tropical Medicine (CCVTM),<br>Churchill Hospital Old Road,<br>Headington<br>Oxford<br>United Kingdom<br>OX3 7LE
**Study Sites**	**Site 1:** Centre for Clinical Vaccinology and Tropical Medicine (CCVTM),<br>Churchill Hospital,<br>Oxford,<br>OX3 7LE<br><br>**Site 2:** (**non-recruiting site)**<br>Oxford University Hospitals NHS Foundation Trust,<br>Churchill Hospital<br>Oxford,<br>OX3 7LE
**Study Monitor**	Oxford Vaccine Group (OVG) Internal Quality Assurance team<br>Oxford Vaccine Group<br>Centre for Clinical Vaccinology and Tropical Medicine (CCVTM)<br>Churchill Hospital, Oxford, OX3 7LE
**Statistician**	Xinxue Liu<br>Oxford Vaccine Group (OVG)<br>University of Oxford<br>Centre for Clinical Vaccinology and Tropical Medicine (CCVTM)<br>Churchill Hospital, Oxford, OX3 7LE
**DSMC Chair**	Professor Adam Dale<br><br>University Hospital Southampton<br><br>Tremona Road, Southampton SO16 6YD

2. LAY SUMMARY

In the study, we hope to understand the way the body reacts to a combination of the flu (influenza) virus and *Streptococcus pneumoniae* (Spn, pneumococcus) *bacteria*. We will look at combining the two germs to see if it will help us understand respiratory infections, and the immune system cells in the nose. Pneumococcal bacteria can cause chest infections such as pneumonia, more often in young children and

older adults. The information gained by doing the study could help us develop new treatments and vaccines for the influenza virus and pneumonia.

This co-infection model has been well tested in younger adults, and we want to compare these results with a group of older adults. The primary goal of this study is to understand if this would be safe and beneficial to study in this age group.

Safety is important in this study. We would be using an approved nasal vaccine for the flu called the Live Attenuated Influenza Vaccine (LAIV) to mimic the way the immune system responds to the virus. We have chosen 'pneumococcal serotype 6B' for the pneumonia germ, as it has been shown to be safe and well tolerated in older adults. Pneumococcal serotype 6B mostly affects the upper airways and can be easily treated with antibiotics to take in the event of a chest infection. There would be a study doctor available to call to discuss any concerns related to the study throughout their journey in the study.

We want to study two groups, A and B. Participants who take part in the co-infection study Group A will be adults between the ages of 55 and 80. They will receive the pneumococcal bacteria on Day 0, and LAIV either on Day –3 (before the pneumococcal bacteria) or Day 3 (after the pneumococcal bacteria).

Participants in both groups will either get saltwater or the attenuated flu virus, and they will not know which one they received. We call this blinding. However, the study staff will not be blinded and will know what each participant has had to ensure participant safety.

Group B will receive the LAIV only on Day 0. This group will be open to older (55-80 years) and younger (18-49 years) adults. They will not receive the pneumococcal bacteria.

Throughout the study we will take samples from the nose, throat, saliva and blood to look at how the immune system responds and keep a diary to follow immune cells and markers. Participants will collect saliva and nasal samples at home.

3. SYNOPSIS

Study Title	Human co-Infection challenge study of S. pneumoniae (Spn) and Live Attenuated Influenza Vaccine (LAIV) in older healthy adults
Internal ref. no. / short title	OVG2023/07<br><br>Exploring Co-infection with Live attenuated Influenza vaccine and PneumococcuS in oldEr adults (ECLIPSE)
Study registration	ISRCTN: 13284643<br>Date registered: 15/10/2024
Sponsor	University of Oxford
Funder	European Union (grant number: 101080528-NOSEVAC) and Horizon Europe Guarantee Extension. UKRI Reference number: 10077113
Study Design	This study involves 2 groups. Group A: single-blind controlled, outpatient, ambulatory human co-infection study. A comparator Group B: unblinded outpatient ambulatory study of vaccine response.
Study Participants	60 healthy older adults (55-80 years), with a control subset of up to 10 younger adults (18-49 years) and 10 older adults (55-80 years)
Sample Size	Up to 60 participants randomised and up to 20 control participants
Planned Study Period	1st September 2024 – 31st January 2028

Planned Recruitment period	01/Sep/2024 to 31 /Mar/2026
Objectives	
Primary	To determine if a co-infection human challenge model with LAIV and *S. pneumoniae* (Spn6B) is feasible and safe in older adults.
Secondary	1. If co-infection with LAIV and Spn6B alter upper and lower respiratory tract infection symptoms<br>2. How primary LAIV vaccination alters the:<br>(a) Risk of Spn6B carriage acquisition<br>(b) Density of Spn6B carriage<br>(c) Duration of Spn6B carriage<br>3. How primary Spn6B challenge or carriage alters the:<br>(a) Risk of secondary LAIV infection (i.e., detection of LAIV)<br>(b) Viral load of secondary LAIV infection (AUC of density over time)<br>4. If nasal colonisation with other respiratory viruses and bacteria is present<br>5. To evaluate changes in nasal cells phenotype, function and gene expression over time
Exploratory	1. To identify if primary Spn6B challenge or carriage alters immune responses to secondary LAIV vaccination (innate and adaptive) and vice-versa.<br>2. To determine T cell receptor and B cell receptor sequence at the single cell level in nasal cells<br>3. To evaluate changes in inflammatory markers and antibody responses to pneumococcus and LAIV in the mucosa<br>4. To compare selected immune parameters both at the cellular and transcriptomic level between the nasal mucosa and systemic circulation<br>5. Comparison of immune response to LAIV vaccination over the life course (children, younger adults, older adults)<br>6. Comparison of immune and transcriptional parameters of experimental co-infection with Spn6B and LAIV with data obtained from natural infected older adults (symptomatic and asymptomatic)<br>7. To determine the frequency, phenotype, and function of immune cells in axillary secondary lymphoid tissue compared to blood and nasal mucosal following intranasal immunisation, in both older and younger adults.<br>8. To characterise transcriptional changes in immune cells (single cell and bulk RNA sequencing) in response to LAIV and Spn6B challenge/carriage/vaccination.
Intervention(s)	Intranasal inoculation with Spn6B suspension<br>Vaccination with Live Attenuated Influenza Vaccine (LAIV)<br>Fine needle aspiration of axillary lymph nodes under ultrasound guidance (in a subset of up to 10 individuals)

4. ABBREVIATIONS

CAP	Community-Acquired Pneumonia
CI	Chief Investigator
CRF	Case Report Form

EHPC	Experimental Human Pneumococcal Carriage
FNA	Fine needle aspiration
GCP	Good Clinical Practice
GP	General Practitioner
HRA	Health Research Authority
ICF	Informed Consent Form
IPD	Invasive Pneumococcal Disease
LAIV	Live Attenuated Influenza Vaccine
NHS	National Health Service
NW	Nasal Wash
OM	Otitis Media
PBMC	Peripheral Blood Mononuclear Cell
PCR	Polymerase Chain Reaction
PI	Principal Investigator
PIS	Participant/ Patient Information Sheet
qPCR	Quantitative Polymerase Chain Reaction
REC	Research Ethics Committee
RES	Research Ethics Service
RNA	Ribonucleic Acid
SAE	Serious Adverse Event
SUSAR	Suspected Unexpected Serious Adverse Reactions
SOP	Standard Operating Procedure
TDS	Three times a day
TOPS	The Over Volunteering Prevention System database
UKHSA	United Kingdom Health Security Agency

5. BACKGROUND AND RATIONALE

Pneumococcal Disease and Impact

Community-acquired pneumonia (CAP) has an incidence of up to 14 cases per 1,000 adults and is responsible for 4 million deaths annually. Bacterial and viral lower respiratory infections are the fourth leading cause of morbidity and mortality worldwide at 2.4 million deaths per year (Lavelle & Ward, 2022). Children under 5 years old and adults over 65 years old are particularly at risk of suffering from severe or fatal respiratory diseases.

Streptococcus pneumoniae is a common colonizer of the nasopharynx with approximately 10-20% of adults colonised at any given time. Colonisation is largely asymptomatic but a pre-requisite for transmission and disease. *S. pneumoniae* is the most common cause of CAP and invasive pneumococcal disease (IPD) and is responsible for a significant burden of morbidity and mortality worldwide, especially in young children and the elderly (Tsoumani et al., 2023). Why some individuals develop pneumococcal disease when exposed whereas others asymptomatically carry the bacteria in their nose is poorly understood. However, there is increasing evidence that viral co-infection and alterations in host immunity are associated with increased risk of pneumonia and invasive pneumococcal disease.

Secondary pneumococcal pneumonia is a major cause of mortality following seasonal and pandemic influenza infection (Chien et al., 2009). Seasonal increases in infections with Respiratory Syncytial Virus (RSV) and influenza in young children are strongly associated with subsequent increased hospital admissions with invasive pneumococcal disease (Weinberger et al., 2015, 2013).

During the COVID-19 pandemic, social restrictions and face masks created ideal conditions to study associations between respiratory viral infections, pneumococcal carriage and IPD. Studies in Israel demonstrated that whilst rates of pneumococcal carriage remained stable during non-pharmaceutical interventions, cases of CAP and IPD substantially decreased during this period. The reduction of invasive pneumococcal disease was temporally associated with decreases in RSV, Influenza and human metapneumovirus cases providing epidemiological evidence of an interaction between pneumococcal and viral co-infections.

Similarly, healthy children vaccinated with the live attenuated influenza vaccine (LAIV) experience a temporary increased pneumococcal colonisation (Thors et al., 2016). Together these data suggest that bacterial and viral co-infection alters the immune response in the nasal mucosa resulting in an increased risk of transmission and disease. To better understand the impact of co-infection on nasal immune responses, we investigated co-infection of Influenza and pneumococcus in the upper respiratory tract using our experimental human pneumococcal challenge model.

The Experimental Human Pneumococcal Colonisation (EHPC) model

Over the past decade we have developed a unique controlled human infection model with pneumococcus in which 40-70% of the participants nasally inoculated with pneumococcus develop nasal colonisation for 1-4 weeks (Robinson et al., 2023). The Experimental Human Pneumococcal Colonisation (EHPC) programme has been running safely in the UK for thirteen years. Over 2000 participants including adults with moderate asthma and healthy adults aged 50-84 (Robinson et al., 2023) have been inoculated to date without safety concerns. The study team have gained extensive knowledge in pneumococcal infection responses, correlates of protection and mucosal immunity (Wright et al., 2013; Carniel et al., 2021; Mitsi et al., 2018, 2020; Peno et al., 2018; Diniz et al., 2022). This work has demonstrated that prior carriage boosts pneumococcus-specific IgG responses as well as cellular responses in the lungs and blood (Wright et al., 2013). Serotype-specific memory B cells were associated with protection from reacquisition of carriage following re-challenge studies (Robinson et al., 2023; Pennington et al., 2016; Jochems et al., 2019).

EHPC in older adults

We recently reported results of a controlled human infection challenge study where we exposed healthy older adults (over 50 years) to pneumococcus. We observed in older adults that colonization rates and densities were not different from that of younger adults, but their immune response differed – those who became colonized did not exhibit the expected boost in specific IgG against the inoculated strain capsular polysaccharide (CPS), as seen in younger adults. In addition, in those participants who were challenged and did not become colonized, IgG levels to the CPS decreased a month after challenge. Re-challenging study participants with homologous pneumococcus 6B up to a year later did not protect them from colonization, unlike in younger adults where re-challenge conferred protection (Adler et al., 2021; Ferreira et al., 2013). Together, these findings suggested a diminished immunizing effect of pneumococcal colonization in older adults, potentially increasing their vulnerability to severe pneumococcal disease even with lower colonization rates.

EHPC of Influenza and pneumococcal co-infection in younger adults

We have used our CHIM to study interactions of pneumococcus and live attenuated influenza vaccine (LAIV) (Rylance et al., 2019). We demonstrated that the effect of pneumococcal colonisation on the outcome of infection and immune responses to influenza antigens is dependent on the order of pathogen exposure. Attenuated influenza infection prior to challenge with pneumococcus led to exacerbated nasal inflammatory responses and impaired nasal cellular responses, particularly affecting monocytes, which was associated with increased pneumococcal bacterial carriage density (Jochems et al., 2018). Surprisingly, pre-existing pneumococcal colonisation with pneumococcus at the time of attenuated influenza virus infection reduces nasal inflammatory responses leading to decreased nasal antibody responses, as well as lung cellular responses to influenza antigens (Carniel et al., 2021).

We postulate that pneumococcal colonisation may alter antigen presentation at the nasal epithelia reducing immune responses to subsequent viral infections. PCV vaccination, which reduces pneumococcal colonisation, could therefore have an indirect effect on protection against respiratory infections. Data in the literature also supports this. Post hoc analysis of two randomised-controlled trials (RCTs) found that individuals vaccinated with PCVs had 30–35% reduction in hospitalisations for the endemic human coronaviruses (hCoV OC43, and HKU1) associated with pneumonia in adults (Frasca et al., 2020; Mogilenko et al., 2022) and LRTI in children. PCV13 vaccination in older adults was associated with a reduction of approximately 30% in COVID-19 disease, hospitalisation and death (Lewnard et al., 2022).

This work poses important questions on whether the synergy of respiratory infections with viruses and bacteria can lead to more severe disease, what nasal immune mechanisms are associated with transmission and whether transmission can be curbed by improved vaccination strategies.

Pneumococci are covered in a polysaccharide capsule with over 100 different capsular serotypes described. Pneumococcal Conjugate Vaccines (PCV), targeting the polysaccharide capsule of 13 serotypes commonly causing disease, have demonstrated that effective prevention of both pneumococcal colonisation and disease is possible in young children. PCVs also provide the additional benefit of indirect protection of unvaccinated adults due to reduction of circulating pneumococcus following vaccination of children. The PCV vaccine has been shown to protect again mucosal colonisation of vaccine associated serotypes but may leave room for other serotypes to predominate. (Bewick et al., 2012; Hammitt et al., 2006; Miller et al., 2011). PCV vaccines covering a wider range of serotypes have recently been licensed (PCV-20).

Immune Response and Age

Older adults are at an increased risk of pneumococcal disease although the rates, duration and densities of *S. pneumoniae* colonization in the upper respiratory tract are comparable or lower than those observed in younger adults (Adler et al., 2021; Almeida et al., 2014). The reasons underlying the increased risk of pneumococcal disease are poorly understood but may be associated with alteration in immune responses including innate immune responses such as neutrophil recruitment into tissue, reduction in phagocytosis, killing of bacteria and antigen presentation by myeloid cells (Bleve et al., 2023) and the adaptive immune response with an increase in cytotoxic CD4+ and CD8+ T cells and an increase in age-associated B cells in peripheral circulation (Frasca et al., 2020; Karagiannis et al., 2023; Mogilenko et al., 2022). We have recently shown that older adults who are prone to colonisation with pneumococcus show increased inflammation at the nasal mucosa before experimental colonisation. We hypothesise that nasal inflammation results in tissue injury and increased adhesion of pneumococcus (Urban et al., 2023).

Study Rationale

This study intends to investigate the recruitment and activation of cells in the nasal mucosa over the course of infection (challenge with pneumococcus) and vaccination with Influenza in older adults (>55 years). The use of a cold-adapted influenza virus, such as LAIV, will provide a safe platform to study influenza nasal effects without any risk to older adult participants. Considering LAIV leads to profound inflammation in the nose and impaired responses in the nose and lung to pneumococcal carriage in young adults, we will compare this with an older cohort. Since older adults show increased nasal inflammation, we postulate that inflammatory responses due to co-infection will be even more exacerbated in older adults leading to increased virus shedding and pneumococcal density in the nose and oropharynx (throats). In this clinical study, we will extend this LAIV-EHPC co-infection model to the elderly population (aged >55 years) to assess nasal immune responses to LAIV and pneumococcus carriage, as well as the effect of the co-infection in this population. We will conduct two studies under the same protocol. Those studies will reflect alternative scenarios:

1. Colonisation with Pneumococcus first (LAIV administered 3 days after colonisation with pneumococcus)
2. Immunisation first (LAIV precedes nasopharyngeal inoculation with pneumococcus by 3 days).

In parallel, and to obtain data on nasal immune responses to infection with LAIV alone, we will vaccinate a control group of younger and older adults with LAIV and monitor their immune responses in a comparable manner to the co-infection study. These data will allow us to understand which immune responses are triggered by LAIV alone and whether the quality or quantity of immune responses to LAIV or viral shedding differ between younger and older adults.

The results of the study will provide a better understanding of the factors that influence susceptibility to infection with either pathogen and their interaction. We will undertake an in-depth analysis of immune responses in the upper respiratory tract, peripheral circulation and lymph nodes and their association with protection or susceptibility to colonisation/infection. The obtained knowledge will contribute to the design of intervention that could be tailored to an at-risk population of older adults through vaccination and/or early treatment with antivirals. A better understanding of immune response at the nasal mucosa is of

particular interest for the design of mucosal vaccines that reduce the risk of dissemination of respiratory pathogens into the lower respiratory tract.

6. OBJECTIVES AND OUTCOME MEASURES

Objectives	Outcome Measures
**Primary Objective:**<br><br>1. To determine if a co-infection human challenge model with LAIV and S. pneumoniae (Spn6B) is feasible and safe.	1. Absence of SAEs/AESIs relating to inoculation throughout the study
**Secondary Objectives**<br><br>1. If co-infection with LAIV and Spn6B alter upper and lower respiratory tract infection symptoms<br>2. How primary LAIV vaccination alters the:<br>(a) Risk of secondary Spn6B carriage<br>(b) Density of secondary Spn6B carriage<br>(c) Duration of secondary Spn6B carriage<br>3. How primary Spn6B challenge or carriage alters the:<br>(a) Risk of secondary LAIV infection (i.e., detection of LAIV)<br>(b) Viral load of secondary LAIV infection (density over time)<br>4. If nasal colonisation with other respiratory viruses and bacteria is present<br>5. To evaluate changes in nasal cells phenotype, function and gene expression over time	1. Symptomatology using diary cards, with clinical review of LRTI and URTI symptoms at visits<br>2. Classical culture and molecular methods including RT-qPCR<br>(a) Colonisation rates of Spn6B carriage<br>(b) Density of Spn6B carriage<br>(c) Longitudinal quantification<br>3. Secondary LAIV infection and viral load determined by RT-qPCR and/or microfluidic qPCR<br>4. Detection of most common respiratory viruses and other respiratory bacteria will be assessed by microfluidic qPCR and/or qPCR<br>5. Analysis of changes in cell populations and gene expression levels at different time points in nasal mucosa using flow cytometry and RNA sequencing

**Exploratory Objectives**	
1. To identify if primary Spn6B challenge or carriage alters immune responses to secondary LAIV vaccination (innate and adaptive) and vice-versa.<br>2. To determine T cell receptor and B cell receptor sequence at the single cell level in nasal cells.<br>3. To evaluate changes in inflammatory markers and antibody responses to pneumococcus and LAIV in the mucosa<br>4. To compare selected immune parameters both at the cellular and transcriptomic level between the nasal mucosa and systemic circulation<br>5. Comparison of immune response to LAIV vaccination over the life course (children, younger adults, older adults)<br>6. Comparison of immune and transcriptional parameters of experimental co-infection with Spn6B and LAIV with data obtained from natural infected older adults (symptomatic and asymptomatic)<br>7. To determine the frequency, phenotype, and function of immune cells in axillary secondary lymphoid tissue compared to blood and nasal mucosa following intranasal immunisation, in both older and younger adults.<br>8. To characterise transcriptional changes in immune cells (single cell and bulk RNA sequencing) in response to LAIV and Spn6B challenge/carriage/vaccination.	1. Assessment of immune responses including nasal cytokines, cell populations, Influenza- and Spn6B-specific antibodies and cellular immunity before and after each pathogen challenge.<br>2. T cell receptor and B cell receptor sequencing in parallel with scRNAseq to determine clonotypes in the nasal mucosa in response to LAIV/Spn6B co-infection in selected samples<br>3. Analysis of changes in antibody levels and inflammatory markers at different time points in nasal lining fluid, saliva and/or throat swabs<br>4. Analysis of antibody level, inflammatory markers and cell populations (cellular and transcriptome level) in systemic circulation and compare data with those generated at the nasal mucosa<br>5. Meta analysis of data generated in this study with comparable data generated in a parallel study on mucosal immune responses to infection with LAIV in children<br>6. Meta analysis of data generated in this study with comparable data generated by collaborators in a hospital study (University of Geneva).<br>7. Single cell ribonucleic acid sequencing (ScRNA-seq) and/or multiparameter flow cytometry of lymph node cells and comparison with similar data generated from the nasal mucosa and blood<br>8. ScRNAseq and bulk transcriptome analysis to compare gene expression in response to Spn6B and LAIV co-infection or LAIV infection alone

7. STUDY DESIGN

This will be a single blind randomised controlled human co-infection study using a previously established pneumococcal and LAIV infection model (Jochems et al., 2018). This study will be conducted in Oxford. Eligible healthy, older adults will receive an intranasal challenge with LAIV/saline on Day –3 followed by challenge with Spn6B on day 0. Participants will receive a further intranasal challenge on Day 3 with the LAIV/saline option they did not receive on Day –3 (participants who received saline at day –3 will receive

a LAIV on day 3, and those who received LAIV will receive saline). All study participants will be blinded to the order of challenge agents (LAIV first or saline first).

We will be using LAIV outside of the licencing recommendations (children under the age of 18). LAIV is restricted to infection of the upper respiratory tract with no replication and therefore deemed safe in older adults. Safe experimental human challenge models of pneumococcal inoculation have been conducted previously in Liverpool and is established as an outpatient model (>2000 participants have completed the programme with no evidence of community transmission) (Robinson et al., 2023).

In addition to the co-infection model, we will vaccinate a control group of younger and older adults with intranasal LAIV only to determine the impact on systemic and mucosal immune responses. Data form the control group will distinguish the effect of LAIV vaccination alone on nasal and systemic immune responses from the effect of co-infection Spn. We will assess immune responses in the axillary lymph node tissue in a subset of participants across Group A and B (up to 10 volunteers).

Allowing for a 20% expected drop out, we will enrol up to 60 participants to Group A to complete at least 40 participants. We may stop recruitment once we have the required datasets from 40 participants. A participant will be considered enrolled in the study at point of collection of research samples at Baseline Assessment (see section 9.7.1).

<u>**Study Visits**</u>

Group A:
Healthy older adults age 55-80 years, n=40-60

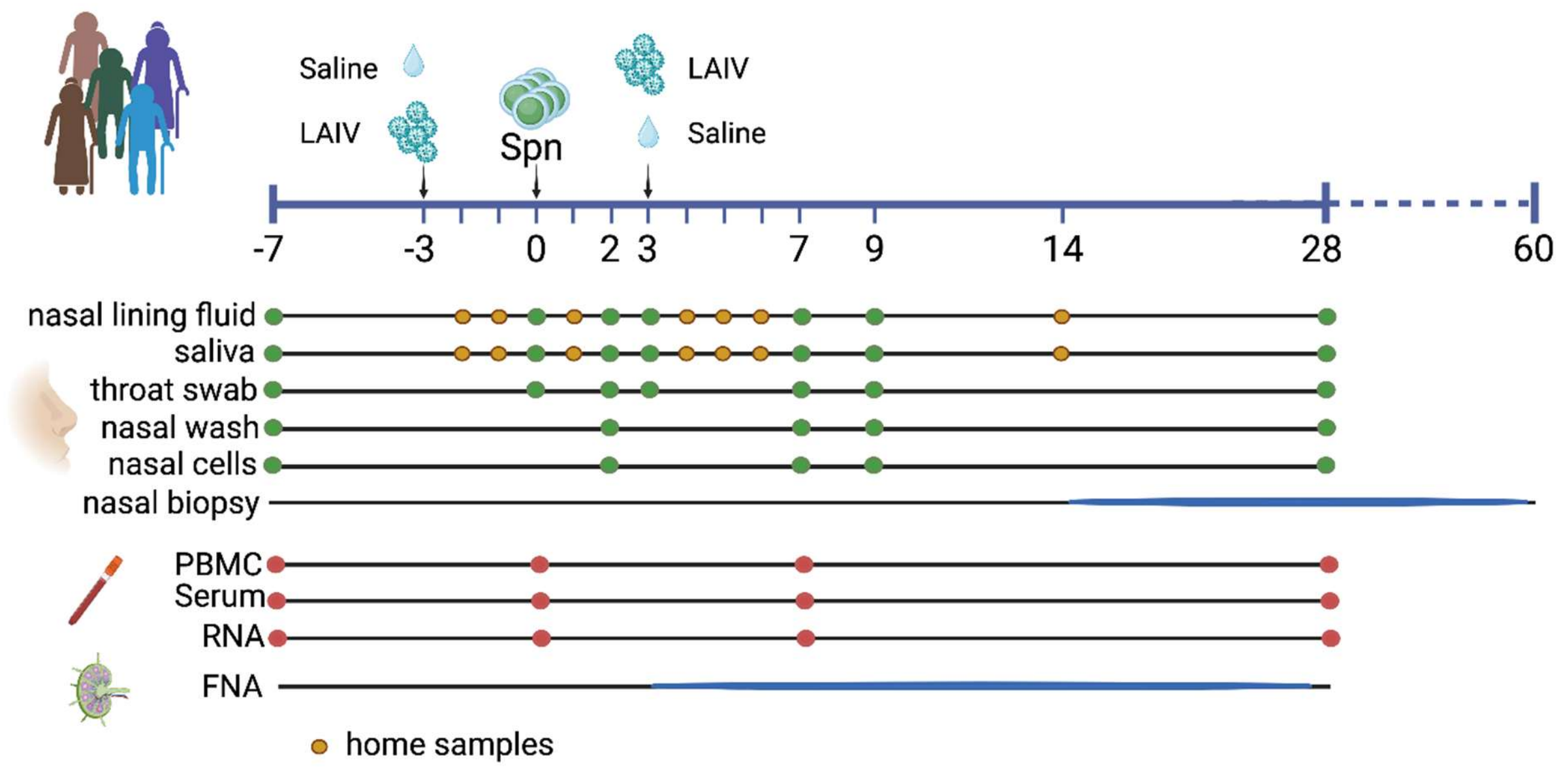

- **LAIV=Live attenuated influenza vaccine. Spn=*Streptococcus* pneumoniae. PBMC=Peripheral Blood Mononuclear Cell. RNA=Ribonucleic acid. FNA=Fine needle aspirate**

  Figure 1: Clinical study visits and sampling to be conducted for participants in group A (active study arm).

Group B – LAIV only (control arm):

Healthy younger adults age 18-49 years, n=7-10

Health older adults age 55-80 years, n=7-10

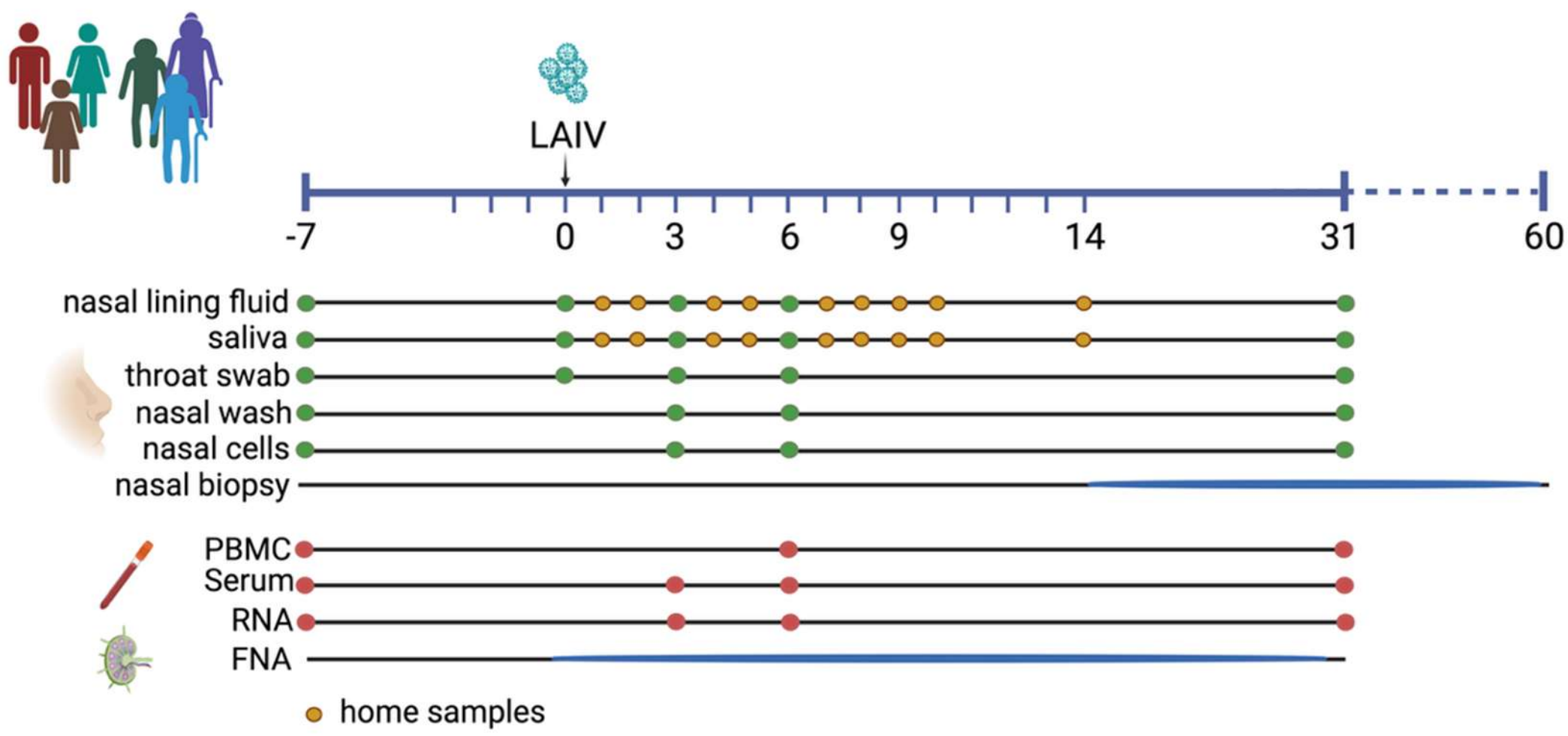

- **LAIV=Live attenuated influenza vaccine. PBMC=Peripheral Blood Mononuclear Cell. RNA=Ribonucleic acid. FNA=Fine needle aspirate**

Figure 2: Clinical study visits and sampling to be conducted for participants in group B, those receiving LAIV only.

Analysis of nasal mucosal responses will be performed in the following sample types:

1. Nasal cell samples for single cell RNA sequencing (scRNAseq), bulk RNA sequencing and analysis of nasal cell phenotypes and function to identify immune responses in the nasal mucosa in response to LAIV infection alone and co-infection of LAIV and Spn6B.
2. Nasosorption samples to evaluate age-associated inflammation and antibodies to pneumococcal and LAIV antigens as appropriate
3. Nasal washes to detect colonisation by pneumococcus by classical microbiology and molecular methods, LAIV by molecular methods, measure mucosal antibody responses against pneumococcus and LAIV antigens and other soluble markers of cell activation
4. Throat swab; saliva sample to detect viral and bacterial load by molecular methods.

Additionally, blood samples will be collected for safety assessment and as a comparator to nasal immune responses. In selected study participants, fine needle aspirates of axial lymph node cells will be obtained to compare lymph node responses with those in the nasal mucosa and blood. In selected study participants (up to 5 participants in group A and up to 5 in group B older adults) we will perform optional nasal biopsies to evaluate remodelling of the nasal mucosa due to pneumococcal carriage and/or LAIV infection using spatial transcriptomic. Clinical review and a patient diary will collect symptomatology throughout the study.

8. PARTICIPANT IDENTIFICATION

8.1 Study Participants

The participants are split into Group A and a control Group B. The study staff will determine eligibility at the screening visit. Participants will not have had an influenza vaccine in the current flu season or suffered from confirmed influenza in the past 2 years. They will not have had a pneumococcal polysaccharide vaccine in the past 1 year or a pneumococcal conjugate vaccine in the past 3 years.

Group A participants enrolled on this study will be up to 60 healthy volunteers between 55 and 80 years of age. Group B will include 7-10 healthy volunteers between 18 and 49 years of age, and 7-10 healthy older adults between 55 and 80 years of age.

8.2 Inclusion Criteria (Group A and B)

- Participant is willing and able to give informed consent for participation in the study.
- Healthy adults, between 55-80 years OR 18-49 years (Group B only).
- In the Investigator's opinion, is able and willing to comply with all study requirements.
- Fluent spoken English – to ensure a comprehensive understanding of the research study
- Willing to allow their General Practitioner and consultant, if appropriate, to be notified of participation in the study.
- Agree to provide their National Insurance/Passport number for the purposes of TOPS registration and for payment of reimbursement expenses.
- Participant must live near to study site or in the surrounding area
- Females of childbearing potential* with a negative urine pregnancy test at screening and willing to practice adequate contraceptive** measures as per UK Clinical Trial Facilitation Group during the study.

*This is applicable to Group B only. A woman is considered of childbearing potential, i.e., fertile, following menarche and until becoming post-menopausal unless permanently sterile. Permanent sterilisation methods include hysterectomy, bilateral salpingectomy and bilateral oophorectomy. A post-menopausal state is defined as no menses for 12 months without an alternative medical cause. A high follicle-stimulating hormone (FSH) level in the post-menopausal range may be used to confirm a post-menopausal state in women not using hormonal contraception or hormonal replacement therapy. However, in the absence of 12 months of amenorrhoea, a single FSH measurement is insufficient.

**Acceptable effective forms of contraception for female volunteers include:

- Established use of oral, injected or implanted hormonal methods of contraception.
- Placement of an intrauterine device (IUD) or intrauterine system (IUS).
- Total abdominal hysterectomy.
- Bilateral tubal occlusion.
- Barrier methods of contraception (condom or occlusive cap with spermicide).
- Male sterilisation, if the vasectomised partner is the sole partner for the subject.
- Sexual abstinence defined as refraining from heterosexual intercourse during the entire period of risk associated with the study interventions. The reliability of sexual abstinence needs to be

evaluated in relation to the duration of the study and the preferred and usual lifestyle of the subject.

Should a volunteer become pregnant during the trial this outcome will be recorded and the Sponsor and the DSMC will be notified if appropriate. They will be followed up for clinical safety assessment with their ongoing consent and in addition will be followed until pregnancy outcome is determined (to birth or end of pregnancy). No further non-essential trial procedures will be performed (i.e., vaccination or inoculation), however, procedures such as appropriate antibiotic treatment may be required if pregnancy detected following inoculation.

8.3 Exclusion Criteria

The participant may not enter the study if ANY of the following apply:

- Severe and/or uncontrolled cardiovascular disease, respiratory disease, gastrointestinal disease, liver disease, renal disease, endocrine disorder, or neurological illness, as judged by the Investigator (note, mild/moderate well-controlled comorbidities are allowed). Including but not limited to:
    - Asplenia or dysfunction of the spleen
    - Chronic respiratory disease (e.g., asthma [requiring medication (including salbutamol inhaler) within last 12 months], COPD, bronchiectasis and sleep apnoea)
    - Chronic heart disease (e.g., angina, ischaemic heart disease, chronic heart failure) – controlled and stable hypertension may be included
    - Severe chronic kidney disease (e.g., nephrotic syndrome, kidney transplant, requires dialysis)
    - Chronic liver disease (e.g., cirrhosis, biliary atresia, hepatitis)
    - Chronic neurological disease that limits mobility, bulbar or respiratory function (including stroke, Parkinson’s disease, dementia and multiple sclerosis)
    - Diabetes mellitus (including diet-controlled)
    - Receipt of immunosuppressive therapy such as anti-cancer chemotherapy or radiation therapy within the preceding 12 months or long-term systemic corticosteroid, Roaccutane, or disease modifying anti-rheumatoid drugs therapy (for more than 7 consecutive days within the 3 months prior to enrolment).
    - Individuals with cochlear ear implants
    - Individuals with major cerebrospinal fluid leaks (e.g., following traumatic, major skull surgery, or requiring CSF shunts)
    - Subjects with known or suspected immune deficiency (e.g., known IgA deficiency, immotile cilia syndrome, or Kartagener’s syndrome)
    - History of frequent nose bleeds
    - Bleeding disorders
    - No major pneumococcal illness requiring hospitalisation in the last 10 years
- Maternal (Group B)
    - Female participants who are pregnant
    - Female participants who are lactating
    - Female participants who intend to become pregnant during the study

    - Female participants unable to take contraception measures during the study (from consent to final study visit)
- Close contact with individuals at increased risk of pneumococcal disease (e.g. children under 5 years, immunocompromised individuals) (Group A only).
- Healthcare workers in a direct caring role for individuals at increased risk of pneumococcal disease (Group A only).
- On medication that may affect the immune system in any way e.g. steroids
- Taking long term antibiotics, nasal/inhaled steroids, oral antiplatelets or warfarin therapy.
- Allergy to penicillin, amoxicillin, and/or gentamicin (Group A only)
- Allergy togelatin, lidocaine or any ingredient of the influenza vaccine.
- Current regular smoker/vaper (smokes daily) or previous regular smoker/vaper than has stopped smoking/vaping less than 12 months ago (up to 10 pack-years smoking history allowed).
- History of drug or alcohol abuse (frequently drinking over the recommended alcohol intake limit: men and women should not regularly drink more than 14 units per week)
- Any clinically significant finding on screening investigation bloods
- Not able to make specific inoculation/vaccination visit dates required for the study, or overseas travel booked for 21 days following baseline testing.
- Received any influenza vaccine in the same winter season as they are recruited
- Pneumococcal vaccination (which we can confirm is not the PCV vaccine) in the last 1 year (older adults who have had pneumococcal vaccination but not influenza vaccination may be considered for Group B – LAIV only).
    - Participants who have received the Pneumococcal Conjugate Vaccine specifically within the last 3 years (this would not be given routinely in the UK schedule).
- Previous involvement in a clinical trial with Pneumococcal inoculation in the last 3 years.
- Scheduled elective surgery or other procedures requiring general anaesthesia during the study.
- Participants who have participated in another research trial involving an investigational product in the past 12 weeks.
- History of significant unexplained bleeding after a surgical or dental procedure (for optional nasal biopsy participants only)
- Any other issue which, in the opinion of the study staff, may:
    - Put the participant or their contacts at risk because of participation in the study
    - Adversely affect the interpretation of the study results, or
    - Impair the participant’s ability to participate in the study

Temporary exclusion for inoculation

The following are temporary exclusion criteria to primary inoculation:

- Current acute infective illness – delay inoculation by 14 days
- Asymptomatic positive COVID-19 lateral flow test (taken on day of planned challenge) – delay inoculation by 21 days.
- Recent /current URTI OR Antimicrobial (including antiviral) use – delay inoculation by 28 days after last day of illness OR last date of antimicrobial therapy
- Received any other vaccination within the past 28 days
- Any study participant that is colonised with pneumococcus at the baseline visit (Day –7) will be excluded temporarily for 30 days and then this re-checked

- Temporary use of oral or inhaled steroids (within a month prior to enrolment)

Temporary exclusion for lymph node biopsy

The following are temporary exclusion criteria applicable to participants who have consented to lymph node biopsy (this is optional). On day of lymph node biopsy the following criteria will be reviewed:

- Any medicine that increases bleeding risk taken in the past 7 days (e.g. Aspirin)
- Signs of local infection or rash at the site of intended biopsy

For these participants, we would temporarily exclude from biopsy. We would consider rescheduling should the symptoms resolve, and they are still within the study window for FNA (between day 3 and last visit).

Temporary exclusion for nasal biopsy

- Antibiotic use (during the study)-delay nasal biopsy for at least 1 week from last date of therapy
- Dental infections- delay nasal biopsy for at least 2 weeks after last day of illness

9. PROTOCOL PROCEDURES

9.1 Recruitment

Participants in Group A and B will be recruited primarily in Oxford.

Advertisements for recruitment will be distributed through methods including but not limited to mail-out letter to primary care, posters, leaflets, websites, newspapers, radio, public engagement events, and/or social media, using advertising material containing wording from approved study documents to invite participation in the study. Potential participants may be contacted by methods including but not limited to email, telephone, and/or mail, using an approved invitation letter.

Where mail-outs are used (only with appropriate Confidentiality Advisory Group (CAG) approval), participants may be identified via the electoral open register, or through National Health Service databases using data extracts. For the NHS databases, initial contact with potential participants will not be made by the study team. Instead, study invitation material will be sent out on our behalf by an external company, CFH Docmail Ltd (or equivalent company), to preserve the confidentiality of potential participants. CFH Docmail Ltd (or equivalent company) is accredited as having exceeded standards under the NHS Digital Data Security and Protection Toolkit (ODS ID – 8HN70).

For mail-outs via the electoral register, the study team will obtain access to the names and addresses of individuals who are on the open electoral register (which contains the names of registered voters who have not opted out). In this instance, the study team will upload the mailing list to the CFH Docmail system (or equivalent company), and the study invitation pack will be sent out by CFH Docmail (or equivalent company).

Interested potential participants will be sent a copy of the participant information leaflet and invited to contact a member of the clinical research team if still interested in participating. Provided we have not exceeded our capacity for recruitment, potential participants will be given an unrestricted amount of time to decide whether to participate or not. Potential participants will be asked to demonstrate that they understand the studies objectives, associated risks and the possible benefits. If the study team are satisfied

that they meet the inclusion criteria, the potential participant will be invited to give their written informed consent and further clinical appointments will be made.

The details of other recruitment methods which may be used are outlined below:

- **Email campaign**: We may contact representatives of local tertiary education establishments and local employers and ask them to circulate approved posters and a link to the study website by email or hard copy.
- Oxford Vaccine Centre (OVC) database for healthy volunteers/other databases: The study may be advertised on the electronic newsletter sent out to those potential participants signed up to the Oxford Vaccine Centre's Healthy Volunteers Database. Additionally, by email distribution to potential participants registered on the OVC Healthy Volunteers Database or similar databases (where members of the public have given their consent to be contacted when studies open for recruitment and understand that this is not a commitment to participate), or to a group or list only with the express agreement of the network administrator or with equivalent authorisation. This would include services such as the Be Part of Research (BPoR) registry.
- **Media advertising**: Approved local media, newspaper and website advertisements may be placed in locations relevant for the target age group with brief details of the study and contact details for further information.
- **Website advertising**: Description of the study and copy of the information booklet may be placed on study websites and other appropriate platforms for advertising.
- **Social media**: Approved advertisements may be placed on study social media accounts or targeted social media platform advertisements including, but not restricted to, Twitter, Facebook and Instagram.
- **Exhibitions**: Advertising material and/or persons providing information relating to the study may exhibit using stalls or stands at exhibitions and/or fairs, such as University Fresher's Fairs.
- **SMS/text messages**: SMS/text message (or emails) may be sent to potential participants identified by GPs from their databases (which will require Participant Identification Centres [PIC] agreements to be set up with the GP surgeries).
- **Royal Mail Leaflet**: Royal Mail door-to-door service with delivery of invitation letters enclosed in envelopes may be sent to every household within certain postcode areas.

Research ambassadors and research staff will attend public engagement events as stated above to promote our research and engage the public. Potential participants interested in study participation are able to contact the sites by telephone, email, online or a reply slip.

Online Screening questionnaire

Information about the study will direct volunteers to the study website, where a full participant information sheet will be available. Volunteers will also have access to the study team contact details to communicate with the team directly. Volunteers who are willing to proceed will be asked to complete an initial online 3-part questionnaire.

Part 1: Will include major inclusion and exclusion criteria. If a volunteer is deemed ineligible based on any of the replies, they be informed, the questionnaires will stop, and no demographic information will be recorded. Those eligible will be directed to e-consent in part 2.

Part 2: e-consent for access to and storage of medical history and vaccination records (via the volunteer GP or NHS databases) and recording and storage of personal information. Completion of the e-consent directs the volunteer to part 3.

Part 3: Records demographic information, NHS number, medical history, and medication use.

Following review of NHS database or a GP summary by the research staff, volunteers that remain eligible will be invited for a full screening and consent visit. Their full eligibility will be assessed by a member of the clinical research team (see Figure 3 below). Clarification of history provided can be discussed with the volunteer by telephone, prior to the face-to-face screening visit. If further clarification is needed following the electronic database, a GP eligibility letter can be sent to request more information.

Where potential participants are not able or willing to complete the online screening and e-consent for storing and accessing medical records, they can be contacted to discuss the study in more detail. Medical records must be reviewed prior to screening, if they are still unable to consent to online screening they will be excluded.

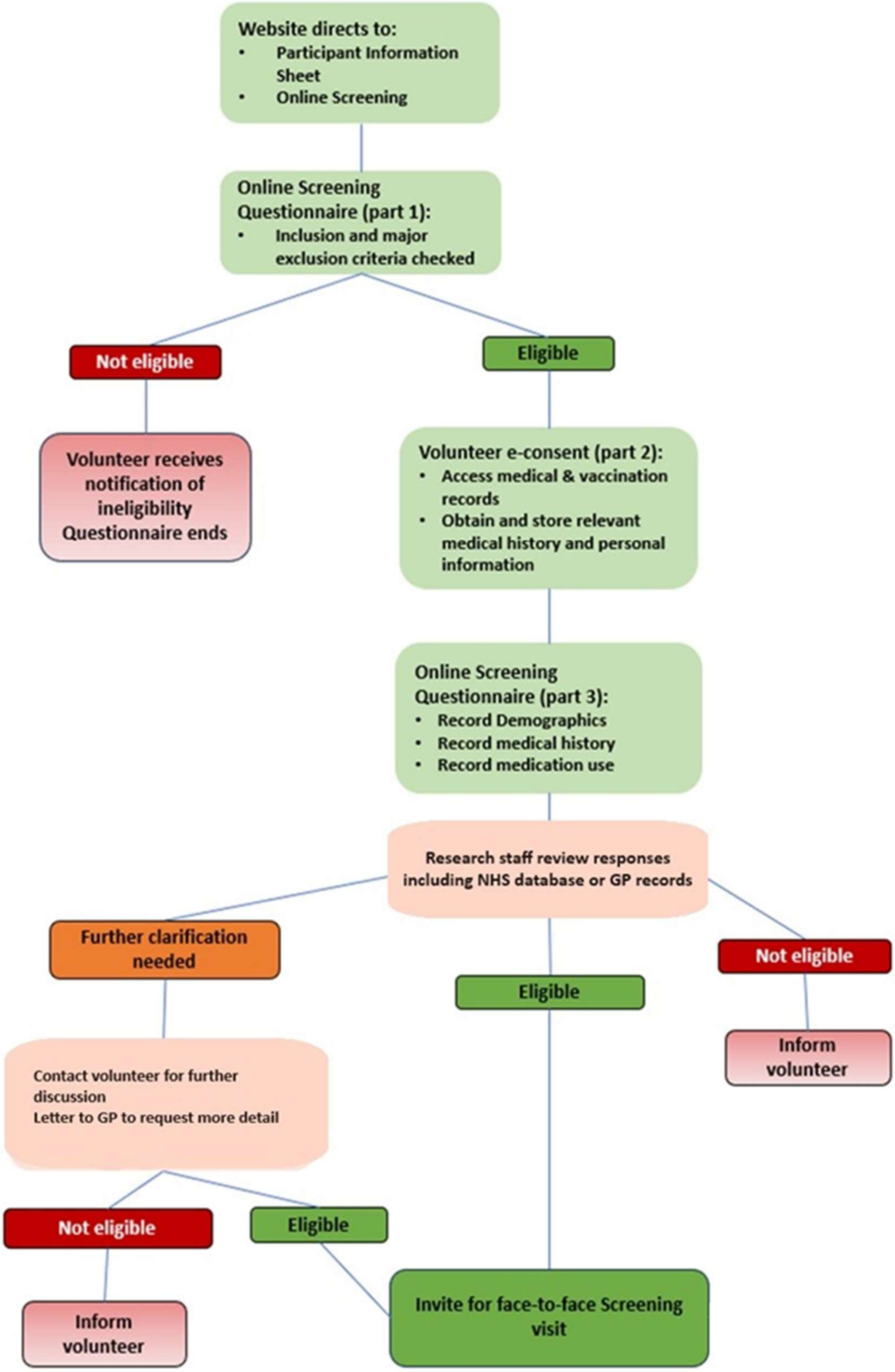

Figure 3: Online Screening Flow

9.2 Screening and Eligibility Assessment

Once an expression of interest has been received, an information sheet will be downloaded from the study website by the potential participant, and/or sent to them via mail or email. Following this information, if participants are willing to proceed, they will be asked to complete an initial online and/or telephone questionnaire before they are invited for a screening and consent visit, where their eligibility will be assessed by member of the clinical research team at the study site. Procedures will take place as listed in 9.6.1.

Permission to access the volunteer medical records either via the electronic patient record (EPR) or GP will be sought (if possible) prior to the screening visit via the online screening questionnaire. Alternatively, written permission to access medical records may be sought at the volunteers screening and consent visit.

Participants must satisfy all the approved inclusion and exclusion criteria, and no exceptions will be made. The following screening procedures will be undertaken:

- Participant demographics: e.g., age, sex, and ethnicity
- Medical history
- Contraception: participants of childbearing potential are asked if they are willing to use effective contraceptive measures for the duration of the study [Section 8.2 Inclusion Criteria (Group B)].
- Concomitant medications and vaccinations (including over the counter medications, vitamins, illicit drug use and herbal supplements)
- Recording of vital signs, including: resting pulse, oxygen saturation, blood pressure, oral temperature, weight and height to calculate body mass index (BMI).
- Physical examination including (but not limited to) cardiovascular, respiratory, abdominal, and gross neurological examination.
- Urine pregnancy test (participants of childbearing potential only)
- Screening Bloods: FBC, U+E, LFT, HbA1c, random plasma glucose (RPG), CRP, HIV, Hep B/C
- ECG (Group A only)
- Check TOPS database
- Next of kin contact details (Group A only)

Medical history, including vaccination and prescribed medication will be collected by participant recall and verified from the GP records or accessed via the electronic patient record (if available). The participant's GP will be notified of an individual's participation in the study. A volunteer identity check to be performed according to local policy.

Samples may be repeated at investigator discretion for safety or to check eligibility.

The medical tests carried out during the trial screening and follow up have the potential to find incidental medical problems that may require referral of volunteers for further investigation. Participants will be informed of these, and, with their consent, their general practitioner (GP) will be contacted.

If a participant is found to have hepatitis B or C, we would inform the participant, and the UK Health Security Agency (UKHSA). We would pass on the participant's name and personal contact information to the UKHSA. This is a legal obligation for medical professionals in the United Kingdom, and the participant will be made aware that they cannot opt out of this. This is made clear to participants in both the Participant Information Sheet (PIS) and the Informed Consent Form (ICF).

9.3 Informed Consent

The participant must personally sign and date the latest approved version of the Informed Consent form before any study specific procedures are performed. There will be separate consent forms for Group A and Group B. Fine needle aspiration will be on both consent forms as an additional optional choice. There will be a separate consent form for those participants wishing to get the optional nasal biopsy procedure.

Written and verbal versions of the Participant Information and Informed Consent will be presented to the participants detailing no less than: the exact nature of the study; what it will involve for the participant; the implications and constraints of the protocol; the known side effects and any risks involved in taking part. It will be clearly stated that the participant is free to withdraw from the study at any time for any reason without prejudice to future care, without affecting their legal rights, and with no obligation to give the reason for withdrawal.

Participants in Group A will be asked to complete a consent quiz as part of the informed consent process to ensure they have properly understood the study and provide an opportunity to review any areas that the participant may require further information before consent is taken. If they have more than 1 incorrect answer, they will have the opportunity to repeat the quiz as many times as necessary.

The participant will be allowed as much time as wished to consider the information, and the opportunity to question the Investigator, their GP or other independent parties to decide whether they will participate in the study. Written Informed Consent will then be obtained by means of participant-dated signature and dated signature of the person who presented and obtained the Informed Consent. The person who obtained the consent must be suitably qualified and experienced and have been authorised to do so by the Chief/Principal Investigator. A copy of the signed Informed Consent will be given to the participant. The original signed form will be retained at the study site.

As part of recommended practice (MRC tissue and biological samples for use in research) participants will be asked to consent to gift their samples for use in future studies and they may be shared with international collaborators. All samples will be anonymised. Any samples remaining at the end of the study will be transferred to the OVC biobank [Biobank REC number is 21/SC/0161] providing appropriate consent has been obtained.

9.4 Randomisation

Group A – Spn & LAIV Co-infection

The maximum time between screening and randomisation on Day -3 is 87 days (between day – 90 and day –3). Participants in Group A (older adults) will involve up to 60 participants randomised on a 1:1 ratio into 2 groups:

A1. Saline-P-LAIV arm: Participants will have saline on day –3*, S. pneumoniae* serotype 6B inoculation on day 0, followed by LAIV on day 3.

A2. LAIV-P-saline arm: Participants will have LAIV on day –3, *S. pneumoniae* serotype 6B inoculation on day 0, followed by saline on day 3.

This study is blinded to participants involved in the study. Random block sizes of 2 or 4 will be used. Randomisation will occur on the Day -3 visit by clinical staff to Group A1 or A2, once eligibility has been

established. Laboratory staff will be blinded to participant group. A computer-generated randomisation list will be generated by the study statistician. Randomisation software on REDCap will be used, and data will be stored on a secure electronic portal accessible by the statistician and IT team.

Group B – LAIV alone (control)

B1 = participants aged 18-49 years
B2 = participants aged 55-80 years

Both B1 and B2 receive LAIV alone on day 0. This group is not blinded or randomised.

Control Group Allocation

Participant allocation to Group A or B will not be randomised but will follow the rationale discussed below.

Younger adults between the ages of 18 to 49 will automatically be allocated to Group B. When adults between the ages of 55 and 80 years are screened; initially we will assign participants to Group A. In the event that the participant does not want to receive pneumococcal inoculation, but is still interested in being a part of the study, we will recruit these participants to Group B. We will ensure that 30 participants are assigned to Group A (15 in Spn/LAIV; 15 in LAIV/Spn arm) and a minimum of 5 participants are assigned to Group B before recruiting any further; so that a proportionate number of participants are recruited to both groups in the same flu season.

Depending on participant availability we may recruit more participants to both groups in the first or second year, while maintaining an appropriate ratio. All participants will be offered to consent for an FNA procedure in addition as an optional choice. Although all participants will be offered to consent, a maximum of 10 participants across all groups will have an FNA performed. This is not randomised and will depend on participant and clinic availability.

9.5 Blinding and code-breaking

Group A will be participant blinded for LAIV/saline administration on day –3 and day 3 respectively. The clinical research teams will not be blinded in this study. There is no blinding involved in Group B. Participant blinding is required to reduce the risk of bias on the reporting of adverse events depending on the order of administration of LAIV/Spn.

The administering doctor or nurse will prepare the vaccine out of view of the patient, in a separate room or by drawing a curtain. The syringe will be covered in silver foil, to disguise brand names. Once the nasal spray saline/LAIV is prepared, the participant will be blinded by using a physical disposable blindfold. If the participant is not happy to wear a blindfold, they can close their eyes for administration. The spray will then be administered.

Scheduled unblinding for Group A will occur after the last participant, in each season, has completed their final visit. Participant unblinding may also be performed at an earlier timepoint in the event of study withdrawal or the occurrence of SAEs, SAR or SUSAR (please see section 10).

If an SAE is deemed related to IMP, the CI/PI delegated investigator will assess expectedness against RSI. If this is deemed a potential SUSAR, the CI/PI delegated individual will unblind for reporting. Individual

envelopes with allocation to study arm will be assigned to all participants in Group A and kept securely as per local protocol.

9.6 Visit Summary

9.6.1 Baseline assessments

Following consent and eligibility assessment, these baseline assessments will be completed:

- Confirm ongoing verbal consent
- Vital signs including blood pressure
- Interim medical history including significant events
- Clinical examination
- ECG (Group A only)
- Check temporary exclusion criteria to challenge
- Baseline saliva, throat swab, nasosorption, nasal cells and nasal wash
- Safety bloods (If screening visit >21 days prior)
- Research bloods
- Check 24hr contact details (Group A only)

Participants in Group B of childbearing potential will need to donate a urine sample for urine HCG.

Subsequent visits occur according to the schedule of events for Group A and B listed below. The participant is considered enrolled following Day –7/Baseline assessment visit after research samples have been collected.

9.6.2 Schedule of Events – Group A

Study Visit<br>Day post Spn inoculation	Screening	Day -7	Day -3	-2	-1	Day 0	1	Day 2	Day 3	4	5	6	Day 7	Day 9	14	Day 28	Nasal Biopsy
**Visit window (days post Spn inoculation)**	**-90 to -5**	**-9 to -4**											**7 to 9**	**8 to 10**		**27 to 30**	**14 to 60**
Consent (Written)	X																(X)[&]
Consent (Verbal)	X	X	X			X		X	X				X	X		X	X
Study information	X																
Study screening (inc. medical history)	X																
Clinical Exam	X	X															
Vital Signs	X	X	X			X		X	X				X	X		X	X[%]
Screen for AEs			X			X		X	X				X	X		X	X
Concomitant medications	X	X	X			X		X	X				X	X		X	X
Screening bloods *	X																
ECG	X																
Randomisation			X														
Spn inoculation						X											
LAIV/saline[1] or saline/LAIV[2]			X[1]						X[2]								
Nasal biopsy[**]																	X
FNA									X (shaded: Day 3 to Day 28)								
Nasosorption ((X) home sampling)		X		(X)	(X)	X	(X)	X	X	(X)	(X)	(X)	X	X	(X)	X	
Nasal cells		X						X					X	X		X	
Nasal wash		X						X					X	X		X	
Throat swab		X				X		X	X				X	X		X	
Saliva ((X) home sampling)		X		(X)	(X)	X	(X)	X	X	(X)	(X)	(X)	X	X	(X)	X	
Research bloods		X				X							X			X	
Safety bloods		X[$]															

Table 1: Schedule of events – Group A *Screening bloods= FBC, U+E, LFT, HbA1c, RPG, CRP, HIV, Hep B/C, Coagulation (for nasal biopsy cohort only) [$]= **Safety bloods** (U+E, LFT, CRP, FBC) are only required during baseline assessment visit if screening bloods were performed >21 days beforehand. **Shaded text =** at any time between these dates depending on clinical staff and participant combined availability. [%] = Vital signs may be performed at the discretion of the ENT specialists. [&] = Safety follow-up: Participants after nasal biopsy procedure will be observed as per local NHS hospital guidelines before leaving the

facility. A phone safety review will be conducted by the study team at 1 day and 3 weeks after the nasal biopsy. Participants undertaking the optional nasal biopsy will undertake a separate additional written consent for the procedure which will be performed or re-confirmed on the day of the biopsy by the operator.**Optional procedure, up to 5 participants

9.6.3 Schedule of Events - Group B

**Study Visit**	**Screening**	**Day – 7/Baseline visit**	**Day**			**Day**			**Day**						**Day**	
**Day post LAIV**			**0**	**1**	**2**	**3**	**4**	**5**	**6**	**7**	**8**	**9**	**10**	**17**	**31**	**Nasal Biopsy**
**Visit window**	-90 to –2	–9 to –4							5 to 6						31 to 34	14 to 60
Consent (Written)	X															(X)[&]
Consent (Verbal)	X	X	X			X			X						X	X
Study information	X															
Study screening (inc. medical history)	X															
Clinical Exam	X															
Vital Signs	X	X	X			X			X						X	X[%]
Screen for AEs			X			X			X						X	X
Concomitant medications	X	X	X			X			X						X	X
Pregnancy test	X		X													
Safety bloods		X[$]														
Screening bloods*	X															
**LAIV**			X													
**Nasal Biopsy****																X
**FNA**										X						
Nasosorption ((X) home sampling)		X	X	(X)	(X)	X	(X)	(X)	X	(X)	(X)	(X)	(X)	(X)	X	
Nasal cells		X				X			X						X	
Nasal wash		X				X			X						X	
Throat swab		X	X			X			X						X	
Saliva ((X) home sampling)		X	X	(X)	(X)	X	(X)	(X)	X	(X)	(X)	(X)	(X)	(X)	X	
Research bloods		X				X			X						X	

Table 2: Schedule of events Group B. LAIV only, younger (18-49 years) and older (55-80 years) adults (n=7-10 each group) . ***Screening bloods=** FBC, U+E, LFT, HbA1c, random plasma glucose, CRP, HIV, Hep B/C, Coagulation (for nasal biopsy cohort only) [$] = **Safety bloods** (U+E, LFT, CRP, FBC) are only required during baseline assessment visit if screening bloods were performed >21 days beforehand. **Shaded text =** at any time between these dates depending on clinical staff and participant combined availability. [%] = Vital signs may be performed at the discretion of the ENT specialists. [&] = Safety follow-up: Participants after nasal biopsy procedure will be observed as per local NHS hospital

guidelines before leaving the facility. A phone safety review will be conducted by the study team at 1 day and 3 weeks after the nasal biopsy. Participants undertaking the optional nasal biopsy will undertake a separate additional written consent for the procedure which will be performed or re-confirmed on the day of the biopsy by the operator. **Optional procedure, up to 5 participants in the older group

9.6.4 Blood Volumes

At visits in Group A specified blood volumes up to the following maximum amounts will be taken:

Group A Visits	*Visit Window*	*FBC*	*U+E, LFT, CRP*	*HIV/Hep B, C, HbA1c, RPG*	*Coagulation (for nasal biopsy cohort only)*	*Research Bloods*	*Maximum Blood Volume*
**Screening**	-90 to -5	X	X	X	X		Up to 18.5 mL
**Day -7**	-9 to -4	X°	X°			X	Up to 87 mL
**Day 0**	0					X	Up to 72.5 mL
**Day 7**	7 to 9					X	Up to 72.5 mL
**Day 28**	27 to 30					X	Up to 72.5 mL
**FNA visit**	-3 to 30					X^	Up to 72.5 mL
						Total	Max 395.5 mL

Table 3: Blood Volumes Group A. X° these bloods are not required if Day –30 is less or equal to 21 days away from Day -7. ^Bloods taken at FNA visit can be combined with any visit between Day –3 and Day 30, if performed on the same day.

At visits in Group B specified blood volumes up to the following maximum amounts will be taken:

Group B Visits	*Visit Window*	*FBC*	*U+E, LFT, CRP*	*HIV/Hep B, C, HbA1c, RPG*	*Coagulation (for nasal biopsy cohort only)*	*Research Bloods*	*Maximum Blood Volume*
**Screening**	-90 to -2	X	X	X	X		Up to 18.5 mL
**Day -3**	-7 to -2	X°	X°			X	Up to 87 mL
**Day 3**	0					X	Up to 72.5 mL
**Day 6**	5 to 6					X	Up to 72.5 mL
**Day 31**	31 to 34					X	Up to 72.5 mL
**FNA visit**	0 to 34					X	Up to 72.5 mL
							Max 395.5 mL

Table 4: Blood Volumes Group B. ^Blood taken at FNA visit can be combined with another visit in which bloods are taken between day 0 and 34 if done on the same day.

In the event of an unscheduled visit for clinical assessment, safety bloods up to 15ml can be taken at investigator discretion during clinical review. This can be in addition to maximum blood volumes stated above.

9.7 Description of study intervention, study procedures (clinical) and concomitant medications

9.7.1 Description of Study Interventions

9.7.1.1 S Pneumoniae serotype 6B

Preparation of Spn6B challenge agent

The dilution of the inoculum will be prepared as per local SOPs in a room dedicated for challenge agent preparation. A mid-log broth culture of pneumococcus (Spn6B) will be frozen at -80°C in aliquots of glycerol-enriched media. Frozen aliquots will be thawed and checked for bacterial number (colony forming

units [CFU] per mL), and purity. These checks will first be carried out in research laboratories and then identification, purity by whole genome sequencing and penicillin sensitivity will be confirmed in a reference laboratory (UKHSA). On experimental inoculation days, aliquots will be thawed, washed twice, and re-suspended in 0.9% normal saline at the correct density. Once inoculum is prepared it will be administered within 30min to avoid degradation.

We will aim for a dose of 80,000 CFU/100µl per naris of the Spn6B inoculum (inoculum dose determined from previous challenge studies). We will allow a variation of half or double of this dose and have previously demonstrated in our dose-ranging studies that this range is safe and leads to similar colonisation outcome (rates and density recovered from the nasopharynx).

Maintenance, storage and transport of Spn6B challenge agent

Labelled pneumococcus (Spn6B) inocula will be stored at dedicated -80°C in the designated laboratory of the study sites. Prepared inocula are transported to the site clinical facilities in a clearly labelled vial and transportation bag. Inocula will be placed at room temperature within a closed polystyrene box and will not be opened before reaching the designated room of the research clinic in which volunteers are to be inoculated.

Supply and accountability

Accurate records of receipt and condition of all Challenge stock agents will be available for verification by the Study Monitor. Trained site staff will be responsible for adequate and accurate accounting of all Challenge Agents inoculum. Any deviation from the protocol-dispensing regimen will be fully documented.

Challenge procedure – Day 0 (Group A ONLY)

During this visit the study clinician will review the inclusion/exclusion criteria, screen for AEs, and check vitals. Study procedure will occur as listed in Table 1. Before challenge, a viral swab COVID-19 will be done with a point of care test to confirm participant negative status before inoculation.

The inoculum will be administered according to local Standard Operating Procedure (SOP). The participant will be seated in a semi-recumbent position. Using a P200 micropipette, 0.1 mL of pneumococcus-containing-fluid will be instilled into each nostril. This will be done slowly with sufficient interval between each inoculation (2-3 minutes) to ensure maximum contact time between the nasal and pharyngeal mucosa. After inoculation, the participant will remain in this position for up to 15 minutes. Participants will be asked not to wash or blow their noses for at least one hour.

Post Challenge

After the inoculation participants will be given a safety pack containing:

- Thermometer
- Safety information leaflet (including how to take temperature and symptoms of pneumococcal infection)
- Medical alert card with study team contact details
- Amoxicillin 500mg TDS 3-day supply (including antibiotic patient information sheet). Participants will be asked to return any remaining antibiotic doses to reconcile and discard.

Written and verbal instructions are given to the participant describing potential mild, moderate and severe symptoms and the instances when antibiotics can be taken. These include:

- At an investigator's discretion at any point
- At the end of the study, if they have been positive for Spn6B, at any timepoint following inoculation, without having had two consecutive negative NWs before the last visit
- If unwell and/ or symptomatic and instructed to take by the research team
- If unwell and unable to contact the research team

Participants will be instructed to monitor the development of any symptoms at home and complete diaries. Home monitoring of symptoms will include a clear flow chart of the necessary intervention should any symptoms develop (see participant safety information leaflet). A member of the research team will review diaries and assessments daily in the first week following inoculation and attempt to contact the participants should they not make contact or fill in the diary by the specified time. If no contact is possible, then a prior defined 'secondary contact', from whom written consent has been obtained (Group A only), will be telephoned. Participants will have access to a 24/7 on-call telephone number until the end of the study. Patients reporting symptoms potentially consistent with pneumococcal disease (for example, ear pain, sore throat, cough and fever) will be seen in person for medical assessment and will begin the course of amoxicillin if the research staff feel symptoms could possibly be due to pneumococcal or another potential bacterial infection.

To diminish the risk of local transmission we will educate personnel and participants about the characteristics, transmission, and risk of pneumococcal infection before starting the study, frequent handwashing and use of surgical facemasks will be applied.

Symptom directed clinical examination and investigation

If participant is experiencing adverse events/symptoms and a doctor's review is required then the participant may have an optional clinical examination during the visit at any time point for the duration of the study. Participants may be invited to attend an extra, or unscheduled visit for further clinical examination. Participants who present with sore throat symptoms will be assessed and graded in clinic using a pharyngitis assessment.

An optional, additional throat swab may be performed to identify potential causes of participants' symptoms. A BioFire respiratory PCR test will be an optional investigation to allow examination for and investigation of symptoms on a case-by-case basis. The BioFire test will be undertaken at the examining doctor's discretion to allow flexibility in the clinical assessment of the participant and sent to the NHS hospital lab for testing.

9.7.1.2 Live Attenuated Influenza Vaccine

The composition of live attenuated influenza vaccines differs annually based on the UK Guidance for the National Flu Immunisation programme. These may be tetravalent which protect against 4 strains of influenza or trivalent which protect against 3. Below we outline 3 LAIV medicinal product that were used in the UK as part of the national influenza immunisation program. The choice of LAIV for each season of this study will be based on the UK Guidance for the National Flu Immunisation programme and while the exact strains covered vary year to year, they will comply with WHO recommendation for the Northern Hemisphere.

Fluenz Tetra and FluMist Quadrivalent are licensed medicinal products in the UK for children under the age of 18 years. Both are produced by AstraZeneca, and in 2023/2024 had similar compositions (please see below).

They are both licensed for use in children up to the age of 18. During this study, we are using this medicine outside of licensing use, but there is ongoing data supporting safety in this population in previous trials.

Fluenz Tetra

Produced by AstraZeneca, Fluenz Tetra is a live attenuated influenza vaccine delivery via nasal spray. It is produced as a single use spray, with pre-made 0.2 mL suspension. It is administered to both nostrils (0.1 mL each), with no need to actively inhale or sniff. The product has a maximum shelf life of 18 weeks.

Strains covered 2023/2024 (composition may change in 2024/2025)	Fluorescent focus units
A/Victoria/4897/2022 (H1N1)pdm09 – like strain (A/Norway/31694/2022, MEDI 369815)	$10^{7+/-0.5}$
A/Darwin/9/2021 (H3N2) - like strain (A/Norway/16606/2021, MEDI 355293)	$10^{7+/-0.5}$
B/Austria/1359417/2021 - like strain (B/Austria/1359417/2021, MEDI 355292)	$10^{7+/-0.5}$
B/Phuket/3073/2013 - like strain (B/Phuket/3073/2013, MEDI 306444)	$10^{7+/-0.5}$

Table 5: Strains covered in 2023/2024 Fluenz Tetra LAIV vaccine

Flumist Quadrivalent

Similar to Fluenz Tetra, Flumist Quadrivalent is a live attenuated influenza vaccine delivered via nasal spray. It is produced as a single use spray, with pre-filled 0.2 mL suspension. It is administered to both nostrils (0.1 mL each), with no need to actively inhale or sniff. The product has a maximum shelf life of 18 weeks.

Strains covered 2023/2024 (composition may change in 2024/2025)	Fluorescent focus units
A/Norway/31694/2022 (H1N1) (A/Victoria/4897/2022 (H1N1)pdm09-like virus)	Total of $10^{6.5-7.5}$ FFU
A/Norway/16606/2021 (H3N2) (A/Darwin/9/2021 (H3N2)-like virus)	
B/Phuket/3073/2013 (Yamagata lineage)	
B/Austria/1359417/2021 (Victoria lineage)	

Table 6: Strains covered in 2023/2024 Flumist LAIV vaccine

Fluenz Trivalent

Produced by AstraZeneca, Fluenz Trivalent is a live attenuated influenza vaccine delivery via nasal spray. It is produced as a single use spray, with pre-made 0.2 mL suspension. It is administered to both nostrils (0.1 mL each), with no need to actively inhale or sniff. The product has a maximum shelf life of 18 weeks.

Strains covered 2024/2025 (composition may change in 2025/2026)	Fluorescent focus units
A/Victoria/4897/2022 (H1N1)pdm09 – like strain (A/Norway/31694/2022, MEDI 369815)	$10^{7+/-0.5}$
A/Thailand/8/2022 (H3N2) - like strain (A/Thailand/8/2022, MEDI 370626)	$10^{7+/-0.5}$
B/Austria/1359417/2021 - like strain (B/Austria/1359417/2021, MEDI 355292)	$10^{7+/-0.5}$

Table 7: Strains covered in 2024/2025 Fluenz Trivalent LAIV vaccine

Storage and Maintenance of LAIV

Both Fluenz Tetra and Flumist Tetravalent (or equivalent LAIV) will be stored in refrigerator between 2-8°C at each study site or in hospital pharmacy according to local guidelines. The temperature of the fridges will be monitored and logged. Vaccine stock will be accountable according to local policy.

The following should be adhered to:

- Fluenz tetra and Flumist Tetravalent (or equivalent LAIV) should appear colourless to pale yellow, there may be small white particles
- Any unused medicinal product or waste material should be disposed of in accordance with local requirements

Vaccination Process

Day –3 and Day 3 (Group A) AND **Day 0** (Group B)

During this visit the study clinician will review the inclusion/exclusion criteria, screen for AEs, and check vitals. Study procedure will occur as listed in Table 1.

Once eligibility to proceed is confirmed, the study nurse should prepare the LAIV out of view of the participant. They should check the expiry date and batch of the vaccine with a second checker. Check the appearance of the vaccine before administration. The suspension should be colourless to pale yellow, clear to opalescent. Small white particles may be present.

The participant should be told to breathe normally during administration, there is no need to inhale or sniff. Administer half the dose until the dose divider, then administer the remaining dose in the other nostril. The participant should be monitored for 15 minutes post procedure for anaphylaxis.

9.7.2 Study Procedures

<u>Blood sampling</u> will be performed by trained, experienced staff. Up to 87 mL of blood will be collected at a single visit to measure full blood count (for safety), and laboratory measures including, but not limited to serum immunoglobulins, PBMC populations, and host RNA expression.

<u>Fine Needle Aspirate (optional)</u>

Fine needle aspiration (FNA) will be carried out by an appropriately trained medical practitioner in an appropriate clinical facility with necessary equipment.

This procedure is optional and will be conducted for up to 10 participants. The visit must occur between Day 3 and Day 28 (Group A) or Day 0 to Day 31 (Group B). In Group A, only participants colonised with pneumococcus in their nasal wash sample following inoculation will be eligible. It can be conducted at a study visit or an unscheduled visit can be arranged in order to facilitate this. This visit will take approximately 30 minutes.

Eligibility to undergo the procedure will be confirmed, paying attention to:

- Blood thinning medication likely to induce bruising taken prior to aspiration
- Signs of local infection
- Pain or swelling at any sites of potential lymph node sampling
- Allergy to local anaesthetic
- Any other medical reason, which the practitioner deems significant to warrant exclusion from the FNA

Before the procedure, the participant's temperature, blood pressure, oxygen saturations and pulse rate will be recorded.

The FNA will be conducted using standard aseptic technique under ultrasound guidance. During the procedure, the ipsilateral and contralateral lymph nodes in the axilla will be located by physical examination of the lymphatic system, and then under US guidance. A sterile needle and syringe will be used to aspirate material from lymph nodes on one side or each side using 3-6 passes. Where necessary local anaesthesia will be employed to numb the area prior to sampling, using a standard local anaesthetic e.g., 1% lidocaine.

The visit for FNA biopsy may involve sampling lymph nodes from both axillae. Samples from right and left sides will be placed in separate specimen pots which have been clearly labelled to indicate the side from which the biopsy has been taken.

At each visit for FNA sampling a paired peripheral blood sample will be taken.

Lymph node samples will be placed into pre-prepared and labelled specimen pots and placed with the blood tubes in an appropriate transportation container. They will be transferred to the receiving laboratory where they will be processed upon receipt. The equipment necessary will all be made available on the day, including an ultrasound machine, and equipment for FNA (including disinfectant, local anaesthetic, needles, syringes, specimen tubes prepared with transport medium).

Participants will be observed for a minimum of 30 minutes after the procedure, and a final check of the FNA site at the end of this time, before participant leaves the visit.

Ultrasound imaging (as part of FNA procedure)
No device is being tested for the purposes of the research and the study does not include an investigational device. The device described herein is a tool to facilitate the research and perform the FNA.

A clinical grade ultrasound machine purchased and maintained for the purposes of research will be used during the study. There will be no endpoints directly related to assessment of the performance of the machine.

(a) *Device description*; A GE LogiqE10s, Toshiba Aplio i700 or similar ultrasound machine with appropriate probe for imaging soft tissues will be deployed for the study. A medicinal practitioner with training in its use will perform the ultrasound scan.

(b) *Device safety*: the ultrasound machine will be checked by the Clinical Research Facility electrician for use.

(c) *Maintenance and storage of device*: the US machine will be maintained, stored, and cleaned according to the manufacturer's instruction. Storage of the machine will be at the study site.

Oropharyngeal sampling:

1. **Saliva**

Participants should be fasted 30 minutes before collection. Saliva samples will be collected by requesting the participant to use the saliva collection method using Oracol devices. Ensure the Oracol lid is closed to

prevent leaking and transport to the lab in wet ice. This sample is taken to the lab and stored at -80 °C pending molecular testing.

2. **Throat swab**

The participant's tongue will be depressed using a tongue depressor exposing the palatopharyngeal arch. Two samples will be obtained: for bacterial identification, and for detection of pathogens by molecular and/or classical microbiological techniques, each by making 5 small circular motions of the palatopharyngeal arch in contact with the mucosa whilst avoiding the patient's tongue. Performing throat swabs prior to nasal washes will ensure that the oropharynx is not inadvertently contaminated with nasal pathogens prior to throat swab sampling.

3. **Nasosorption**

Concentrated nasal lining fluid will be obtained for analysis of soluble mediators using nasosorption strips (similar to blotting paper) developed by Hunt Developments Ltd (UK). Strips will be held inside each nostril for 1—3 minutes, then stored at -80°C pending further analysis.

4. **Covid Throat swabs**

Will be obtained for detection of SARS-CoV2 prior to first study intervention (Group A = Day -3, Group B = Day 0).

5. **Nasal wash**

After nasosorption, nasal wash will be performed using a modified Naclerio method (Naclerio et al., 1983). This has been used for more than 6 years and we have employed it in all of our pneumococcal carriage studies. Initially, 5 mL of saline is introduced using a syringe and held for a few seconds in the nose before being expelled into a sterile container. This is repeated twice in each nostril using 20 mL saline in total. In the event of nasal wash loss (defined as cough/sneeze/swallow) the procedure may then be repeated to obtain an adequate specimen (defined as ≥10 mL saline recaptured).

6. **Nasal cells**

Nasal cells may be collected using FloQ swabs and/or a nanosampling method in which cells are obtained through minimally invasive superficial nasal scrape biopsies (Rhino-Pro® curette). Participants can be biopsied multiple times with no significant side effects using the Rhino-Pro® curette method. Up to 6 samples will be obtained at each nasal sampling visit (2 samples for each nostril using Rhino- Pro® and/or one FloQ swab for each nostril). If no cells are visible on the Rhino-Pros following sampling, the sample can be repeated immediately. For participants sampled using the nasopharyngeal swab method, one FloQ swab will be used per nostril.

7. **Nasal biopsy tissue**

Nasal biopsy tissue will be collected on a single occasion as an optional procedure. This tissue will be collected from the inferior turbinate and/or the postnasal space/adenoid following NHS procedures. Participants will have up to 4 biopsies collected, performed by a trained ENT surgeon in an acute hospital outpatient setting using local anaesthesia.

Symptom Diary

Individual symptom scores will be accumulated for 21 days after Day -3 for group A and Day 0 for group B.

Upper Respiratory Tract Symptom Score

A total 'upper respiratory clinical symptom score' will be derived using a four-point scale (0-4 for absent, mild, moderate, severe and requiring emergency department visit or hospitalisation) for each of the following eight respiratory symptoms: sneezing, headache, malaise, fever (37.6°C or above/chills), nasal discharge, nasal obstruction, sore throat and cough according to established methods, giving a maximum clinical severity score of 24. This is an established method for studies of common cold illnesses (Jackson et al., 1958). Symptoms will be recorded at the same time of day and before any procedures such as nasal lavage is performed. More details are provided in Appendix A.

Lower Respiratory tract Symptoms

An e-diary of lower respiratory tract symptoms (D-3 to D17 in group A and D0 to D20 in group B) will also be completed with a scoring system outlined in the diary card (see Appendix A).

9.7.3 Concomitant medications

The use of concomitant medication prescribed or over the counter, will be recorded in the participants CRF. Prescribed medications such as antipyretics, antibiotics and immunosuppressive agents may impact the study results or may impact the safety of the participant (particularly during the challenge period). It is at the discretion of the study investigators to determine whether withdrawal or temporary exclusion of the participant is required.

Amoxicillin

Participants in Group A will be provided with amoxicillin 500mg to be taken TDS for 3 days to reduce density of Spn colonisation if they remain positive for Spn at their last visit without 2 consecutive negative samples, if unwell or at clinician discretion as per Section 9.7.1.1.

Lidocaine

A small subset of participants (up to 10) will be asked to provide an FNA sample; depending on participant and clinical availability. We will inject lidocaine locally as a regional anaesthetic for this procedure. This is licensed and commonly used for this purpose. We will ask participants if they have any contraindications to this medication as part of a separate consent process for the FNA procedure. It should not be injected into inflamed or infected tissues.

9.7.4 Post Study Vaccinations

Depending on vaccine availability, we may be able to offer the seasonal, annual COVID/influenza vaccine to participants. This would occur at the end of the final study visit (Day 28 in Group A; Day 31 in Group B). The intention is to prevent additional delay caused by the study for this group of older adults. The seasonal vaccines will be given as per Joint Committee on Vaccination and Immunisation (JCVI) guidance, which will be a COVID-19 vaccination and an Adjuvanted Quadrivalent Influenza Vaccine (aQIV or similar licensed flu vaccine).

Participants offered the opportunity to receive the seasonal vaccines for COVID-19 and Influenza will be recorded in the clinical database to ensure planning of post-study vaccination prescriptions. This will be recorded at the screening visit.

9.7.4.1 COVID-19 Vaccination

The participants may be offered a vaccine in line with the national vaccination programme. This is likely to be an mRNA COVID-19 vaccine.

The variant will be according to the latest JCVI guidance, which may vary year on year according to the evolving COVID-19 pandemic. For example, for the 2023-2024 season, Spikevax bivalent Original/Omicron BA.4-5 (50 micrograms/50micrograms)/mL dispersion for injection or Spikevax XBB.1.5 0.1 mg/mL dispersion for injection are recommended. One 0.5 mL dose contains two types of COVID-19 mRNA vaccine embedded in SM-102 lipid nanoparticles. The vaccine contains 1,2-Dimyristoyl-rac-glycero-3-methoxypolyethylene glycol-2000 (PEG2000-DMG) which is a potential allergen. It is presented as a multidose vial containing 2.5 mL dispersion in type 1 glass with a chlorobutyl rubber stopper and blue flip off plastic cap with aluminium seal.

Storage and Handling

The unopened vial can be stored for 9 months at -50°C to -15°C. Once thawed, the unopened vaccine may be stored refrigerated at 2°C to 8°C, protected from light, for a maximum of 30 days. The unopened vaccine can be kept at room temperature (8°C to 25°C) for up to 24 hours before use and once punctured, the vaccine must be used within 6 hours. Thawed vaccine cannot be refrozen.

9.7.4.2 aQIV Influenza vaccination (or similar equivalent)

The vaccine strains are updated every year in line with recommendations from the WHO. Vaccines currently used in the UK are quadrivalent (contain four subtypes) or trivalent (contain three subtypes); two A subtypes and two (or one for trivalent vaccines) B subtypes. The seasonal influenza vaccine for the study is Adjuvanted Quadrivalent Influenza Vaccine (aQIV), (surface antigen, inactivated), Seqirus suspension for injection in pre-filled syringe. One 0.5 mL dose contains 15 micrograms HA from four strains of influenza propagated in fertilised hens' eggs with adjuvant MF59C.1. It is licensed for prophylaxis of influenza in people aged 65 years and over.

Storage and handling

The aQIV vaccine (or similar equivalent) is stored in a refrigerator (2 °C – 8 °C) protected from light and must not be frozen.

9.8 Sample handling

9.8.1. Clinical laboratory samples

Blood will be drawn for the following laboratory tests. The processing and analysis of the blood will be carried out at an accredited clinical laboratory.

- Haematology:
    - Full blood count (including haemoglobin, platelet count, total white cell count, neutrophil count, lymphocyte count, eosinophil count)

- Biochemistry:
    - Urea and electrolytes (including sodium, potassium, urea, and creatinine)
- Liver function tests (including ALT, ALP, Bilirubin, Albumin)
    - HbA1c
- C – reactive Protein (CRP)
- Microbiology:
    - HbA1c and Random Plasma Glucose (RPG) – screening tests to exclude individuals with previously undiagnosed diabetes, as per National Institute for Health and Care Excellence (NICE) guidelines:
        - HbA1c of 48 mmol/mol (6.5%) or more.
        - Fasting plasma glucose level of 7.0 mmol/L or more.
        - Random plasma glucose of 11.1 mmol/L or more in the presence of symptoms or signs of diabetes.
- Diagnostic serology (screening only):
    - Screening tests for Hepatitis B, Hepatitis C and HIV infection (including: HBsAg, HCV antibodies, standard clinical HIV test in a laboratory, *e.g.,* 4th generation HIV antigen/antibody test HIV antibodies). Additional safety blood tests may be performed if clinically relevant at the discretion of the medically qualified investigator(s).

9.8.2. Immunology Research samples

University of Oxford Research Laboratories:

Research blood and nasal samples

Immunogenicity will be assessed by a variety of immunological assays. This may include single cell RNA-seq, CITEseq, TCR- and BCR-seq, ELISpot assays, flow cytometry assays, functional antibody assays and B and T cell analyses. Other exploratory immunological assays including cytokine analysis and other antibody assays, production of monoclonal antibodies, DNA analysis of genetic polymorphisms potentially relevant to vaccine immunogenicity and gene expression studies, amongst others, may be performed.

Other Research Laboratories
Collaboration with other specialist laboratories in the UK study, Europe and outside of Europe for further exploratory immunological tests may occur. This would involve the transfer of samples to these laboratories. The samples would remain pseudonymised. Informed consent for this will be gained from the volunteers at screening. Immunological assays will be conducted according to local SOPs.

Lymph node biopsy samples

These will be handled for processing similar to previous studies conducted at the Oxford Vaccine Group led by the study CI.

Immunogenicity will be assessed by a variety of assays such as single cell RNA-Seq, flow cytometry and serological and cell function analyses.

9.8.3. Laboratory Processes

Monitoring and confirmation of Colonisation of Spn6B
Colonisation will be defined by the result of nasal washes taken at 2, 7, 9 and 28 after inoculation as per visit schedule (Table 1). Therefore, samples will be taken at Days 2, 7, 9 and 28 after challenge.

Nasal washes will be plated onto culture media, incubated overnight and colonies will be confirmed as *S. pneumoniae* using classical microbiological techniques. If and when microbiological culture confirms *S. pneumoniae* experimental colonisation in the laboratory, the clinical team will be informed to ensure participants are counselled appropriately. A participant is considered positive for colonisation if they test positive for pneumococcus at any nasal wash sample by either classical microbiology or molecular methods. If study participants persistently carry pneumococcus day 28, they will be instructed to take oral Amoxicillin 500mg three times daily for 3 days to reduce the burden of colonisation.

DNA will be extracted from nasal wash (NW) samples using our well-defined protocols (German et al., 2019). *S. pneumoniae* serotype 6B detection will be done by multiplex qPCR for *lytA* and *6BcpsA*, respectively. This technique will enable us to detect individuals who are potential carriers with very low bacterial density. This multiplex qPCR is well established and validated in our laboratories. In addition, a microfluidic qPCR assay may be used.

Confirmation of viral load after vaccination
Nasal lining fluid and saliva will be tested for LAIV using an established qPCR assay and/or a microfluidic qPCR assay (Carniel et al., 2021).

Asymptomatic carriage of common respiratory viruses and bacteria
Viral and bacterial multiplex qPCR for detection and quantification all common respiratory viruses (including SARS-CoV2) and bacteria will be performed on genetic material of stored swab, nasal lining fluid, nasal washes or saliva as appropriate.

Antibody measurement
As per our previous published work we will measure LAIV-specific and pneumococcal serotype-specific antibody responses both systemically and at the respiratory mucosa. We will measure changes in responses to Spn6B compared to baseline in volunteers who received LAIV either before or after pneumococcal challenge. We will investigate the impact of LAIV alone on mucosal immune responses and on responses to Spn6B in the nasal mucosa and compare these to systemic responses in blood. Antibody responses to other respiratory pathogen will be evaluated as required.

Secretion of inflammatory markers in response to LAIV vaccination
Serum and mucosal lining fluid (nasosorption sample) will be stored at –80 at the OVG laboratories and thawed for batch processing to assess inflammatory markers. Samples will be analysed with a Luminex multiplex kit or similar for a wide range of cytokines and chemokines or by ELISA for additional markers

Cellular responses
Nasal cells and PBMCs will be stored in cryopreservation medium in liquid nitrogen in the OVG laboratories until batch processing to assess cellular and transcriptome responses.

We will also assess cellular responses to Spn6B and LAIV, as well as other respiratory pathogens as required, systemically (from PBMCs) and at the respiratory mucosa (nasal cells). We will assess T cell responses (including CD4+ and CD8+) post stimulation with pneumococcal and LAIV antigens and the levels of antigen-specific B cells. We will measure changes in the responses post-pneumococcal challenge to

baseline and the impact of LAIV infection alone, before or after challenge with Spn6B. We will also evaluate changes in the innate immune cell dynamics, activation and functionality in the nasal mucosa and correlate those findings with the quality and phenotype of B and T cell responses.

In a meta-analysis, mucosal and systemic responses in co-infected older adults will be compared to older adults who are asymptomatic carriers of pneumococcus or who suffer from pneumococcal pneumonia using data generated by our collaborator Arnaud Didierlaurent (University of Geneva, Switzerland) and to those in younger adults using existing data from a previous study (Jochems et al., 2018). Furthermore, we will compare mucosal and systemic immune responses to LAIV across the lifespan using data generated in a separate study in children vaccinated with LAIV and in younger and older adults (Group B).

Genetic responses

As per our previously published work, we will use single cell and bulk mRNA sequencing to perform in depth investigation of transcriptome changes in both peripheral blood and respiratory mucosal cells in response to co-infection with Spn6B and LAIV or LAIV alone. Gene signatures include single cells RNA sequencing as well as determination of T cell receptor and B cell receptor sequences. The gene signatures will be paired with immunophenotyping data and induction of immune responses after vaccination with LAIV assessed. This type of analyses will enable us to identify immune responses in the nasal mucosa in response to co-infection in older adults and correlate these with systemic responses. Furthermore, we can evaluate differences in mucosal and systemic immune responses between older adults co-infected with Spn6B and LAIV to older adults with either LAIV alone, asymptomatic pneumococcal carriage or hospitalised with pneumococcal pneumonia.

Spatial Transcriptomics

Nasal Biopsies will be embedded in formalin-fixed paraffin and analysed using the 10x Genomics Xenium platform. We aim for targeted profiling of >400 epithelial and immune transcripts at subcellular resolution. The data will allow us to generate spatially resolved models of nasal immunity in response to pneumococcal colonisation, LAIV infection or both.

9.9 Definition of Enrolment

The point of enrolment is defined as the day on which a Day -7 Baseline Assessment occurs (between day -9 and day -4) after research samples have been collected.

9.10 Early Discontinuation/Withdrawal of Participants

Each participant can exercise their right to withdraw from the study at any time without giving a reason. The participant information sheet (PIS) will inform participants that they may withdraw from the study at any time and that this will not affect any care they receive within the NHS. In addition to consent being withdrawn by a participant, the investigator may discontinue a participant from the study at any time for the following, although not exhaustive, reasons:

- The investigator considers it necessary for participant safety.
- Significant non-compliance with study requirements.

In circumstances pertaining to the safety of the participant, the DSMC chair, DSMC committee or Investigator may choose to discontinue further vaccination and/or specific study procedures for an

individual participant. However, participants should otherwise continue to attend the follow up visit schedule and follow up procedures unless they withdraw consent for this. Such circumstances may include the following non-exhaustive reasons:

- Pregnancy (further details on management of participants who become pregnant are provided in section 8.2).
- If a medication is started that would affect the study results, for example, intercurrent use of antibiotics.
- An adverse event which requires discontinuation of the study vaccinations or results in an inability to continue to comply with study procedures.
- Ineligibility (either arising during the study or in the form of new information not declared or detected at screening).

Withdrawal from the study will not result in exclusion of existing data generated by the participant from analysis. If the participant withdraws after Spn inoculation, they will be advised to complete the prescribed course of antibiotics after withdrawal. Participants can request that their samples are destroyed at any point during or after the study (although data that has already been generated from samples that have been analysed up to that point will be retained). The reason for withdrawal, if given, will be recorded in the eCRF.

- If the participant is withdrawn due to an adverse event, the Investigator may arrange for follow-up visits or telephone calls until the adverse event has resolved or stabilised.

9.11 Definition of End of Study

The clinical phase of the study ends when the last participant completes their last visit. Recruitment and follow up of participants from initial recruitment activities until last participant finishes their last study visit. GP and participant letters will be sent when a cohort of participants complete the clinical phase. The end of the study will be complete when all assays providing data for primary and secondary endpoints have been completed.

10. SAFETY REPORTING

10.1 Definitions

Our safety reporting terms and definitions are described in Table 8 below.

Term	Definition
**Adverse Event (AE)**	Any untoward medical occurrence in a participant to whom an inoculum has been administered, including occurrences, which are not necessarily caused by or related to that product.
**Adverse Event of Special Interest (AESI)**	An adverse event of special interest (serious or non-serious) is one of scientific and medical concern specific to the inoculation, for which ongoing monitoring and rapid communication by the investigator to the Sponsor can be appropriate. Such an event might warrant further investigation in order to characterise and understand it. Depending on the nature of the event, rapid

	communication by the study Sponsor to other parties (e.g., regulators, DSMC) might also be warranted.
**Serious Adverse Event (SAE)**	A serious adverse event is any untoward medical occurrence that:<br>• Results in death<br>• Is life-threatening<br>• Requires inpatient hospitalisation or prolongation of existing hospitalisation<br>• Results in persistent or significant disability/incapacity<br>• Consists of a congenital anomaly or birth defect<br>Other 'important medical events' may also be considered serious if they jeopardise the participant or require an intervention to prevent one of the above consequences.<br>NOTE: The term "life-threatening" in the definition of "serious" refers to an event in which the participant was at risk of death at the time of the event; it does not refer to an event which hypothetically might have caused death if it were more severe.

Table 8: Safety reporting terms. NB: to avoid confusion or misunderstanding of the difference between the terms "serious" and "severe", the following note of clarification is provided: "Severe" is often used to describe intensity of a specific event, which may be of relatively minor medical significance. "Serious" is the regulatory definition supplied above.

10.2 Grading

The labelling of an AE will be defined by the severity threshold described below in Table 9.

Severity grading criteria for local and systemic AEs. NB: A&E assessment in itself does not constitute a SAE. Refer to section 12.1 for SAE definition

**GRADE 0**	None
**GRADE 1**	Mild: Transient or mild discomfort (< 48 hours); No interference with activity; No medical intervention/therapy required
**GRADE 2**	Moderate: Mild to moderate limitation in activity – some assistance may be needed; no or minimal medical intervention/therapy required
**GRADE 3**	Severe: Marked limitation in activity, some assistance usually required; medical intervention/therapy required.

Table 9: Grading of adverse events

10.3 Causality

The relationship of each adverse event to inoculation must be determined by a medically qualified individual within the site study team according to the following definitions:

Not related	• No temporal relationship to LAIV or *S. pneumoniae* inoculation ***and***<br>• Alternative aetiology (clinical, environmental or other intervention), ***and***

	• Does not follow pattern of recognised response to LAIV or *S. pneumoniae* inoculation or other study procedure.
Possible	• Reasonable temporal relationship to LAIV or *S. pneumoniae* inoculation, ***or***<br>• Event not readily explained by alternative aetiology (clinical, environmental or other interventions), ***or***<br>• Similar pattern of response to that seen to LAIV or *S. pneumoniae*.
Probable	• Reasonable temporal relationship to LAIV or *S. pneumoniae* inoculation or other study procedure, ***and***<br>• Event not readily produced by alternative aetiology (clinical, environment, or other interventions), ***or***<br>• Known pattern of response with LAIV or *S. pneumoniae* or other study procedure.
Definite	• Reasonable temporal relationship to LAIV or *S. pneumoniae* inoculation or other study procedure, ***and***<br>• Event not readily produced by alternative aetiology (clinical, environment, or other interventions), ***and***<br>• Known pattern of response to LAIV or *S. pneumoniae or other study procedure*.

Table 10: Assessment of causality

10.4 Procedure for collecting and recording of adverse events

We will record solicited AEs occurring from Day –3 to Day 17 in group A and D0 to D20 in group B that are observed by the investigator or reported by the participant. Therefore, the diary is collected for a total of 21 days. Severity gradings will be in accordance with table 9. All SAEs will be recorded from time of enrolment. Participants will also be given the opportunity to report unsolicited AEs in an additional section of the e-diary.

AEs will be recorded in either:

- The e-diary (entry by participant)
- The eCRF (entry by study team)

Serious adverse events will be collected from enrolment until the participant completes the study.

All AEs that result in a participant's withdrawal from the study will, subject to participant consent, be followed up, where possible until a satisfactory resolution occurs, or until a non-study related causality is assigned.

AEs will be recorded using the following guidance:

- Pre-existing medical conditions (present before the study start) are considered medical history and should not be recorded as AEs. However, if the participant experiences a worsening or complication of such a condition, the worsening or complication should be recorded as an AE. Investigators should ensure that the AE term recorded captures the change in the condition (e.g., "worsening of")
- Each AE should be recorded to represent a single diagnosis. Accompanying signs or symptoms (including abnormal laboratory values) should not be recorded as additional AEs.

- Changes in laboratory values are only considered to be AEs if they are judged to be clinically significant, for example, if some action or intervention is required. If abnormal laboratory values are the result of pathology for which there is an overall diagnosis, the diagnosis only should be reported as one AE.

The following information will be recorded in the CRF:

- Description of the AE.
- The date of onset and end date.
- Severity of AE
- Assessment of relatedness to study procedure(s) (as judged by a medically qualified investigator).
- Action taken.

It will be left to the investigator's clinical judgment whether an AE is of sufficient severity to require the participant's removal from study. A participant may also voluntarily withdraw from the study due to what he or she perceives as an intolerable AE. If either of these occurs, the participant should undergo an end of study assessment and be given appropriate medical care (e.g. referral to their GP). If required, the investigator can refer the participant directly to hospital if the AE warrants it. NHS indemnity operates in respect of the clinical treatment provided to participants who may become unwell.

10.4.1 E-diary AEs

Solicited adverse events will be recorded by the participant in an electronic diary graded by the participant alone. Participants will be asked to complete an electronic diary during their first inoculation visit until 14 days after the last inoculation visit (total 21 days). Causality will be assigned by a member of the clinical team.

Solicited adverse events will be reviewed daily during the periods of recording as detailed above by the clinical study team. If the clinical team have concerns about the severity or frequency of an event, or a diary is not completed, this will be followed up with the participant by phone or at a scheduled visit. All ≥ grade 3 solicited adverse events recorded in the diary will be followed up with the participant by the clinical team in order to monitor for possible stopping rules. Participants will have access to the study team 24 hours a day via the study mobile number, should they have concerns.

10.4.2 Unsolicited adverse events

These may be recorded by the participant in an electronic diary for the same period as specified above. Unsolicited adverse events will be reviewed at clinic visits. If clarification of any event is required, then the study nurse or doctor will seek this from the participant during a clinical visit or by telephone call. Unsolicited adverse events recorded in the e-diary will be severity graded by the participant. Causality will be assigned as for solicited AEs.

In-person clinical reviews may be arranged at the investigator's discretion if there is sufficient clinical concern. This may be at the study site or during a home visit.

10.4.3 Vital sign AEs

At all visits vital signs are taken. These will be recorded directly into the eCRF at the time of review and severity grading will be automatically assigned as per Appendix B. Where a moderate or severe AE is identified a clinician should review the participant in clinic and document the clinical assessment carried

out. Changes in vital signs that are deemed clinically significant by a PI-delegated clinician will be causality assessed.

10.4.4 Laboratory AEs

Results of safety blood laboratory tests will be recorded onto a results eCRF and automatically graded as per Appendix C. If a test is deemed clinically significant, it may be repeated, to ensure it is not a single occurrence. If a test remains clinically significant, it will be recorded as an AE and the participant will be informed and medical care arranged as appropriate and with the permission of the participant. Laboratory results can be out of normal range for a number of reasons other than physiological disturbance (e.g. hot weather, delayed transit to processing laboratory).

10.5 Recording and reporting of serious adverse events (SAEs)

All SAEs will be reported on the SAE form (paper or electronic) to the CI (as delegated by the Sponsor) immediately (or within 24 hours at the latest) of the site study team becoming aware of the event. SAE's will be reported to the DSMC, CI (as delegated by the Sponsor) and the funder, the European Union and the United Kingdom (UK) Research and Innovation Horizon Programme. SAEs will be monitored and reported until the end of the participants last study visit or until resolution/stabilisation. Additional information received for a case (follow-up or corrections to the original case) need to be detailed on an SAE update form and reported as above. The DSMC will perform an independent review of the SAEs and request any further information required. Documentation of any review will be kept in the site file.

A serious adverse event (SAE) occurring to a participant should be reported to the REC that gave a favourable opinion of the study where in the opinion of the Chief Investigator the event was 'related' (resulted from administration of any of the research procedures) and 'unexpected' in relation to those procedures. Reports of related and unexpected SAEs should be submitted within 15 working days of the Chief Investigator becoming aware of the event, using the HRA report of serious adverse event form (see HRA website).

Serious adverse reactions/events (SARs) will be reported from the time of enrolment until the completion of the study.

Fatal or life threatening SUSARs must be reported within 7 days and all other SUSARs within 15 days. Any additional relevant information should be sent within 8 days of the report. All SUSARs will be reported to the Chief Investigator, Sponsor, DSMC and relevant Research Ethics Committee. The CI or delegate will also inform all Investigators concerned of relevant information about SUSARs that could adversely affect the safety of participants.

10.6 Adverse Events of Special Interest (AESI)

An adverse event of special interest is one of scientific and medical concern specific to a product or study/trial, for which ongoing monitoring and rapid communication by the investigator to the safety committee or Sponsor may be appropriate.

Due to the additional study procedures the following events will be considered AESIs.

- Invasive pneumococcal disease
- Pneumococcal pneumonia, otitis media, conjunctivitis, or pneumococcal meningitis

- Microbiologically confirmed transmission of Spn to a household contact
- Prolonged carrier state in a participant
- AEs requiring a physician visit or Emergency Department visit which, in the opinion of the study staff, are related to LAIV or the challenge with Spn.

11. STATISTICS AND ANALYSIS

11.1 Statistical Analysis Plan (SAP)

The statistical aspects of the study are summarised here with details fully described in a statistical analysis plan. The SAP will be finalised before any analysis takes place.

Statistical Analysis will be performed by statisticians within the Oxford vaccine group. The per-protocol population will be used for the primary and secondary analysis. A detailed statistical analysis plan (SAP) will be signed off before any formal analysis of the data.

11.2 Description of the Statistical Methods

Data Summaries

Continuous variables such as densities of pneumococcal colonisation, LAIV viral load and AUC of density over time will be summarised according to number of subjects with non- missing data (n), mean, standard deviation (SD), median, minimum, and maximum.

Categorical variables such as AEs and SAEs will be summarised according to the absolute frequency and percentage of subjects (%) in each category level. The denominator for the percentages is the number of subjects challenged with spn6B with data available, unless noted otherwise.

Analysis of primary outcome

Safety data and symptoms will be summarised and the number of events compared between study participants who received LAIV vaccination first followed by spn6B challenge and those who were challenged with Spn6B first followed by LAIV vaccination. Reference values will be derived from a previous study of older adults who were challenged with Spn6B only.

Analysis of secondary outcomes

The immune responses data are expected to be highly skewed, and the data will be log-transformed prior to analysis. The geometric mean concentration (GMC) and associated 95% confidence interval (CI) will be summarised by computing the anti-log of the mean of the log-transformed data. Data will be summarised by study arms at different time points.

Analysis of exploratory outcomes

The analysis of all the other secondary outcomes will be included in the SAP.

11.3 Sample Size Determination

In Group A we will randomise up to 60 healthy elderly subjects aged 55-80 years over 2 winter periods. Forty (n=40) participants will complete both arms, allowing for 20% dropout/screen failure and natural carriage. We may stop recruitment once we have complete datasets for 40 participants. Volunteers will be randomised into two arms that reflects alternative scenarios: 1) colonisation with pneumococcus followed by LAIV 3 days later (n=20) and 2), and LAIV followed by pneumococcus challenge 3 days later (n=20).

Group B will involve giving LAIV only to younger (n=7-10) and older adults (n=7-10). We will recruit up to 20 study participants in total. This sample size is based on feasibility assessment and sufficient numbers to identify gene signature in nasal and blood samples. Live attenuated flu vaccines have a very short shelf-life so to allow us to run the study throughout the winter period and account for antigen variation in the vaccine, randomisation will ensure that that both arms in Group A are spread evenly over the two years.

11.4 Populations for analysis of primary and secondary endpoints

The per-protocol population will be used for evaluation of primary and secondary outcomes. For primary outcome evaluation, the per-protocol population is defined as study participants who:

1. Have consented to the study and met all the inclusion and exclusion criteria;
2. Have not had prohibited medications during the study

   and for Group A:

   a. Have been successfully vaccinated with LAIV and challenged with Spn6B;
   b. Have had at least one nasal wash datapoint post second intervention (whether LAIV or pneumococcal challenge)

   for Group B:

   a. Have been successfully vaccinated with LAIV only

Exploratory endpoints will be analysed in the following populations:

- Participants who were vaccinated with LAIV and challenged with Spn6B and provided at least one post-challenge nasal sample.
- Participants who were vaccinated with LAIV alone and provided at least one post-vaccination nasal sample.
- For some of the analysis, such as gene expression, a subset of participants will be selected for analysis based on clinical outcomes and availability of samples for desired timepoints.

11.5 Decision points

An interim analysis is not scheduled; however, can be requested at the discretion of the principal investigator should any concerns arise. Decision points to pause or terminate the study will be made based on participant safety data if any concerns emerge.

11.6 Stopping rules

Stopping rules for individual participants will apply, which may result in halting progress to challenge.

- Solicited adverse events: the participant develops a grade 3 systemic solicited AE considered possibly, probably or definitely related within 2 days after challenge (day of challenge and one subsequent day) which persists for 48 hours or is deemed severe by clinician assessment
- Unsolicited adverse events: the participant has a grade 3 adverse event, considered possibly, probably or definitely related to challenge which persists for 48 hours or is deemed severe by clinician assessment or has a SAE considered possibly, probably or definitely related to challenge.

The SMG may make the decision to terminate the study early in the event of serious safety concerns raised by the DSMC.

The CI, with the DSMC will have the right to terminate the study at any time on grounds of participant safety. If the study is prematurely terminated the clinical study team will promptly inform the participants and will ensure appropriate therapy and follow-up.

If the study is terminated, the Sponsor, Study sites, Oxford University Hospitals NHS Foundation Trust (OUHFT) and relevant Ethics Committee will be notified within 15 days of this occurring.

11.7 The Level of Statistical Significance

Statistical significance will be assumed if $p < 0.05$ for two sided comparisons for primary and secondary outcome measures after appropriate adjustment if multiple tests are used.

11.8 Procedure for Accounting for Missing, Unused, and Spurious Data.

Reasons for missing data (including withdrawal of consent, loss to follow-up, removal from study due to serious side effects, death, or inability to obtain any laboratory results) will be indicated, but missing data will not be imputed. The quantity of missing data for the vaccine and placebo groups and the appertaining demographic characteristics will be compared. There will be an intention to publish all collected data, or at least open clarification about which additional variables have been measured if reporting is ultimately selective, so that readers can self-determine the possible impact of "data dredging", i.e. selective reporting of seemingly interesting results.

11.9 Procedures for Reporting any Deviation(s) from the Original Statistical Plan

A final statistical analysis plan (SAP) will be signed off before the final database lock. Any additional analysis or deviations from the SAP will be documented in the final analysis report and updated according to the statistical standard operating procedure.

12. DATA MANAGEMENT

The data management aspects of the study are summarised here, with details fully described in the Data Management Plan.

Each study participant will have a unique participant ID which will be allocated at the time of the screening visit. Names or identifying details are not included in any electronic file, containing study data. The exception to this is the electronic diaries, for which consent will be obtained to store the participant email address, which is necessary for the system to function. Only site research staff and sponsor data managers

have access to view the email address. Participant's personal information will be stored on a separate database not linked to the clinical database. Apart from clinical safety blood samples which are sent to local clinical laboratories and follow local sample labelling requirements, samples sent to laboratories for processing will be identified by study number only.

12.1 Source Data

Source documents are where data are first recorded, and from which participants' CRF data are obtained. These include, but are not limited to, hospital or GP records (from which medical history and previous and concurrent medication may be summarised into the CRF), laboratory and vaccination/pharmacy records, diaries, ultrasound images, and correspondence.

In this study, eCRF entries will be considered source data where it is the site of the original recording (e.g., there is no other written or electronic record of data). All documents will be stored safely in confidential conditions. On all study-specific documents, other than the signed consent and the participant contact sheet, the participant will be referred to by the study participant number, not by name.

12.2 Access to Data

Direct access will be granted to authorised representatives from (or appointed by) the Sponsor, host institution and the regulatory authorities to permit study-related monitoring, audits, and inspections.

12.3 Data Recording and Record Keeping

All study data will be recorded directly into eCRFs within Electronic Data Capture (EDC) system (e.g., REDCap, or similar), or onto a paper source document for later entry into the EDC system if direct entry is not available. Any additional information that needs recording but is not relevant for the eCRF (such as signed consent forms) will be recorded on a separate paper source document. All documents will be stored safely and securely in confidential conditions.

The EDC system (CRF data) uses a relational database (MySQL/ PostgreSQL) via a secure web interface with data checks applied during data entry to ensure data quality. The database includes a complete suite of features which are compliant with GCP, EU and UK regulations and Sponsor security policies, including a full audit trail, user-based privileges, and integration with the institutional LDAP server. The MySQL and PostgreSQL database and the webserver will both be housed on secure servers maintained by Oxford Vaccine Group IT personnel. The servers are in a physically secure location in Europe, and data are backed up on secure servers operated by the University of Oxford IT Services, physically located in Europe. Backups will be stored in accordance with the IT department schedule of daily, weekly, and monthly retained for one month, three months, and six months, respectively. Weekly backup tapes are stored offsite. The servers provide a stable, secure, well-maintained, and high-capacity data storage environment. REDCap is a widely used, powerful, reliable, well-supported system. Access to the study's database will be restricted to the members of the study team by username and password.

The study team will use names and contact details to contact participants about the research study, and make sure that relevant information about the study is recorded for their care, in relation to their health during the study and to oversee the quality of the study. At the completion of the study, unless participants consent otherwise (*e.g.,* requesting to be informed of other trials), participant's personal details will not

be used to contact them other than in exceptional circumstances concerning their safety. If consent is provided by participants to take part in another study carried out by the study site, personal information and medical information including blood test results may be accessed to avoid unnecessary repetition. If participants provide specific consent, we will use personal identifiable data to invite participants for future research.

Data collection and storage will be inspected throughout the study by the Oxford Vaccine Group and monitoring will be carried out by monitors with the Oxford Vaccine Group team.

13. QUALITY ASSURANCE PROCEDURES

Approved standard operating procedures (SOPs) will be used at all clinical and laboratory sites.

13.1 Risk assessment

The study will be conducted in accordance with the current approved protocol, GCP, relevant regulations and Standard Operating Procedures. A risk assessment and monitoring plan will be prepared before the study opens and will be reviewed as necessary over the course of the study to reflect significant changes to the protocol or outcomes of monitoring activities. Approved and relevant SOPs will be used at all clinical and laboratory sites.

**Screening**	Exclude adults with potential risk factors for invasive pneumococcal infection based on history, vital signs, clinical assessment and safety laboratory samples. Exclude adults who have contacts with risk factors for invasive or severe infection.
**Participant safety**	Safety guidance is presented to the participant during consent and on the days of inoculation. Participants are provided a safety information leaflet detailing:<br>• contact details for the research team available 24/7<br>• symptoms of infection<br>• advice to report early signs of infection or to seek urgent health care if concerned<br>• if/when to take the antibiotics provided<br>• to report adverse events including unrelated hospital admissions for the duration of the follow up period until their last visit<br>• how to maintain regular hand washing.<br><br>Close friend or family: participants are encouraged to inform a close contact that they are taking part in a study and a copy of the safety leaflet is provided for them including contact details for the research team. We advise participants that if they are unwell, they should contact the clinical research team and inform their identified close contact. The close contact's contact details will be provided to the study team should the site be unable to locate the participant. Attempts will be made to contact the close contact instead if there are concerns.
**Symptoms and access to healthcare**	**Urgent Care:** to avoid any delay in diagnosis, participants are advised to attend their usual health care facility (wearing a fluid resistant surgical mask) or dial 111 if seriously concerned about their health, as their condition may not necessarily be related to the inoculation. They will also be advised to inform the research team.

	**Daily checks:** following primary inoculation participants will complete an electronic or paper diary to report any symptoms for a total of 21 days. Thermometers are provided for daily temperature measurement.<br>**Symptoms:** will be monitored and recorded systematically at each visit by the clinical research team.<br>**Triggered clinician assessment:** participants with respiratory/ear symptoms, fever, and other symptoms potentially associated with pneumococcal infection may attend the clinic for a triggered assessment by the research nurse/doctor. They may be advised to seek another healthcare route if a visit with the research nurse/doctor is not possible, or the symptoms are severe. A research doctor is available for telephone advice 24/7 for participants.<br>**General Practitioner:** will be routinely notified of participants involvement in the study.
**Antibiotics**	**Sensitivity:** the bacterial inoculum will be tested to confirm sensitivity to the protocol antibiotics.<br>**Supply of antibiotics:** Oral Amoxicillin 500mg TDS for 3 days is provided to each participant to avoid delay in treatment if the participant has symptoms of potential pneumococcal infection (preferably taken upon discussion with the clinical team).<br>**Termination of colonisation:** participants who remain bacterial colonisation positive at their last nasal wash visit will be advised to take a 3-day course of amoxicillin 500mg TDS antibiotics to clear or reduce colonisation post inoculation.
**Monitoring colonisation**	**Nasal Wash:** results of colonisation are reported to the clinical team each follow-up visits following inoculation. Colonisation and safety data are communicated weekly for discussion by the SMG.
**Withdrawal**	If a participant or their close contacts develop potential risk factors for invasive bacterial, then they may be withdrawn from the study at any time and commence antibiotics to clear/reduce bacterial colonisation when required.
**Staff Safety**	Use of personal protective equipment, assessment by Occupational Health, management of high-risk staff members (e.g. pregnant or immunocompromised individuals)
**Safety monitoring**	**Study Oversight:** An established DSMC and SMG will review infection/colonisation rates and adverse events.

13.1.1. Risks related to FNA of lymph nodes

Expected adverse events following lymph node aspiration include sample site pain or tenderness. Haematoma is a rare risk, and minimal bleeding may occur after the aspiration but should resolve spontaneously. Participants at increased risk due to blood-thinning medication will be excluded. Bruising may occur but is expected to fade after 2 weeks. In a study lead by the chief investigator, common adverse events attributable to the FNA (tenderness/pain, bruising, swelling) were mild in nature and resolved within 5 days(Day et al., 2022). The chief investigator is conducting studies that included >390 US guided axillary lymph node FNA procedures (NCT03816137, ISRCTN13657999, ISRCTN12928349, ISRCTN11688703) with no serious adverse events related to the procedure.

Damage to the underlying/adjacent structures is an extremely rare risk effectively minimized by direct visualization under ultrasound guidance. In a different study conducted at the University of Oxford (VAC096), a pneumothorax occurred after an FNA procedure and was considered by the investigators of that study to be related to study procedures.

Participants will be provided with information regarding both these expected and rare adverse events in the participant information sheet and adverse events will be monitored and reported.

13.1.2. Risks related to Live Attenuated Influenza Vaccine

LAIV is licensed for use in children and young people up until 18 years of age. The vaccine is well tested and in regular use, although we are using it outside of market authorisation for this study. The most common adverse reaction following administration is nasal congestion/rhinorrhoea as listed in the SmPC of the vaccine.

The following side effects were seen in clinical trials in children and young people:

- Very common (≥1/10): decreased appetite, nasal congestion/rhinorrhoea, malaise
- Common (≥1/100 to ≥1/10): headache, myalgia, pyrexia
- Uncommon (≥1/1,000 to ≥1/100): hypersensitivity reactions (including facial oedema, urticaria and very rare anaphylactic reactions), epistaxis, rash

13.1.3. Risks related to Pneumococcal inoculation

Pneumococcus is responsible for infections including otitis media (OM), sinusitis, pneumonia, bacteraemia and meningitis. The milder forms of infection (OM, sinusitis, conjunctivitis) are many times more common than the serious invasive forms of the disease. Due to inoculating participants with pneumococcus, there is a very low risk of OM, sinusitis, conjunctivitis, pneumonia, bacteraemia and meningitis. While the risk to individuals of developing any infection is very low (10% adults experience natural colonisation at any time and the incidence of invasive disease is 20/100,000 patient years), the study is designed to ensure that any risk is minimal.

This study can be safely run based on the following experience and provisions:

- The research team has 14 years of experience in human challenge studies, following very similar protocols and facing similar risks as previous studies.
- The selected pneumococcal serotype (6B) is fully antibiotic sensitive.
- Participant selection and exclusion criteria reduce the excess risk of invasive pneumococcal disease associated with comorbid conditions.
- Participant education regarding the risks of study participation, provision of a safety information leaflet, and close interaction with the study staff.
- Rigorous and frequent monitoring of development of symptoms and body temperature.
- Provision of standby antibiotics to reduce time to treatment, if it is required.
- Provision of and eye ointment on diagnosis in the event of conjunctivitis to reduce time to treatment, if it is required.
- 24-hour emergency telephone contact with researchers (including individual daily diary monitoring for the first 7 days following inoculation), to facilitate access to hospital and/or prompt treatment if required.

Within the safety information leaflet, participants will be warned to look out for specific symptoms relating to infections caused by *Streptococcus pneumoniae.* That includes: fever (>37.5 ˚C), shivering, headache, new rash, drowsiness, cough, shortness of breath, earache or symptoms of an eye infection.

We now have experience of inoculating and following over 2000 participants using several serotypes, in different age cohorts and at a range of doses (20,000-320,000 CFU/100μL), with participants being experimentally colonised, naturally colonised and not colonised during our studies. We have had two separate SAEs reported during these challenge studies, both classified as unrelated to the study protocols and study conduct.

In the event that symptoms occur during the inoculation or follow up period, volunteers will be contacted on the day that the symptoms are reported and the clinical team will advise participants to commence amoxicillin based on a pre-defined algorithm (available in the EHPC SOPs) and irrespective of the colonisation status at that point. Participants may be reviewed in study clinic or asked, at investigator discretion, to attend an NHS healthcare treatment facility directly.

In the event of an unscheduled visit for clinical assessment, safety samples (blood samples and swabs) can be taken at investigator discretion.

13.2 Study monitoring

Monitoring will be performed according to the principles of Good Clinical Practice (GCP) by parties appointed by OVG. The investigator sites will provide direct access to all study related source data/documents and reports for the purpose of monitoring and auditing by the Sponsor and inspection by local and regulatory authorities.

13.3 Study Committees

Study Management Group (SMG):

Includes scientists, health professionals and investigators who provide ongoing management of the study. They conduct the study and review recruitment, safety and colonisation report weekly.

Data and Safety Monitoring Committee (DSMC):

The DSMC safeguards and monitors the interests of the study participants by reviewing the protocol according to the DSMC charter, assessing the safety of interventions and colonisation rate data throughout the study. They periodically review safety data to determine patterns and trends of events, or to identify safety issues, which would not be apparent on an individual case basis. They may review data in the interest of safety.

- To independently review AEs, SAEs and AESIs regardless of relatedness to any of the study procedures throughout the study.
- To formally review the safety profile and colonisation rate of the inoculum during the pilot phase before progression to the second phase and at the end of the study.
- To perform unscheduled reviews on request of the study team at a demand and frequency determined by the severity of reported adverse events.

Interim data will be provided if at any time the SMG have any concerns regarding the safety of a participant or the general public. The DSMC will advise the study investigators on whether there are any ethical or safety reasons why the study should be changed or not continue. The DSMC will meet as per the terms of reference.

The Chair of the DSMC will also be contacted for advice where the CI feels independent advice or review is required. A further DSMC meeting will be held at the conclusion of the study,

14. PROTOCOL DEVIATIONS

A study related deviation is a departure from the ethically approved study protocol or other study document or process (e.g. consent process or administration of study intervention) or from Good Clinical Practice (GCP) or any applicable regulatory requirements. Any deviations from the protocol will be documented in a protocol deviation form and filed in the study master file.

15. SERIOUS BREACHES

A "serious breach" is a breach of the protocol or of the conditions or principles of Good Clinical Practice which is likely to affect to a significant degree –

(a) the safety or physical or mental integrity of the study subjects; or

(b) the scientific value of the research.

In the event that a serious breach is suspected the Sponsor must be contacted within 1 working day. In collaboration with the C.I., the serious breach will be reviewed by the Sponsor and, if appropriate, the Sponsor will report it to the approving REC committee and the relevant NHS host organisation within seven calendar days.

16. ETHICAL AND REGULATORY CONSIDERATIONS

16.1 Declaration of Helsinki

The Investigator will ensure that the study is conducted in accordance with the principles of the Declaration of Helsinki.

16.2 Guideline for Good Clinical Practice

The Investigator will ensure that this study is conducted in accordance with relevant regulations and with Good Clinical Practice

16.3 Approvals

Following Sponsor approval, the protocol, informed consent form, participant information sheet, and required material will be submitted to an appropriate Research Ethics Committee (REC), regulatory authorities, and host institutions for written approval.

The Investigator will submit and, where necessary, obtain approval from the above parties for all substantial amendments to the original approved documents.

16.4 Other Ethical Considerations

When performing an ultrasound scan for the FNA biopsy, in the unlikely event of seeing any structural abnormalities, the scan will be checked by a clinical specialist. This only applies to the subset of participants that are invited to attend for FNA biopsy. If the specialist feels that the abnormality was medically important, they will discuss the implications with the participant and arrange for further investigations as necessary. Participants will not be informed unless the doctor considers the finding has clear implications for their current or future health. It is important to note that scans are not carried out for diagnostic purposes, and therefore the scans are not a substitute for a clinical appointment. Rather, the scans are intended for research purposes only.

16.5 Reporting

The CI shall submit once a year throughout the study, or on request, an Annual Progress report to the host organisation, Sponsor and funder (where required). In addition, an End of Study notification and final report will be submitted to the same parties.

16.6 Transparency in Research

Prior to the recruitment of the first participant, the study will have been registered on a publicly accessible database. Results will be uploaded to ISRCTN Database/CT.gov within 12 months of the end of study (as declared by the CI or their delegate). Where the study has been registered on multiple public platforms, the study information will be kept up to date during the study, and the CI or their delegate will upload results to all those public registries within 12 months of the end of the study declaration.

16.7 Participant Confidentiality

With the exception of clinical samples, which are sent to local clinical laboratories and follow local sample labelling requirements (typically including the participant’s medical record number (MRN), NHS number, name, sex and date of birth), samples sent to other laboratories for processing will be identified by study number and participant number only.

All documents will be stored securely and only accessible by study staff and authorised personnel. The study will comply with UK General Data Protection Regulation (GDPR) and Data Protection Act 2018, which requires data to be anonymised as soon as it is practical to do so.

16.8 The Over-volunteering Prevention System

The Over-volunteering Prevention System (TOPS) is a database to guard against the potential for harm that can result from excessive volunteering in clinical trials involving IMPs or blood donations. Participants will be registered for TOPS and checked for conflicts at screening using their national insurance number or passport number.

The system will be updated in the event of the participant being withdrawn or excluded. Alternatively, TOPS will be updated on the participant’s last visit.

16.9 Expenses and Benefits

It is not intended that financial factors influence an individual's decision to participate in this study. The payments will reflect remuneration and not financial coercion. We compensate participants for time,

travel, inconvenience and sample collection. The sums offered are consistent with remuneration in other similar local and national studies and are detailed below:

Participants will be reimbursed £110 for their screening and inoculation visits. For each of the follow up visits, participants will be reimbursed £90. Upon full completion of the diary card, participants will be reimbursed a further £30. £20 will be reimbursed for each set of home samples taken by the participant.

Each participant in Group A can therefore receive a maximum of £1060 and each participant in Group B a maximum of £700 for the scheduled study visits/samples. They may receive further reimbursements, based on whether unscheduled/FNA/Nasal biopsy visits were required and how many occurred. FNA visits will be reimbursed at an additional £150. Nasal biopsy visits will be reimbursed at an additional £160.

Remuneration is on a pro rata basis. Should a participant not complete all visits and/or study requirements, or if a participant withdraws from the study early, they will be remunerated for the visits they attended and samples which were taken up until the time they withdrew. Additional reimbursement for unscheduled visits at £90 per visit will be provided. This will not be given unless an unscheduled visit occurs.

The total amount of compensation for an individual participant will depend on the actual number of visits attended and whether any repeat or additional visits were necessary.

17. FINANCE AND INSURANCE

17.1 Funding

Funding for the study has been provided by Horizon Europe Guarantee Extension, UKRI Reference number: 10077113.

17.2 Insurance

The University has a specialist insurance policy in place which would operate in the event of any participant suffering harm as a result of their involvement in the research (Newline Underwriting Management Ltd, at Lloyd’s of London). NHS indemnity operates in respect of the clinical treatment provided.

17.3 Contractual arrangements

Appropriate contractual arrangements will be put in place with all third parties.

18. PUBLICATION POLICY

The Investigators will be involved in reviewing drafts of the manuscripts, abstracts, press releases and any other publications arising from the study. The drafts may also be reviewed by the Nosevac consortium prior to dissemination.

All communication or publications concerning the project, including at a conference or seminar, shall acknowledge the parties involved, and contribution by UK Research and Innovation (UKRI) under the

Horizon Europe Guarantee Extension. Authorship will be determined in accordance with the ICMJE guidelines and other contributors will be acknowledged.

19. DEVELOPMENT OF A NEW PRODUCT/ PROCESS OR THE GENERATION OF INTELLECTUAL PROPERTY

Ownership of IP generated by employees of the University vests in the University. The University will ensure appropriate arrangements are in place as regards any new IP arising from the study.

20. ARCHIVING

Study data may be stored electronically on a secure server by the University IT team, and paper notes will be kept in a secure location at each study site or as outlined in local SOP's. We will store the research data and any research documents with personal information, such as consent forms, securely for up to 25 years after the end of the study, or as per national regulatory requirements. Anonymised research data may be kept indefinitely

Participants' bank details will be stored for a minimum of 7 years in line with the Oxford University financial policy. Volunteers who complete online screening only (before informed consent) will not have data kept beyond the end of the study. General archiving procedures will be conducted in compliance to SOP OVC020 Archiving.

21. REFERENCES

Adler, H., German, E.L., Mitsi, E., Nikolaou, E., Pojar, S., Hales, C., Robinson, R., Connor, V., Hill, H., Hyder-Wright, A.D., Lazarova, L., Lowe, C., Smith, E.L., Wheeler, I., Zaidi, S.R., Jochems, S.P., Loukov, D., Reiné, J., Solórzano-Gonzalez, C., de Gorguette d'Argoeuves, P., Jones, T., Goldblatt, D., Chen, T., Aston, S.J., French, N., Collins, A.M., Gordon, S.B., Ferreira, D.M., Rylance, J., 2021. Experimental human pneumococcal colonization in older adults is feasible and safe, not immunogenic. Am J Respir Crit Care Med 203, 604–613. https://doi.org/10.1164/rccm.202004-1483OC

Almeida, S.T., Nunes, S., Santos Paulo, A.C., Valadares, I., Martins, S., Breia, F., Brito-Avô, A., Morais, A., De Lencastre, H., Sá-Leão, R., 2014. Low prevalence of pneumococcal carriage and high serotype and genotype diversity among adults over 60 years of age living in Portugal. PLoS One 9. https://doi.org/10.1371/journal.pone.0090974

Bewick, T., Sheppard, C., Greenwood, S., Slack, M., Trotter, C., George, R., Lim, W.S., 2012. Serotype prevalence in adults hospitalised with pneumococcal non-invasive community-acquired pneumonia. Thorax 67, 540–545. https://doi.org/10.1136/thoraxjnl-2011-201092

Bleve, A., Motta, F., Durante, B., Pandolfo, C., Selmi, C., Sica, A., 2023. Immunosenescence, Inflammaging, and Frailty: Role of Myeloid Cells in Age-Related Diseases. Clin Rev Allergy Immunol. https://doi.org/10.1007/s12016-021-08909-7

Carniel, B.F., Marcon, F., Rylance, J., German, E.L., Zaidi, S., Reiné, J., Negera, E., Nikolaou, E., Pojar, S., Solórzano, C., Collins, A.M., Connor, V., Bogaert, D., Gordon, S.B., Nakaya, H.I., Ferreira, D.M., Jochems, S.P., Mitsi, E., 2021. Pneumococcal colonization impairs mucosal immune responses to live attenuated influenza vaccine 6. https://doi.org/10.1172/jci

Chien, Y.-W., Klugman, K.P., Morens, D.M., 2009. Bacterial pathogens and death during the 1918 influenza pandemic. New England Journal of Medicine 361, 2582–2583.

Day, S., Kaur, C., Cheeseman, H.M., de Groot, E., McFarlane, L.R., Tanaka, M., Coelho, S., Cole, T., Lemm, N.M., Lim, A., Sanders, R.W., Asquith, B., Shattock, R.J., Pollock, K.M., 2022. Comparison of blood and lymph node cells after intramuscular injection with HIV envelope immunogens. Front Immunol 13. https://doi.org/10.3389/fimmu.2022.991509

Ferreira, D.M., Neill, D.R., Bangert, M., Gritzfeld, J.F., Green, N., Wright, A.K.A., Pennington, S.H., Moreno, L.B., Moreno, A.T., Miyaji, E.N., Wright, A.D., Collins, A.M., Goldblatt, D., Kadioglu, A., Gordon, S.B., 2013. Controlled human infection and rechallenge with Streptococcus pneumoniae reveals the protective efficacy of carriage in healthy adults. Am J Respir Crit Care Med 187, 855–864. https://doi.org/10.1164/rccm.201212-2277OC

Frasca, D., DIaz, A., Romero, M., Garcia, D., Blomberg, B.B., 2020. B Cell Immunosenescence. Annu Rev Cell Dev Biol. https://doi.org/10.1146/annurev-cellbio-011620-034148

German, E.L., Solórzano, C., Sunny, S., Dunne, F., Gritzfeld, J.F., Mitsi, E., Nikolaou, E., Hyder-Wright, A.D., Collins, A.M., Gordon, S.B., Ferreira, D.M., 2019. Protective effect of PCV vaccine against experimental pneumococcal challenge in adults is primarily mediated by controlling colonisation density. Vaccine 37, 3953–3956. https://doi.org/10.1016/j.vaccine.2019.05.080

Hammitt, L.L., Bruden, D.L., Butler, J.C., Baggett, H.C., Hurlburt, D.A., Reasonover, A., Hennessy, T.W., 2006. Indirect Effect of Conjugate Vaccine on Adult Carriage of Streptococcus pneumoniae: An Explanation of Trends in Invasive Pneumococcal Disease, Indirect Effect of PCV7 on Adult Carriage • JID.

Jackson, G.G., Dowling, H.F., Spiesman, I.G., Boand, A. V, 1958. Transmission of the Common Cold to Volunteers Under Controlled Conditions: I. The Common Cold as a Clinical Entity. AMA Arch Intern Med 101, 267–278. https://doi.org/10.1001/archinte.1958.00260140099015

Jochems, S.P., De Ruiter, K., Solórzano, C., Voskamp, A., Mitsi, E., Nikolaou, E., Carniel, B.F., Pojar, S., German, E.L., Reiné, J., Soares-Schanoski, A., Hill, H., Robinson, R., Hyder-Wright, A.D., Weight, C.M., Durrenberger, P.F., Heyderman, R.S., Gordon, S.B., Smits, H.H., Urban, B.C., Rylance, J., Collins, A.M., Wilkie, M.D., Lazarova, L., Leong, S.C., Yazdanbakhsh, M., Ferreira, D.M., 2019. Innate and adaptive nasal mucosal immune responses following experimental human pneumococcal colonization. Journal of Clinical Investigation 129, 4523–4538. https://doi.org/10.1172/JCI128865

Jochems, S.P., Marcon, F., Carniel, B.F., Holloway, M., Mitsi, E., Smith, E., Gritzfeld, J.F., Solórzano, C., Reiné, J., Pojar, S., Nikolaou, E., German, E.L., Hyder-Wright, A., Hill, H., Hales, C., de Steenhuijsen Piters,

W.A.A., Bogaert, D., Adler, H., Zaidi, S., Connor, V., Gordon, S.B., Rylance, J., Nakaya, H.I., Ferreira, D.M., 2018. Inflammation induced by influenza virus impairs human innate immune control of pneumococcus. Nat Immunol 19, 1299–1308. https://doi.org/10.1038/s41590-018-0231-y

Karagiannis, T.T., Dowrey, T.W., Villacorta-Martin, C., Montano, M., Reed, E., Belkina, A.C., Andersen, S.L., Perls, T.T., Monti, S., Murphy, G.J., Sebastiani, P., 2023. Multi-modal profiling of peripheral blood cells across the human lifespan reveals distinct immune cell signatures of aging and longevity.

Lavelle, E.C., Ward, R.W., 2022. Mucosal vaccines — fortifying the frontiers. Nat Rev Immunol. https://doi.org/10.1038/s41577-021-00583-2

Lewnard, J.A., Bruxvoort, K.J., Fischer, H., Hong, V.X., Grant, L.R., Jódar, L., Gessner, B.D., Tartof, S.Y., 2022. Prevention of Coronavirus Disease 2019 Among Older Adults Receiving Pneumococcal Conjugate Vaccine Suggests Interactions Between Streptococcus pneumoniae and Severe Acute Respiratory Syndrome Coronavirus 2 in the Respiratory Tract. Journal of Infectious Diseases 225, 1710–1720. https://doi.org/10.1093/infdis/jiab128

Miller, E., Andrews, N.J., Waight, P.A., Slack, M.P.E., George, R.C., 2011. Herd immunity and serotype replacement 4 years after seven-valent pneumococcal conjugate vaccination in England and Wales: an observational cohort study. Lancet Infect Dis 11, 760–768. https://doi.org/10.1016/S1473-3099(11)70090-1

Mogilenko, D.A., Shchukina, I., Artyomov, M.N., 2022. Immune ageing at single-cell resolution. Nat Rev Immunol. https://doi.org/10.1038/s41577-021-00646-4

Naclerio, R.M., Meier, H.L., Kagey-Sobotka, A., Adkinson, N.F., Meyers, D.A., Norman, P.S., Lichtenstein, L.M., 1983. Mediator Release after Nasal Airway Challenge with Allergen. American Review of Respiratory Disease 128, 597–602. https://doi.org/10.1164/arrd.1983.128.4.597

National Institute for Health and Care Excellence. *When should I suspect type 2 diabetes in an adult?* May 2024. https://cks.nice.org.uk/topics/diabetes-type-2/diagnosis/diagnosis-in-adults/HbA1c of 48 mmol/mol (6.5%) or more

National Institute for Health and Care Excellence. *Type 1 diabetes in adults: diagnosis and management (NG17) 1.1 Diagnosis and early care plan.* Aug 2022. < https://www.nice.org.uk/guidance/ng17/chapter/Recommendations#diagnosis-and-early-care-plan>

Robinson, R.E., Myerscough, C., He, N., Hill, H., Shepherd, W.A., Gonzalez-Dias, P., Liatsikos, K., Latham, S., Fyles, F., Doherty, K., Hazenberg, P., Shiham, F., Mclenghan, D., Adler, H., Randles, V., Zaidi, S., Hyder-Wright, A., Mitsi, E., Burhan, H., Morton, B., Rylance, J., Lesosky, M., Gordon, S.B., Collins, A.M., Ferreira, D.M., 2023. Comprehensive review of safety in Experimental Human Pneumococcal Challenge. PLoS One 18. https://doi.org/10.1371/journal.pone.0284399

Rylance, J., de Steenhuijsen Piters, W.A., Mina, M.J., Bogaert, D., French, N., Ferreira, D.M., 2019. Two Randomized Trials of the Effect of Live Attenuated Influenza Vaccine on Pneumococcal Colonization. Am J Respir Crit Care Med 199, 1160–1163.

Thors, V., Christensen, H., Morales-Aza, B., Vipond, I., Muir, P., Finn, A., 2016. The effects of live attenuated influenza vaccine on nasopharyngeal bacteria in healthy 2 to 4 year olds: A randomized controlled trial. Am J Respir Crit Care Med 193, 1401–1409. https://doi.org/10.1164/rccm.201510-2000OC

Tsoumani, E., Carter, J.A., Salomonsson, S., Stephens, J.M., Bencina, G., 2023. Clinical, economic, and humanistic burden of community acquired pneumonia in Europe: a systematic literature review. Expert Rev Vaccines. https://doi.org/10.1080/14760584.2023.2261785

Urban, B.C., Gonçalves, A.N., Loukov, D., Passos, F.M., Reiné, J., Gonzalez-Dias, P., Solórzano-Gonzalez, C., Mitsi, E., Nikolaou, E., Collins, A.M., Adler, H., Rylance, J., Gordon, S.B., Jochems, S.P., Nakaya, H.I., Ferreira, D.M., Paulo, S., 2023. Cellular and Transcriptional Signature of the Nasal Mucosa is Associated with Susceptibility to Pneumococcal Carriage in Older Adults. medRxiv. https://doi.org/10.1101/2023.11.16.23298619

Weinberger, D.M., Givon-Lavi, N., Shemer-Avni, Y., Bar-Ziv, J., Alonso, W.J., Greenberg, D., Dagan, R., 2013. Influence of pneumococcal vaccines and respiratory syncytial virus on alveolar pneumonia, Israel. Emerg Infect Dis 19, 1084–1091. https://doi.org/10.3201/eid1907.121625

Weinberger, D.M., Klugman, K.P., Steiner, C.A., Simonsen, L., Viboud, C., 2015. Association between Respiratory Syncytial Virus Activity and Pneumococcal Disease in Infants: A Time Series Analysis of US Hospitalization Data. PLoS Med 12, 1–12. https://doi.org/10.1371/journal.pmed.1001776

Wright, A.K.A., Bangert, M., Gritzfeld, J.F., Ferreira, D.M., Jambo, K.C., Wright, A.D., Collins, A.M., Gordon, S.B., 2013. Experimental Human Pneumococcal Carriage Augments IL-17A-dependent T-cell Defence of the Lung. PLoS Pathog 9. https://doi.org/10.1371/journal.ppat.1003274

22. APPENDIX A: Upper and lower respiratory symptom score

Upper respiratory clinical symptom score scale

Symptom	Severity			
	0	1	2	3
Sneezing	No sneezing present	Present but no interference with activity	Some interference with activity	Significant; prevents daily activity
Headache	No headache present	Present but no interference with activity	Some interference with activity	Significant; any use of codeine phosphate or prevents daily activity
Malaise	No malaise	Present but no interference with activity	Some interference with activity	Significant; prevents daily activity
Fever / chills	No fever	Present but no interference with activity	Some interference with activity	Significant; prevents daily activity
Nasal discharge	No nasal discharge	Present but no interference with activity	Some interference with activity	Significant; prevents daily activity
Nasal obstruction	No nasal congestion present	Present but no interference with activity	Some interference with activity	Significant; prevents daily activity
Sore throat	No sore throat present	Present but no interference with activity	Some interference with activity	Significant; any use of codeine phosphate or prevents daily activity
Cough	No cough present	Present but no interference with activity	Some interference with activity	Significant; prevents daily activity

Table 11: Upper respiratory symptom score scale

Lower respiratory clinical symptom score scale

Symptom	Severity			
	0	1	2	3
Cough on waking	No cough present	Present but no interference with activity	Some interference with activity	Significant; prevents daily activity
Wheeze on waking	No wheeze present	Present but no interference with activity	Some interference with activity	Significant; prevents daily activity
Daytime cough	No cough present	Present but no interference with activity	Some interference with activity	Significant; prevents daily activity
Daytime wheeze	No wheeze present	Present but no interference with activity	Some interference with activity	Significant; prevents daily activity
Daytime shortness of breath	No shortness of breath present	Present but no interference with activity	Some interference with activity	Significant; prevents daily activity
Nocturnal cough, wheeze or shortness of breath	No shortness of breath present	Present but no interference with activity	Some interference with activity	Significant; prevents daily activity

Table 12: Lower respiratory symptom score scale

23. APPENDIX B: Grading the severity of visit-observed Adverse Events

Observation	Grade 1	Grade 2	Grade 3
Oral temperature (°C)	37.6 – 38.0	38.1 – 39.0	> 39.0
Tachycardia (beats/min)	101-115	116-130	>130
Bradycardia (beats/min)	50-54	45-49	<45
Systolic hyper-tension (mmHg)	141-150	151-155	>155
Diastolic hyper-tension (mmHg)	91-95	96-100	>100
Systolic hypo-tension (mmHg)	85-89	80-84	<80

Table 13: Grading the severity of the visit-observed AEs

24. APPENDIX C: Grading the severity of laboratory Adverse Events

Parameter	Grade 1	Grade 2	Grade 3
**Haemoglobin: decrease from baseline value (g/l)**	10 - 15	16-20	21-50
**White cell count: elevated (10^9/L)**	11.01 – 15.00	15.01 – 20.00	20.01 – 25.00
**White cell count: depressed (10^9/L)**	2.50 – 3.50	1.50 – 2.49	1.00 – 1.49
**Neutrophil count (10^9/L)**	1.5-2.0	1.0-1.4	0.5-0.9
**Platelets (10^9/L)**	125-140	100-124	25-99
**Sodium: hyponatraemia (mmol/L)**	132–134	130–131	125–129
**Sodium: hypernatremia (mmol/L)**	146	147	148–150
**Potassium: hyperkalaemia (mmol/L)**	5.4 – 5.5	5.6 – 5.7	5.8 – 5.9
**Potassium: hypokalaemia (mmol/L)**	3.3–3.4	3.1–3.2	3.0
**Urea (mmol/L)**	8.2–8.9	9.0–11	>11

Table 14: Grading the severity of laboratory AEs

***AEs for laboratory results will be raised if deemed clinically significant**

25. APPENDIX D: Amendment History

Amendment No.	Protocol Version No.	Author(s) of changes	Details of Changes made
1	V2.1	Oisin Hennigan, Ella Morey, Britta Urban	Removal of Maheshi Ramasamy as an investigator throughout<br><br>Clarification of laboratory investigations in secondary and exploratory objectives and throughout<br><br>Removal of allowing combining screening and Day – 7/Baseline assessment visits throughout

			Clarified enrolment definition as occurring during baseline assessment visit immediately prior to collection of research samples throughout<br><br>Section 7: Infographic updated to include saliva sampling type, Figure 1 and 2<br><br>Section 8.1: Clarified pneumococcal vaccination exclusion so it matches what is outlined in section 8.3<br><br>Section 8.3: Clarified exclusion criteria for healthcare workers was only for those with direct caring responsibilities for those at increased risk of pneumococcal disease for group A only<br><br>Section 9.2: Clarified ECG and Next of Kin details required for Group A participants only<br><br>Section 9.4: Clarified may recruit more participants depending on availability for either season<br><br>Section 9.6.3: Edited visit table to reflect screening and baseline assessment visits occurring separately as outlined above<br><br>Section 9.7.1.2: Clarified that LAIV formulation changes annually and that the seasonal choice of vaccine will be based on UK influenza immunisation guidelines<br><br>Section 9.7.2: Changed number of passes for FNA to 3-6 to align with current practice by radiology team<br><br>Section 9.11: Added that GP and participant letters to be sent when a cohort of participants complete the clinical phase<br><br>Section 13.1.1: Risks related to FNA of lymph nodes updated to reflect FNA-related SAE in a different study conducted at the University of Oxford and provided the safety profile from ongoing studies conducted by the PI.<br><br>Section 16.7: Clarified local sample labelling requirements would include participants MRN, NHS number etc
2	V3.0	Oisin Hennigan, Britta Urban, Bhumika Patel, Carla Solorzano-Gonzalez Thejaswini Madupuri	Section 7:<br>-Updated study visit schedule chart to include optional nasal biopsy visit<br><br>Section 8.3 and 8.4:<br>-Exclusion and Temporary Exclusion criteria updated based on nasal biopsy procedure

			Section 9.3 updated to include a separate consent form for those participants wishing to get the optional nasal biopsy procedure<br><br>Section 9.6.2 and 9.6.3:<br>- Updated visit schedule to include nasal biopsy visits<br><br>Section 9.6.4:<br>-Updated blood collection chart to include coagulation<br><br>Section 9.7.2:<br>- Inclusion of nasal biopsies for participants who volunteer for this additional sampling.<br><br>Section 9.8.3<br>-Inclusion of spatial transcriptomics in laboratory processes<br><br>Changes to the PIS for Group A and Group B:<br>-Updated with the nasal biopsy procedure.<br><br>Changes to Reimbursement Information Sheet:<br>Updated with the reimbursement for nasal biopsy visit.<br><br>Additional documents:<br>A new document was also created to provide participants information regarding the nasal biopsy procedure:<br>-Nasal biopsy PIS and ICF<br><br>Typographical corrections
3	V4.0	Katrina Pollock, Daniela Ferreira, Oisin Hennigan, Britta Urban, Hannah Baughan, Thejaswini Madupuri, Carla Solorzano-Gonzalez	Change age range of older participants from 60-80 to 55-80 throughout<br><br>Section 7 - Figure 2<br>-Updated Group B study visit to remove PBMCs at D3 visit<br><br>9.6.4 Blood Volumes - Table 4<br>- Corrected table to match section 7 – Figure 2<br>- update to max total in Group B table<br><br>Additional documents<br>ECLIPSE Informed Consent Quiz Answer sheet<br>- Question 2: changed to include influenza virus as an answer<br>ECLIPSE GP Withdrawal Letter<br>- Added optional text to include information about withdrawal of Group B participants<br>ECLIPSE Group A Visit Appointment Card<br>- Changed Vaccination/LAIV timeline to match protocol<br>ECLIPSE Group B Visit Appointment Card<br>Changed wording of baseline visit to match protocol<br>ECLIPSE GP screening letter

			- Change age range of older participants from 60-80 to 55-80 throughout<br>ECLIPSE Group A PIS<br>- Change age range of older participants from 60-80 to 55-80 throughout<br>ECLIPSE Group B PIS<br>- Change age range of older participants from 60-80 to 55-80 throughout<br>ECLIPSE Online Screening Questionnaire<br>- Change age range of older participants from 60-80 to 55-80 throughout<br>ECLIPSE Recruitment Material<br>- Change age range of older participants from 60-80 to 55-80 throughout<br>ECLIPSE Recruitment Posters<br>- Change age range of older participants from 60-80 to 55-80 throughout<br>ECLIPSE Be Part of Research Volunteer Service Template email<br>- Change age range of older participants from 60-80 to 55-80 throughout<br>- Fluenz nasal spray suspension Influenza vaccine (live, nasal) – SmPC – 2025<br>- ECLIPSE Nasal Biopsy PIS and ICF
NSA01	V4.1	Thejaswini Madupuri	Updates included in Non-substantial Amendment 01:<br><br>Section 3: The revised planned recruitment period will be from 01 Sep 2024 to 31 March 2026 to allow for recruitment of the new age range 55 – 80.
NSA02	V4.2	Vivian Yim, Thejaswini Madupuri	Updates included in non-substantial amendment 02:<br><br>Section 8.3 is updated to clarify the exclusion criteria to ensure that allergy to penicillin, amoxicillin and/or gentamicin is only relevant to Group A.
