# Supplementary material for: Establishing a human co-infection challenge study of Streptococcus pneumoniae (Spn) and live attenuated influenza vaccine (LAIV) in older healthy adults: Exploring Co-infection with Live Attenuated Influenza Vaccine and PneumococcuS in healthy oldEr adults (ECLIPSE), a UK-based single-centre, single-blind randomised controlled trial
Source: BMJ Open. 2026 Oct 12;16(10):e118632. doi: 10.1136/bmjopen-2026-118632 (PMC13662028; doi:10.1136/bmjopen-2026-118632)
Supplement: Supplementary file 6 [file bmjopen-16-10-s008.pdf]

Oxford Vaccine Group
University of Oxford
Centre for Clinical Vaccinology and Tropical Medicine,
Churchill Hospital, Headington, Oxford OX3 7LE
Telephone: 01865 611400 info@ovg.ox.ac.uk www.ovg.ox.ac.uk

Participant Identification Number: ______________________________

ECLIPSE

Exploring **C**o-infection with **L**ive Attenuated **I**nfluenza Virus and **P**neumococcu**S** in healthy old**E**r adults

Informed Consent Form – Group B

Participant's Name: ______________________________________ Initials: ___ ___ ___

*Please **initial** if you agree with the statement*

**Section 1: Study Procedures**	
1. I confirm that I have read the ECLIPSE Participant Information Sheet Group B, version___.___ dated _ _ - _ _ _ - _ _ _ _. I confirm that the study procedures and information have been explained to me. I have had the opportunity to consider the information, ask questions and have had these answered satisfactorily.	
2. I have spoken with Dr/Nurse ____________________	
3. I understand that my participation is voluntary and that I am free to withdraw at any time without giving any reason, without my medical care or legal rights being affected.	
4. I have received detailed information about the treatment schedule and potential side effects.	
5. I agree to receive intranasal Live Attenuated Influenza Vaccine	
6. I agree that the research team may share, use and store my personal information as described in the participant information sheet.	
7. I understand that I must notify study team if I am taking a vaccine, or any new medications as this may mean I am excluded from the study.	
**Section 2: Personal Information**	
8. I understand that relevant sections of my medical notes and data collected during the study including identifiable information may be looked at by individuals from Oxford Vaccine Group, from regulatory authorities, from the NHS Trust, and authorised representatives appointed by the Sponsor (University of Oxford), where it is relevant to my taking part in this research. I give permission for these individuals to have access to my records.	
9. I agree to my General Practitioner and/or consultant (if appropriate) being informed of my participation in the study.	
10. I agree to my General Practitioner and/or other treating doctors and/or any other healthcare provider service being approached for additional information regarding medical and vaccination history and study staff to access my NHS medical records either via my GP or electronic patient records system or equivalent NHS database.	
11. I understand TOPS is the Health Research Authority database that aims to prevent healthy volunteers from taking part in too many studies. I understand that only staff	

Participant Identification Number: ___________________________

at Oxford Vaccine Group and other research units can use the database, and the study team will check volunteer details. I agree to my National Insurance (if UK citizen), or Passport number being used to register me on TOPS. I understand that it will be stored electronically for the duration of the study.	
12. I agree to provide my bank account details including my account name, sort code and account number for reimbursement purposes. I understand that my bank details will be stored electronically as described in the information booklet. I understand that my personal information will be shared to the extent required to process or verify eligibility of payments as described in the information booklet.	
13. I understand and agree that the Sponsor Data Management and IT Team will be able to view my email address. I understand it will be held securely on a server at the University of Oxford.	
**Section 3: Research Samples and Data**	
14. I agree to donate blood, saliva, throat swabs and nasal samples. I consider these samples a gift to the University of Oxford, and I understand that I will not gain any direct personal or financial benefit from them.	
15. I agree to have blood tests as part of this trial, including testing for HIV, Hepatitis B and Hepatitis C. I agree to the results of these tests being reported to UKHSA as outlined in the participant information sheet if required.	
16. I understand and agree that my anonymised data and samples will be stored and used as outlined in the participant information sheet and this consent form and can be transferred outside of the UK (including outside the EU) for the purposes of this study to other collaborators.	
17. I understand that some of my samples will be used to investigate genetic factors affecting infection and immunity and I understand that the results of these investigations will not have any implications for me personally.	
**Section 4: <u>Optional</u>** (Write 'N/A' and initial outside the box if not applicable.)	
18. **Females of childbearing potential ONLY:** I confirm that I am not planning to conceive, and I will use effective contraception during the study.	
**If all sentences above are initialled, meaning "yes", then please continue:**	
19. I voluntarily agree to take part in this study	

**Section 5: <u>Optional:</u>**		
Please initial in the relevant column (Yes or No) as appropriate	YES	NO
1. I agree to be contacted about ethically approved research studies for which I may be suitable. I understand that agreeing to be contacted does not oblige me to participate in any further studies.		
2. I agree to have a fine needle aspirate (FNA) sample taken from both armpits, and I am aware of the associated risks. I understand that there would be a		

Participant Identification Number: ___________________________

separate discussion and consent at a later stage, and understand I am not guaranteed to be offered this.		

_____________________________	________________________	_ _ / _ _ _ / _ _ _ _
Name of participant (print)	Signature	Date *(DD/MON/YYYY)*
_____________________________	________________________	_ _ / _ _ _ / _ _ _ _
Name of doctor/nurse taking consent	Signature	Date *(DD/MON/YYYY)*

Original to be retained at site in the case report form (CRF); second copy to be given to the participant.
