# Supplementary material for: Establishing a human co-infection challenge study of Streptococcus pneumoniae (Spn) and live attenuated influenza vaccine (LAIV) in older healthy adults: Exploring Co-infection with Live Attenuated Influenza Vaccine and PneumococcuS in healthy oldEr adults (ECLIPSE), a UK-based single-centre, single-blind randomised controlled trial
Source: BMJ Open. 2026 Oct 12;16(10):e118632. doi: 10.1136/bmjopen-2026-118632 (PMC13662028; doi:10.1136/bmjopen-2026-118632)
Supplement: Supplementary file 7 [file bmjopen-2026-118632.reviewer_comments.pdf]

PEER REVIEW HISTORY

BMJ Open publishes all reviews undertaken for accepted manuscripts. Reviewers are asked to complete a checklist review form and are provided with free text boxes to elaborate on their assessment. These free text comments are reproduced below.

ARTICLE DETAILS

Title (Provisional)

Establishing a human co-infection challenge study of S. pneumoniae (Spn) and Live Attenuated Influenza Vaccine (LAIV) in older healthy adults: Exploring Co-infection with Live Attenuated Influenza Vaccine and PneumococcuS in healthy oldEr adults (ECLIPSE), a UK based single centre, single blind randomised controlled trial

Authors

Jackson, Rebecca; Hennigan, Oisin; Kelleher, Karen; Solórzano, Carla; Mitsi, Elena; Morey, Ella; Madupuri, Thejaswini; Singh, Nisha; Baughan, Hannah; Patel, Bhumika; Verleur, Nile; Codreanu, Tatiana; Elterish, Filora; Comma, Ebrima; Mujadidi, Yama F; Plested, Emma; Robinson, Hannah; Brito-Mutunayagam, Sanjita; Liu , Xinxue; Didierlaurent, Arnaud M.; Urban, Britta C.; Pollock, Katrina; Ferreira, Daniela M.

VERSION 1 - REVIEW

**Reviewer**	**1**
**Name**	**Xochitl, Trujillo**
**Affiliation**	**Universidad de Colima, Centro Universitario de Investigaciones Biomedicas**
**Date**	**24-May-2026**
**COI**	**None**

This research protocol is clearly defined and join the needs according to CONSORT for the Results presentation. Being a protocol it have not Results, Discussion and Conclusions. The design is according and I have not additional comments for authors.

**Reviewer**	**2**
**Name**	**Dopelt, Keren**
**Affiliation**	**Ben-Gurion University of the Negev, Health Policy and Management**
**Date**	**29-May-2026**
**COI**	**None**

The protocol is well structured, with a detailed description of the study design, interventions, follow-up schedule, sampling procedures, safety monitoring, and planned analyses. I have only a few minor comments:

The limitations section could be expanded. In particular, add a discussing regarding the non-randomized nature and small sample size of the LAIV-only control cohort, especially in relation to age-stratified comparisons.

One sentence in the abstract could be clarified: "Throughout the study, we will take samples from the nose, throat, saliva, and blood to look at how the immune system responds and keep a diary to follow immune cells and markers." As currently written, this could be read as though the diary is used to follow immune cells and markers. Please revise this sentence to separate biological sample collection from symptom diary completion.

Please clarify the terminology around the control cohort. Group B is described as a control group receiving LAIV alone and is not randomized or blinded. Please state more clearly that comparisons involving this cohort are exploratory and may be limited by the allocation procedure and small sample size.

The sample size justification is acceptable for an exploratory protocol. However, it would be useful to provide a little more detail on how the proposed numbers are expected to support the secondary immunological and virological analyses, particularly for Group B and subgroup comparisons.

Reviewer 3

Name Cardoso, Kimberly

Affiliation Instituto René Rachou/Fiocruz Minas, Belo Horizonte, MG, BrasiLaboratório de Imunologia de Doenças Virais, Instituto René Rachou-Fundação Oswaldo Cruz, Belo Horizonte, Minas Gerais, Brasil

Date 27-Jun-2026

COI None

This protocol describes a novel and clinically relevant controlled human co-infection challenge study addressing an important knowledge gap regarding pneumococcal–influenza interactions in older adults. Overall, the study is well conceived, addresses an important research question, and the protocol is generally well designed.

I believe the manuscript would benefit from several revisions to improve the transparency and reproducibility of the study protocol. My main comments relate to the justification of the proposed sample size, the statistical analysis plan for longitudinal data, the management of concomitant respiratory infections during follow-up, and the presentation of the predefined stopping rules. I also provide several minor suggestions that may improve the clarity and interpretation of the manuscript.

For ease of review, my detailed comments are provided in the attached document.

Human co-infection challenge study of S. pneumoniae (Spn) and Live Attenuated Influenza Vaccine (LAIV) in older healthy adults: Exploring Co-infection with Live Attenuated Influenza Vaccine and Pneumococcus in healthy older adults (ECLIPSE): study protocol

This protocol describes a novel and potentially important controlled human co-infection challenge study addressing a significant knowledge gap regarding pneumococcal–influenza interactions in older adults. Overall, the study is well conceived, addresses an important clinical question, and appears to have been carefully designed with appropriate ethical oversight. Nevertheless, several methodological aspects would benefit from further clarification before publication, particularly regarding the management of concomitant respiratory infections, the statistical analysis of longitudinal data, the justification of the proposed sample size, and the presentation of the study stopping rules. Addressing these points would improve the transparency, reproducibility, and interpretability of the protocol, ultimately strengthening the value of the future study findings.

Major concerns

1. Management of concomitant respiratory infections during follow-up

Pages 13 and 14 – Study Procedures (lines 55 – 60 on page 13 and lines 3 – 8 on page 14).

The protocol indicates that respiratory pathogens will be monitored through viral PCR and microbiological analyses, and asymptomatic respiratory infections are listed among the secondary outcomes. However, the manuscript does not describe how participants who acquire respiratory infections during follow-up will be handled in the statistical analyses. This issue is particularly important because the primary objective of the study is to evaluate the effects of the order of exposure to LAIV and *Streptococcus pneumoniae* on pneumococcal carriage, viral load, respiratory symptoms and mucosal immune responses. Intercurrent respiratory infections (e.g., rhinovirus, RSV, seasonal coronaviruses or naturally acquired influenza) may independently influence these same outcomes by altering local inflammation, cytokine production, immune cell

recruitment, pneumococcal colonization dynamics and viral replication. Consequently, these infections may act as important confounding factors, making it difficult to distinguish changes attributable to the experimental intervention from those resulting from naturally acquired respiratory pathogens.

Furthermore, given that the study plans to include approximately 20 participants per intervention arm, even a small number of participants developing intercurrent respiratory infections could substantially increase data variability, thereby reducing the ability to detect true differences between study groups, or reduce the effective sample size if such participants require exclusion from specific analyses. Clarifying the planned analytical approach is therefore particularly important to ensure that these events do not compromise the interpretation and robustness of the study findings.

Although surveillance for respiratory pathogens is described, the manuscript does not specify:

- whether participants with concomitant respiratory infections will remain in the primary analyses;
- whether these participants will be excluded from specific analyses;
- whether sensitivity analyses will be performed;
- whether respiratory infections will be incorporated as covariates or adjustment variables in the statistical models.

Providing this information would improve the transparency and interpretability of the study findings and strengthen confidence that observed differences in immune responses and pathogen dynamics are attributable to the experimental intervention rather than to unrelated respiratory infections acquired during follow-up.

Comment to the authors: Please clarify how concomitant respiratory infections detected during follow-up will be handled in the statistical analyses. Specifically, please indicate whether these infections will be considered potential confounding factors, whether adjustment or sensitivity analyses are planned, and how their potential impact on immunological and microbiological outcomes will be addressed.

2. Stopping rules are only referenced but not described

Page 15 – Auditing (lines 46 – 50)

The manuscript states that predefined stopping rules are available in Appendix 5. However, no summary of these rules is presented within the main manuscript. Considering that this is a controlled human challenge study involving adults aged 55 – 80 years, readers would benefit from understanding the principal safety criteria that could lead to study interruption.

Comment to the authors: Although the stopping rules are referenced in Appendix 5, I recommend including a summary of the main stopping criteria in the manuscript to improve transparency regarding participant safety.

3. Statistical analysis plan for longitudinal data

Pages 15 and 16 – Statistical methods (lines 58 – 59 on page 15 and lines 3 – 18 on page 16)

The statistical analysis section states that immune response data will be log-transformed, summarized as geometric mean concentrations (GMCs) with 95% confidence intervals, and analyzed using the per-protocol population. However, the analytical strategy remains insufficiently detailed considering the complexity of the study design.

The protocol includes repeated assessments from the same participants at multiple time points (e.g., pneumococcal carriage, viral load, mucosal immune responses, blood biomarkers, transcriptomic analyses and clinical symptoms). Because these measurements are obtained repeatedly from the same individual, they are not statistically independent and generally require analytical approaches that account for the correlation between repeated observations. Although descriptive methods are presented, the manuscript does not specify:

- which statistical models will be used to analyse the primary and secondary outcomes;
- whether longitudinal or repeated-measures approaches (e.g., mixed-effects models or generalized estimating equations) will be used to account for within-subject correlations over time;
- how baseline differences or potential confounding variables will be handled;
- whether adjustments for multiple comparisons will be applied, considering the large number of immunological and molecular endpoints evaluated.

Providing these methodological details is important to improve the transparency, reproducibility and interpretability of the planned analyses, particularly given the longitudinal nature of the study and the broad range of exploratory immunological outcomes.

Comment to the authors: Please provide additional details regarding the planned statistical analysis of the longitudinal data, including the models that will be used to account for repeated measurements within participants. In addition, please clarify how multiple immunological endpoints and potential confounding factors will be addressed during statistical analyses.

4. Sample size justification

Page 16 – Sample Size Determination (lines 30 – 46)

The manuscript states that up to 60 participants will be randomized, with approximately 40 participants completing the study (20 per intervention arm). The proposed sample size is described as based on feasibility and considered sufficient to identify gene signatures. However, the justification for the planned sample size remains limited, particularly considering the broad range of secondary immunological, microbiological and clinical outcomes included in the protocol.

Specifically, the manuscript does not describe:

- the expected effect size;
- the statistical power (if applicable);
- the variability estimates considered;
- the primary parameter used to support the proposed sample size.

Although a formal sample size calculation may not be required for a feasibility study, additional justification would strengthen the rationale for the proposed sample size, particularly given the longitudinal study design and the extensive number of planned exploratory analyses, including pneumococcal carriage, viral load, cytokine profiling, transcriptomics, PBMC analyses and single-cell sequencing.

Furthermore, because only approximately 20 participants are expected per

intervention arm, the precision of these exploratory analyses may be particularly sensitive to increased biological variability or to the loss of evaluable participants during follow-up. Providing additional justification for the chosen sample size would therefore improve confidence that the planned study will adequately address its secondary objectives.

Comment to the authors: Although this is primarily a feasibility study, please provide additional justification for how the proposed sample size was considered sufficient to support the planned secondary immunological, microbiological and clinical analyses.

Minor concerns

1. **Potential selection bias in Group B**

Page 12 – Allocation (lines 38 – 40)

The protocol states that older adults are initially considered for inclusion in Group A and that participants who decline pneumococcal inoculation may subsequently be recruited into Group B. Consequently, allocation to Group B depends, at least in part, on participant preference rather than random allocation. Participants who choose not to undergo pneumococcal challenge may differ systematically from those who agree to participate in the challenge with respect to characteristics that are not captured by the eligibility criteria, such as risk perception, willingness to undergo an invasive intervention, or other unmeasured participant characteristics. These differences could potentially influence study outcomes independently of the intervention itself, particularly when comparisons are made between Groups A and B.

Although Group B appears to function primarily as an exploratory control cohort rather than a randomized comparator, acknowledging this potential source of selection bias would help readers interpret any between-group comparisons more appropriately.

Comment to the authors: Please consider acknowledging this potential source of selection bias in the limitations section and briefly discuss how it may influence the interpretation of comparisons involving Group B.

2. **Additional information regarding off-label use of LAIV**

Page 14 – Vaccination with LAIV/saline administration (lines 40 – 43)

The manuscript appropriately acknowledges that LAIV will be administered outside its licensed indication, as it is approved for use in children but will be administered to adults aged 55–80 years in this study. The authors state that previous studies support the safety of LAIV in this population; however, this statement is not accompanied by a brief summary of the available evidence or by explicit references to the key supporting studies.

Given that the off-label administration of a live attenuated vaccine in older adults represents an important aspect of the study design, providing a concise overview of the available safety data would strengthen the scientific rationale and allow readers to better appreciate the evidence supporting this approach.

Comment to the authors: Please consider expanding the rationale for the off-label use of LAIV in adults aged 55–80 years by briefly summarizing the available safety evidence or by explicitly citing the key studies supporting this decision.

VERSION 1 - AUTHOR RESPONSE

Reviewer: 1

Dr. Trujillo Xochitl, Universidad de Colima

Comments to the Author:

This research protocol is clearly defined and join the needs according to CONSORT for the Results presentation. Being a protocol it have not Results, Discussion and Conclusions. The design is according and I have not additional comments for authors.
Thank you for taking the time to review the manuscript.

Reviewer: 2

Dr. Keren Dopelt, Ben-Gurion University of the Negev

Comments to the Author:

The protocol is well structured, with a detailed description of the study design, interventions, follow-up schedule, sampling procedures, safety monitoring, and planned analyses. I have only a few minor comments:

The limitations section could be expanded. In particular, add a discussing regarding the non-randomized nature and small sample size of the LAIV-only control cohort, especially in relation to age-stratified comparisons.

We have added a statement to the limitations section (page 2), and a statement in the study design section (page 6). .

One sentence in the abstract could be clarified: “Throughout the study, we will take samples from the nose, throat, saliva, and blood to look at how the immune system responds and keep a diary to follow immune cells and markers.” As currently written, this could be read as though the diary is used to follow immune cells and markers. Please revise this sentence to separate biological sample collection from symptom diary completion.

Thank you, this is indeed awkward. We have corrected the sentence in the abstract.

Please clarify the terminology around the control cohort. Group B is described as a control group receiving LAIV alone and is not randomized or blinded. Please state more clearly that comparisons involving this cohort are exploratory and may be limited by the allocation procedure and small sample size.

Thank you, we have added a sentence to the Study Rationale section to specifiy that this group allows us to distinguish the impact of LAIV vaccination alone from co-infection with Spn6B on mucosal and systemic immune responses in an exploratory analysis (page 3-4).

The sample size justification is acceptable for an exploratory protocol. However, it would be useful to provide a little more detail on how the proposed numbers are expected to support the secondary immunological and virological analyses, particularly for Group B and subgroup comparisons.

Thank you for this helpful comment. We have expanded the *Sample Size Determination* and *Statistical Methods* sections to better justify the proposed sample size. We now clarify that ECLIPSE is designed as a mechanistic experimental medicine study, with the primary objective of assessing the feasibility and safety of the co-infection challenge model. The proposed sample size is based on feasibility, previous experience with the EHPC model, and the goal to generate rich longitudinal mucosal and systemic immunological datasets. We also explain how the repeated longitudinal sampling design enables mechanistic and hypothesis-generating analyses despite the relatively small number of participants. Finally, we clarify that analyses involving Group B and age-stratified subgroup comparisons are exploratory and are intended to characterise immune responses to LAIV alone and generate estimates for future confirmatory studies.

Reviewer: 3

Dr. Kimberly Cardoso, Instituto René Rachou/Fiocruz Minas, Belo Horizonte, MG, BrasiLaboratório de Imunologia de Doenças Virais, Instituto René Rachou-Fundação Oswaldo Cruz, Belo Horizonte, Minas Gerais, Brasil

Comments to the Author:

This protocol describes a novel and clinically relevant controlled human co-infection challenge study addressing an important knowledge gap regarding pneumococcal–influenza interactions in older adults. Overall, the study is well conceived, addresses an important research question, and the protocol is generally well designed.

I believe the manuscript would benefit from several revisions to improve the transparency and reproducibility of the study protocol. My main comments relate to the justification of the proposed sample size, the statistical analysis plan for longitudinal data, the management of concomitant respiratory infections during follow-up, and the presentation of the predefined

stopping rules. I also provide several minor suggestions that may improve the clarity and interpretation of the manuscript.

We thank the reviewer for their careful evaluation of our manuscript and believe they will make the manuscript more transparent and improve clarity.

Human co-infection challenge study of S. pneumoniae (Spn) and Live Attenuated Influenza Vaccine (LAIV) in older healthy adults: Exploring Co-infection with Live Attenuated Influenza Vaccine and Pneumococcus in healthy older adults (ECLIPSE): study protocol

This protocol describes a novel and potentially important controlled human co-infection challenge study addressing a significant knowledge gap regarding pneumococcal–influenza interactions in older adults. Overall, the study is well conceived, addresses an important clinical question, and appears to have been carefully designed with appropriate ethical oversight. Nevertheless, several methodological aspects would benefit from further clarification before publication, particularly regarding the management of concomitant respiratory infections, the statistical analysis of longitudinal data, the justification of the proposed sample size, and the presentation of the study stopping rules. Addressing these points would improve the transparency, reproducibility, and interpretability of the protocol, ultimately strengthening the value of the future study findings.

Major concerns
1. Management of concomitant respiratory infections during follow-up
Pages 13 and 14 – Study Procedures (lines 55 – 60 on page 13 and lines 3 – 8 on page 14).
The protocol indicates that respiratory pathogens will be monitored through viral PCR and microbiological analyses, and asymptomatic respiratory infections are listed among the secondary outcomes. However, the manuscript does not describe how participants who acquire respiratory infections during follow-up will be handled in the statistical analyses. This issue is particularly important because the primary objective of the study is to evaluate the effects of the order of exposure to LAIV and *Streptococcus pneumoniae* on pneumococcal carriage, viral load, respiratory symptoms and mucosal immune responses. Intercurrent respiratory infections (e.g., rhinovirus, RSV, seasonal coronaviruses or naturally acquired influenza) may independently influence these same outcomes by altering local inflammation, cytokine production, immune cell recruitment, pneumococcal colonization dynamics and viral replication. Consequently, these infections may act as important confounding factors, making it difficult to distinguish changes attributable to the experimental intervention from those resulting from naturally acquired respiratory pathogens.
Furthermore, given that the study plans to include approximately 20 participants per intervention arm, even a small number of participants developing intercurrent respiratory infections could substantially increase data variability, thereby reducing the ability to detect true differences between study groups, or reduce the effective sample size if such participants require exclusion from specific analyses. Clarifying the planned analytical approach is therefore particularly important to ensure that these events do not compromise the interpretation and robustness of the study findings.
Although surveillance for respiratory pathogens is described, the manuscript does not specify:

- **whether participants with concomitant respiratory infections will remain in the primary analyses;**
- **whether these participants will be excluded from specific analyses;**

• whether sensitivity analyses will be performed;
• whether respiratory infections will be incorporated as covariates or adjustment variables in the statistical models.
Providing this information would improve the transparency and interpretability of the study findings and strengthen confidence that observed differences in immune responses and pathogen dynamics are attributable to the experimental intervention rather than to unrelated respiratory infections acquired during follow-up.

Comment to the authors: Please clarify how concomitant respiratory infections detected during follow-up will be handled in the statistical analyses. Specifically, please indicate whether these infections will be considered potential confounding factors, whether adjustment or sensitivity analyses are planned, and how their potential impact on immunological and microbiological outcomes will be addressed.

We agree that naturally acquired respiratory infections may influence mucosal immune responses. Participants with respiratory infections will remain in the primary analyses, with respiratory pathogens considered as exploratory covariates or sensitivity analyses where appropriate. Previous work from our group has demonstrated that asymptomatic viral infection can influence pneumococcal carriage dynamics, supporting this approach. We have clarified this in the statistical analysis section (page 13). Additionally, we have added the list of temporary exclusions for inoculation (table 1) to highlight that we will attempt to minimise respiratory infections prior to inoculation.

2. Stopping rules are only referenced but not described *Page 15 – Auditing (lines 46 – 50)*

The manuscript states that predefined stopping rules are available in Appendix 5. However, no summary of these rules is presented within the main manuscript. Considering that this is a controlled human challenge study involving adults aged 55 – 80 years, readers would benefit from understanding the principal safety criteria that could lead to study interruption.

Comment to the authors: Although the stopping rules are referenced in Appendix 5, I recommend including a summary of the main stopping criteria in the manuscript to improve transparency regarding participant safety.

We thank the reviewer for this comment which will improve clarity of the manuscript. We have now stated the criteria for stopping rules in the Auditing section of the manuscript (page 15).

3. Statistical analysis plan for longitudinal data

Pages 15 and 16 – Statistical methods (lines 58 – 59 on page 15 and lines 3 – 18 on page 16)

The statistical analysis section states that immune response data will be log-transformed, summarized as geometric mean concentrations (GMCs) with 95% confidence intervals, and analyzed using the per-protocol population. However, the analytical strategy remains insufficiently detailed considering the complexity of the study design.
The protocol includes repeated assessments from the same participants at multiple time points (e.g., pneumococcal carriage, viral load, mucosal immune responses, blood biomarkers, transcriptomic analyses and clinical symptoms). Because these measurements are obtained repeatedly from the same individual, they are not statistically independent and generally require analytical approaches that account for the correlation between repeated observations. Although

descriptive methods are presented, the manuscript does not specify:
• which statistical models will be used to analyse the primary and secondary outcomes;
• whether longitudinal or repeated-measures approaches (e.g., mixed-effects models or generalized estimating equations) will be used to account for within-subject correlations over time;
• how baseline differences or potential confounding variables will be handled;
• whether adjustments for multiple comparisons will be applied, considering the large number of immunological and molecular endpoints evaluated.

Providing these methodological details is important to improve the transparency, reproducibility and interpretability of the planned analyses, particularly given the longitudinal nature of the study and the broad range of exploratory immunological outcomes.

Comment to the authors: Please provide additional details regarding the planned statistical analysis of the longitudinal data, including the models that will be used to account for repeated measurements within participants. In addition, please clarify how multiple immunological endpoints and potential confounding factors will be addressed during statistical analyses.

We thank the reviewer for this helpful suggestion. We have substantially expanded the Statistical Methods section to clarify the planned analytical approach. We now specify that longitudinal outcomes will be analysed using repeated-measures regression models that account for within-participant correlation over time. We also clarify that the principal biological focus of the study is the longitudinal analysis of immune responses within the nasal mucosa, with peripheral blood analysed as a complementary systemic comparator. Baseline measurements and clinically relevant covariates will be incorporated where appropriate. Furthermore, we now state that high-dimensional datasets, including single-cell RNA sequencing, are exploratory and hypothesis-generating, with false discovery rate adjustment applied where appropriate for high-dimensional analyses.

4. Sample size justification

Page 16 – Sample Size Determination (lines 30 – 46)
The manuscript states that up to 60 participants will be randomized, with approximately 40 participants completing the study (20 per intervention arm). The proposed sample size is described as based on feasibility and considered sufficient to identify gene signatures. However, the justification for the planned sample size remains limited, particularly considering the broad range of secondary immunological, microbiological and clinical outcomes included in the protocol.

Specifically, the manuscript does not describe:
• the expected effect size;
• the statistical power (if applicable);
• the variability estimates considered;
• the primary parameter used to support the proposed sample size.

Although a formal sample size calculation may not be required for a feasibility study, additional justification would strengthen the rationale for the proposed sample size, particularly given the longitudinal study design and the extensive number of planned exploratory analyses, including pneumococcal carriage, viral load, cytokine profiling, transcriptomics, PBMC analyses and single-cell sequencing.

Furthermore, because only approximately 20 participants are expected per intervention arm, the precision of these exploratory analyses may be particularly sensitive to increased biological variability or to the loss of evaluable participants during follow-up. Providing additional justification for the chosen sample size would therefore improve confidence that the planned study will adequately address its secondary objectives.

Comment to the authors: Although this is primarily a feasibility study, please provide additional justification for how the proposed sample size was considered sufficient to support the planned secondary immunological, microbiological and clinical analyses.

We thank the reviewer for this helpful comment. We agree that the rationale for the sample size required further clarification. As this is primarily a mechanistic experimental medicine study, the sample size was not based on formal hypothesis testing for the secondary immunological and virological endpoints. Instead, it was determined by feasibility and safety considerations, previous experience with the EHPC model, and earlier human LAIV-Spn co-infection studies that successfully characterised pneumococcal carriage dynamics, viral load and mucosal immune responses with a similar number of study participants. The principal analyses focus on repeated sampling of the nasal mucosa, including single-cell transcriptomic analyses, which are intended to generate mechanistic hypotheses and estimates of biological variability to inform the design of future confirmatory studies. We have revised the Sample Size Determination section accordingly.

Minor concerns
1. Potential selection bias in Group B
Page 12 – Allocation (lines 38 – 40)
The protocol states that older adults are initially considered for inclusion in Group A and that participants who decline pneumococcal inoculation may subsequently be recruited into Group B. Consequently, allocation to Group B depends, at least in part, on participant preference rather than random allocation. Participants who choose not to undergo pneumococcal challenge may differ systematically from those who agree to participate in the challenge with respect to characteristics that are not captured by the eligibility criteria, such as risk perception, willingness to undergo an invasive intervention, or other unmeasured participant characteristics. These differences could potentially influence study outcomes independently of the intervention itself, particularly when comparisons are made between Groups A and B. Although Group B appears to function primarily as an exploratory control cohort rather than a randomized comparator, acknowledging this potential source of selection bias would help readers interpret any between-group comparisons more appropriately.

Comment to the authors: Please consider acknowledging this potential source of selection bias in the limitations section and briefly discuss how it may influence the interpretation of comparisons involving Group B.

We thank the reviewer for this comment and have addressed this under the strength and limitations section and stated in the sample size section that comparisons involving group B are exploratory.

2. Additional information regarding off-label use of LAIV
Page 14 – Vaccination with LAIV/saline administration (lines 40 – 43)
The manuscript appropriately acknowledges that LAIV will be administered outside its licensed indication, as it is approved for use in children but will be administered to adults aged 55–80 years in this study. The authors state that previous studies support the safety of LAIV in this population; however, this

statement is not accompanied by a brief summary of the available evidence or by explicit references to the key supporting studies.
Given that the off-label administration of a live attenuated vaccine in older adults represents an important aspect of the study design, providing a concise overview of the available safety data would strengthen the scientific rationale and allow readers to better appreciate the evidence supporting this approach.

Comment to the authors: Please consider expanding the rationale for the off-label use of LAIV in adults aged 55–80 years by briefly summarizing the available safety evidence or by explicitly citing the key studies supporting this decision.

Thank you, we now explicitly cite and summarise two large randomised clinical trials addressing the safety of LAIV in older adults (page 12).

VERSION 2 - REVIEW

**Reviewer**	**3**
**Name**	**Cardoso, Kimberly**
**Affiliation**	**Instituto René Rachou/Fiocruz Minas, Belo Horizonte, MG, BrasiLaboratório de Imunologia de Doenças Virais, Instituto René Rachou-Fundação Oswaldo Cruz, Belo Horizonte, Minas Gerais, Brasil**
**Date**	**02-Sep-2026**
**COI**	

The authors have carefully addressed the comments and concerns raised during the previous round of review. I appreciate the detailed revisions and the additional methodological clarifications provided in the revised manuscript.

The responses adequately address my previous concerns, and the manuscript has been improved substantially as a result of these revisions. I have no further major concerns regarding the manuscript and consider that the authors have satisfactorily addressed the issues raised in the previous review.

VERSION 2 - AUTHOR RESPONSE

Reviewer: 3
Dr. Kimberly Cardoso, Instituto René Rachou/Fiocruz Minas, Belo Horizonte, MG, BrasiLaboratório de Imunologia de Doenças Virais, Instituto René Rachou-Fundação Oswaldo Cruz, Belo Horizonte, Minas Gerais, Brasil

Comments to the Author:
The authors have carefully addressed the comments and concerns raised during the previous round of review. I appreciate the detailed revisions and the additional methodological clarifications provided in the revised manuscript.

The responses adequately address my previous concerns, and the manuscript has been improved substantially as a result of these revisions. I have no further major concerns regarding the manuscript and consider that the authors have satisfactorily addressed the issues raised in the previous review.

- Thank you for taking the time to review the manuscript and for your helpful comments
