# Supplementary material for: Establishing a human co-infection challenge study of Streptococcus pneumoniae (Spn) and live attenuated influenza vaccine (LAIV) in older healthy adults: Exploring Co-infection with Live Attenuated Influenza Vaccine and PneumococcuS in healthy oldEr adults (ECLIPSE), a UK-based single-centre, single-blind randomised controlled trial
Source: BMJ Open. 2026 Oct 12;16(10):e118632. doi: 10.1136/bmjopen-2026-118632 (PMC13662028; doi:10.1136/bmjopen-2026-118632)
Supplement: Supplementary file 8 [file bmjopen-16-10-s001.docx]

**Supplementary Table 1: Schedule of Events – Group A**

| **Study Visit** | **Screening** | **Day** | **Day** |  |  | **Day** |  | **Day** | **Day** |  |  |  | **Day** | **Day** |  | **Day** | **Nasal** |
| --- | --- | --- | --- | --- | --- | --- | --- | --- | --- | --- | --- | --- | --- | --- | --- | --- | --- |
| **Day post Spn inoculation** |  | **-7** | **-3** | **-2** | **-1** | **0** | **1** | **2** | **3** | **4** | **5** | **6** | **7** | **9** | **14** | **28** | **Biopsy** |
| **Visit window (days post Spn inoculation)** | **-90 to -5** | **-9 to -4** |  |  |  |  |  |  |  |  |  |  | **7 to 9** | **8 to 10** |  | **27 to 30** | **14 to 60** |
| Consent (Written) | X |  |  |  |  |  |  |  |  |  |  |  |  |  |  |  | (X)^&^ |
| Consent (Verbal) | X | X | X |  |  | X |  | X | X |  |  |  | X | X |  | X | X |
| Study information | X |  |  |  |  |  |  |  |  |  |  |  |  |  |  |  |  |
| Study screening (inc. medical history) | X |  |  |  |  |  |  |  |  |  |  |  |  |  |  |  |  |
| Clinical Exam | X | X |  |  |  |  |  |  |  |  |  |  |  |  |  |  |  |
| Vital Signs | X | X | X |  |  | X |  | X | X |  |  |  | X | X |  | X | X^%^ |
| Screen for AEs |  |  | X |  |  | X |  | X | X |  |  |  | X | X |  | X | X |
| Concomitant medications | X | X | X |  |  | X |  | X | X |  |  |  | X | X |  | X | X |
| Screening bloods* | X |  |  |  |  |  |  |  |  |  |  |  |  |  |  |  |  |
| ECG | X |  |  |  |  |  |  |  |  |  |  |  |  |  |  |  |  |
| Randomisation |  |  | X |  |  |  |  |  |  |  |  |  |  |  |  |  |  |
| Spn inoculation |  |  |  |  |  | X |  |  |  |  |  |  |  |  |  |  |  |
| LAIV/saline^1^ or saline/LAIV^2^ |  |  | X^1^ |  |  |  |  |  | X^2^ |  |  |  |  |  |  |  |  |
| Nasal biopsy^**^ |  |  |  |  |  |  |  |  |  |  |  |  |  |  |  |  | X |
| Lymph node fine needle aspiration |  |  |  |  |  |  |  |  | X | | | | | | | |  |
| Nasosorption ((X) home sampling) |  | X |  | (X) | (X) | X | (X) | X | X | (X) | (X) | (X) | X | X | (X) | X |  |
| Nasal cells |  | X |  |  |  |  |  | X |  |  |  |  | X | X |  | X |  |
| Nasal wash |  | X |  |  |  |  |  | X |  |  |  |  | X | X |  | X |  |
| Throat swab |  | X |  |  |  | X |  | X | X |  |  |  | X | X |  | X |  |
| Saliva ((X) home sampling) |  | X |  | (X) | (X) | X | (X) | X | X | (X) | (X) | (X) | X | X | (X) | X |  |
| Research bloods |  | X |  |  |  | X |  |  |  |  |  |  | X |  |  | X |  |
| Safety bloods |  | X ^$^ |  |  |  |  |  |  |  |  |  |  |  |  |  |  |  |

**Supplementary Table 1: Schedule of events – Group A** Please note that Day –30 and Day -7 can occur in the same visit. ***Screening bloods=** FBC, U+E, LFT, HbA1c, RPG, CRP, HIV, Hep B/C, Coagulation (for nasal biopsy cohort only) **^$^**= **Safety bloods** (U+E, LFT, CRP, FBC) are only required during baseline assessment visit if screening bloods were performed >21 days beforehand. **Shaded text =** at any time between these dates depending on clinical staff and participant combined availability. ^&^ = Safety follow-up: Participants after nasal biopsy procedure will be observed as per local NHS hospital guidelines before leaving the facility. A phone safety review will be conducted by the study team at 1 day and 3 weeks after the nasal biopsy. Participants undertaking the optional nasal biopsy will undertake a separate additional written consent for the procedure which will be performed or re-confirmed on the day of the biopsy by the operator. **Optional procedure, up to 5 participants
