# Supplementary material for: Establishing a human co-infection challenge study of Streptococcus pneumoniae (Spn) and live attenuated influenza vaccine (LAIV) in older healthy adults: Exploring Co-infection with Live Attenuated Influenza Vaccine and PneumococcuS in healthy oldEr adults (ECLIPSE), a UK-based single-centre, single-blind randomised controlled trial
Source: BMJ Open. 2026 Oct 12;16(10):e118632. doi: 10.1136/bmjopen-2026-118632 (PMC13662028; doi:10.1136/bmjopen-2026-118632)
Supplement: Supplementary file 9 [file bmjopen-16-10-s002.docx]

**Supplementary Table 2:** **Schedule of Events - Group B**

| **Study Visit** | **Screening/** | **Day** |  |  | **Day** |  |  | **Day** |  |  |  |  |  | **Day** | **Nasal** |
| --- | --- | --- | --- | --- | --- | --- | --- | --- | --- | --- | --- | --- | --- | --- | --- |
| **Day post LAIV** | **Baseline visits** | **0** | **1** | **2** | **3** | **4** | **5** | **6** | **7** | **8** | **9** | **10** | **17** | **31** | **Biopsy** |
| **Visit window** | -90 to –2 ^^^(baseline samples –7 to –2) |  |  |  |  |  |  | 5 to 6 |  |  |  |  |  | 31 to 34 | 14 to 60 |
| Consent (Written) | X |  |  |  |  |  |  |  |  |  |  |  |  |  | (X)^&^ |
| Consent (Verbal) | X | X |  |  | X |  |  | X |  |  |  |  |  | X | X |
| Study information | X |  |  |  |  |  |  |  |  |  |  |  |  |  |  |
| Study screening (inc. medical history) | X |  |  |  |  |  |  |  |  |  |  |  |  |  |  |
| Clinical Exam | X |  |  |  |  |  |  |  |  |  |  |  |  |  |  |
| Vital Signs | X | X |  |  | X |  |  | X |  |  |  |  |  | X | X^%^ |
| Screen for AEs |  | X |  |  | X |  |  | X |  |  |  |  |  | X | X |
| Concomitant medications | X | X |  |  | X |  |  | X |  |  |  |  |  | X | X |
| Pregnancy test | X | X |  |  |  |  |  |  |  |  |  |  |  |  |  |
| Safety bloods^*^ | X |  |  |  |  |  |  |  |  |  |  |  |  |  |  |
| Screening bloods^$^ | X |  |  |  |  |  |  |  |  |  |  |  |  |  |  |
| **LAIV** |  | X |  |  |  |  |  |  |  |  |  |  |  |  |  |
| **Nasal Biopsy** |  |  |  |  |  |  |  |  |  |  |  |  |  |  | X |
| Lymph node fine needle aspiration |  | X | | | | | | | | | | | | |  |
| Nasosorption ((X) home sampling) | X | X | (X) | (X) | X | (X) | (X) | X | (X) | (X) | (X) | (X) | (X) | X |  |
| Nasal cells | X |  |  |  | X |  |  | X |  |  |  |  |  | X |  |
| Nasal wash | X |  |  |  | X |  |  | X |  |  |  |  |  | X |  |
| Throat swab | X | X |  |  | X |  |  | X |  |  |  |  |  | X |  |
| Saliva ((X) home sampling) | X | X | (X) | (X) | X | (X) | (X) | X | (X) | (X) | (X) | (X) | (X) | X |  |
| Research bloods | X |  |  |  | X |  |  | X |  |  |  |  |  | X |  |

**Supplementary Table 2: Schedule of events Group B.** LAIV only, younger (18-49 years) and older (55-80 years) adults (n=7-10 each group); **^ =** Screening and baseline samples can be done on the same day or separately. If on the same day the visit window would need to be –7 to –2. If separate, screening can be from day –90 onwards up to day -2. ***Screening bloods=** FBC, U+E, LFT, HbA1c, random plasma glucose, CRP, HIV, Hep B/C, Coagulation (for nasal biopsy cohort only) **^$^** = **Safety bloods** (U+E, LFT, CRP, FBC) are only required during baseline assessment visit if screening bloods were performed >21 days beforehand. **Shaded text =** at any time between these dates depending on clinical staff and participant combined availability. ^%^ = Vital signs may be performed at the discretion of the ENT specialists. ^&^ = Safety follow-up: Participants after nasal biopsy procedure will be observed as per local NHS hospital guidelines before leaving the facility. A phone safety review will be conducted by the study team at 1 day and 3 weeks after the nasal biopsy. Participants undertaking the optional nasal biopsy will undertake a separate additional written consent for the procedure which will be performed or re-confirmed on the day of the biopsy by the operator. **Optional procedure, up to 5 participants in the older group
